# Supplementary material for: Evaluation of the Immortalized Primary Human Hepatocyte Cell Line Fa2N-4 as a Model for Metabolic Dysfunction–Associated Steatotic Liver Disease
Source: Gastro Hep Adv. 2026 Jul 22;5(11):101069. doi: 10.1016/j.gastha.2026.101069 (PMC13582011; doi:10.1016/j.gastha.2026.101069)

OVERVIEW SUPPLEMENTAL TABLE AND FIGURES

Supplementary File 1 (below)

Supplementary Table 1: Genetic variation in known MASLD single nucleotide polymorphisms (SNPs) identified in hepatic cell lines.......... 4

Supplementary Figure S1. Basal glucose concentration in HepG2 and Fa2N-4 cell media does not change intracellular triglycerides. 5

Supplementary Figure S2. Quantification of Oil red O stainings in QuPath with the StarDist module.......... 6

Supplementary Figure S3. Cell viability of HepG2 cells after 24 h treatment with resmetirom. 7

Supplementary File 2 (separate .xsls)

Table 1 - HepG2 PEA steatosis

Table 2 - Fa2N-4 PEA steatosis

Table 3 - HepG2 DEGs

Table 4 - FA2N-4 DEGs

Table 5 - DEGs HepG2 vs. Fa2N-4

Table 6 -Fa2N-4+RSM Glyc

Supplementary File 3 Karyograms (below)

Reports and original data (.pdf and .png)

**Characteristics known SNPs present in SNP array**<br>Used dbSNP NCBI[77] and literature[36–40,78]								**HepG2**<br>South American White descent, XY, 15 years				**Fa2N-4**<br>Unknown White descent, XX, 12 years			
**CHR**	**Locus**	**SNP ID**	**REF**	**ALT**	**SNP Effect**	**ALT Allele frequency** [41]	**Variant effect for MASLD**	**Result**	**Genotype**	**GT Quality**	**Risk allele**	**Result**	**Geno type**	**GT Quality**	**Risk allele**
2	*COBLL1 / GRB14*	rs13389219	C	T	Intronic variant	0.4080	Increase	Heterozygote ALT	0/1	89%	+1	Homozygote REF	0/0	93%	0
2	*GCKR*	rs780094	T	C	Intronic variant	0.7049	Increase	Heterozygote ALT	0/1	70%	+1	Heterozygote ALT	0/1	58%	+1
2	*GCKR*	rs1260326	T	C	P446L	0.7130	Increase	missing call	./.	6%	.	Heterozygote ALT	0/1	35%	+1
3	*ATG7*	rs36117895	T	C	V471A	0.0298	Increase	Heterozygote ALT	0/1	80%	+1	Homozygote REF	0/0	80%	0
3	*PPARG*	rs17036160	C	T	P12A	0.0670	Increase	Homozygote REF	0/0	83%	0	Homozygote REF	0/0	83%	0
4	*ADH1B*	rs1229984	T	C	H48R	0.8535	Decrease	Homozygote ALT	1/1	59%	0	Homozygote ALT	1/1	59%	0
4	*HSD17B13*	rs6834314	A	G	Intergenic	0.2299	Increase	Homozygote REF	0/0	70%	0	Heterozygote ALT	0/1	70%	+1
6	*HFE*	rs1800562	G	A	C282Y	0.0122	Increase	Homozygote REF	0/0	93%	0	Homozygote REF	0/0	93%	0
6	*HFE*	rs1799945	C	G	H63D	0.0739	Increase	Homozygote REF	0/0	87%	0	Homozygote REF	0/0	87%	0
6	*SIRT5*	rs12216101	T	G	Intronic	0.2174	Increase	Heterozygote ALT	0/1	36%	+1	Homozygote REF	0/0	87%	0
11	*PNPLA2*	rs140201358	C	G	N252K	0.0045	Increase	Homozygote REF	0/0	79%	0	Homozygote REF	0/0	79%	0
14	*SERPINA1*	rs28929474	C	T	E366K	0.0044	Increase	Homozygote REF	0/0	67%	0	Homozygote REF	0/0	67%	0
16	*FTO*	rs17817449	T	G	Intronic	0.3106	Increase	Homozygote REF	0/0	92%	0	Homozygote REF	0/0	37%	0
17	*APOH*	rs1801689	A	C	C325G	0.0183	Increase	Homozygote REF	0/0	76%	0	Homozygote REF	0/0	76%	0
19	*APOE*	rs429358<br><br>rs7412	T<br><br>C	C<br><br>T	C130R<br><br>R176C	0.1512<br><br>0.0771	Increase (Apo-ε3/ε3)<br><br>Decrease (Apo-ε4ε4, ε2ε4)	Homozygote REF<br><br>Homozygote REF<br>Genotype ®ε3ε3	0/0<br><br>0/0	33%<br><br>70%	0<br><br>0	Heterozygote ALT<br><br>Heterozygote ALT<br>Genotype® ε2ε4	0/1<br><br>0/1	33%<br><br>70%	+1<br><br>+1
19	*MBOAT7 / TMC4*	rs8736	C	T	3 Prime UTR Variant – Upstream variant	0.6334	Increase	Homozygote REF	0/0	69%	0	Heterozygote ALT	0/1	69%	+1
19	*MBOAT7 / TMC4*	rs626283	G	C	Downstream - Upstream variant	0.6302	Increase	Homozygote REF	0/0	93%	0	Heterozygote ALT	0/1	93%	+1
19	*MBOAT7*	rs641738	C	T	Downstream variant - G17E	0.6287	Increase	Homozygote REF	0/0	82%	0	Heterozygote ALT	0/1	82%	+1
19	*TM6SF2*	rs58542926	C	T	E167K	0.0661	Increase	Homozygote REF	0/0	89%	0	Homozygote REF	0/0	89%	0
19	*TM6SF2*	rs187429064	A	G	L156P	0.0036	Increase	Homozygote REF	0/0	79%	0	Homozygote REF	0/0	79%	0
22	*PNPLA3*	rs738409	C	G	I148M	0.2644	Increase	Homozygote ALT	1/1	83%	+2	Homozygote ALT	1/1	83%	+2
22	*PNPLA3*	rs2896019	T	G	Intron variant	0.2626	Increase	Homozygote REF	0/0	85%	0	Homozygote ALT	1/1	85%	+2
22	*PNPLA3*	rs6006460	G	T	S453I	0.0336	Decrease	Homozygote REF	0/0	87%	0	Homozygote REF	0/0	87%	0
							**Total count of present risk alleles**				**6**				**12**

Supplementary Table 1: Genetic variation in known MASLD single nucleotide polymorphisms (SNPs) identified in hepatic cell lines. A multi-ethnic global screening array with the GRCh37 reference identified genetic variations in MASLD. Information derived from dbSNP NCBI for the annotated gene names, SNP effect, and alternative allele frequency based on 1000Genomes_30X Global[41]. The total count of risk alleles was calculated by the presence of disease-risk associated SNPs, amounting to +1 per present risk allele (in some cases, the reference allele is the risk allele, in other cases, an alternative). Abbreviations used: REF, reference allele; ALT, alternative allele; AF, allele frequency (of reference allele).

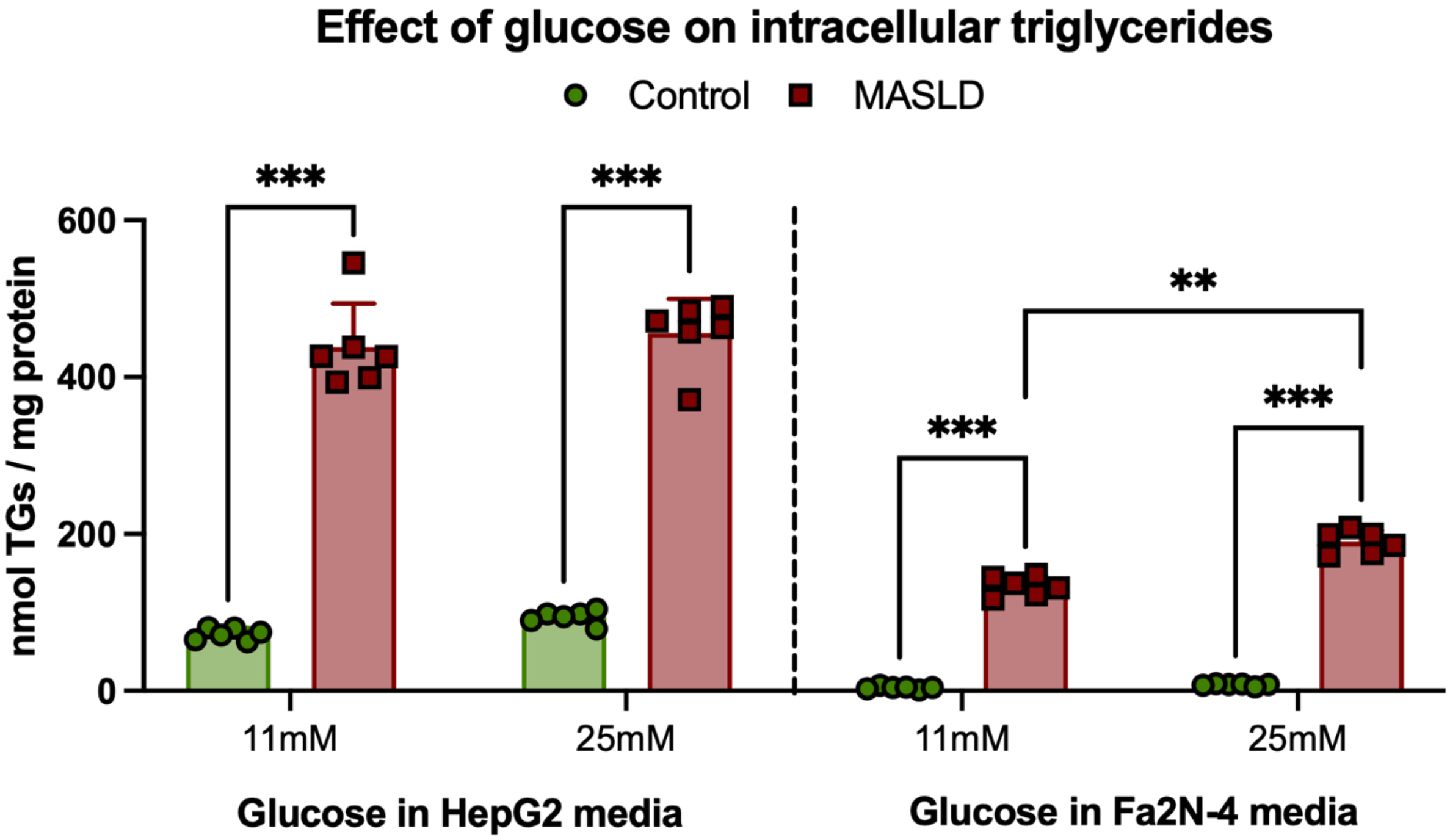

Supplementary Figure S1. Basal glucose concentration in HepG2 and Fa2N-4 cell media does not change intracellular triglycerides. Intracellular triglyceride levels of HepG2 and Fa2N-4 cells upon culturing in 11 mM and 25 mM glucose. 2-Way ANOVA test with *** $p < 0.001$ or ** $p < 0.01$ indicated.

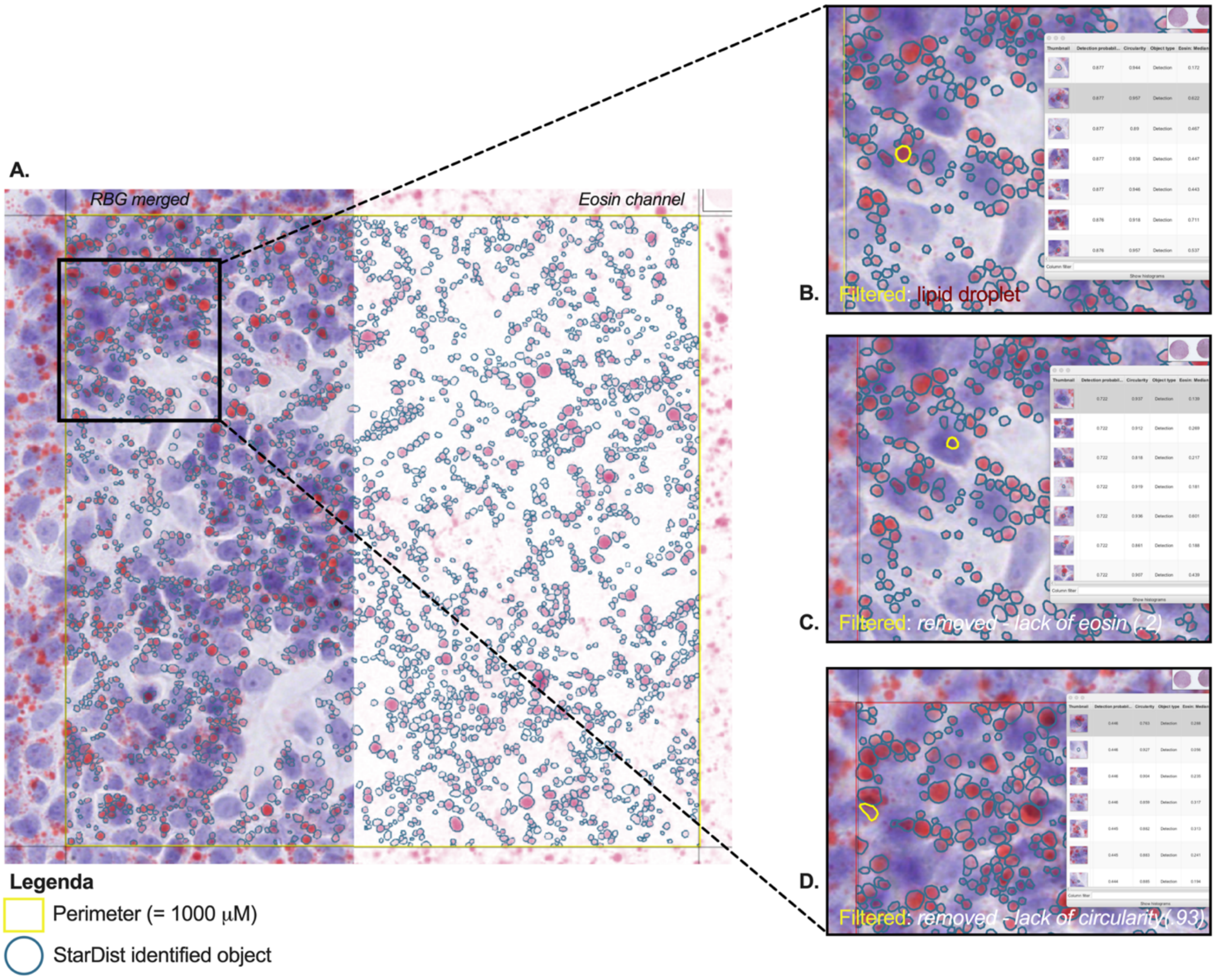

Supplementary Figure S2. Quantification of Oil red O stainings in QuPath with the StarDist module. (A) Representative image taken from a slide with steatotic HepG2 cells. On the right side, the merged RGB image is shown. On the left, the Eosin-only channel is displayed. (B) Example of an object identified as lipid droplet after applying filters on detection probability, circularity, and eosin channel. (C) Example of an object removed by filtering based on the lack of a threshold in the eosin channel of 0.2 and above. (D) Example of object removed by filtering based on lack of circularity threshold of 0.93 and above. Not shown is detection probability inclusion of ≥ 0.75.

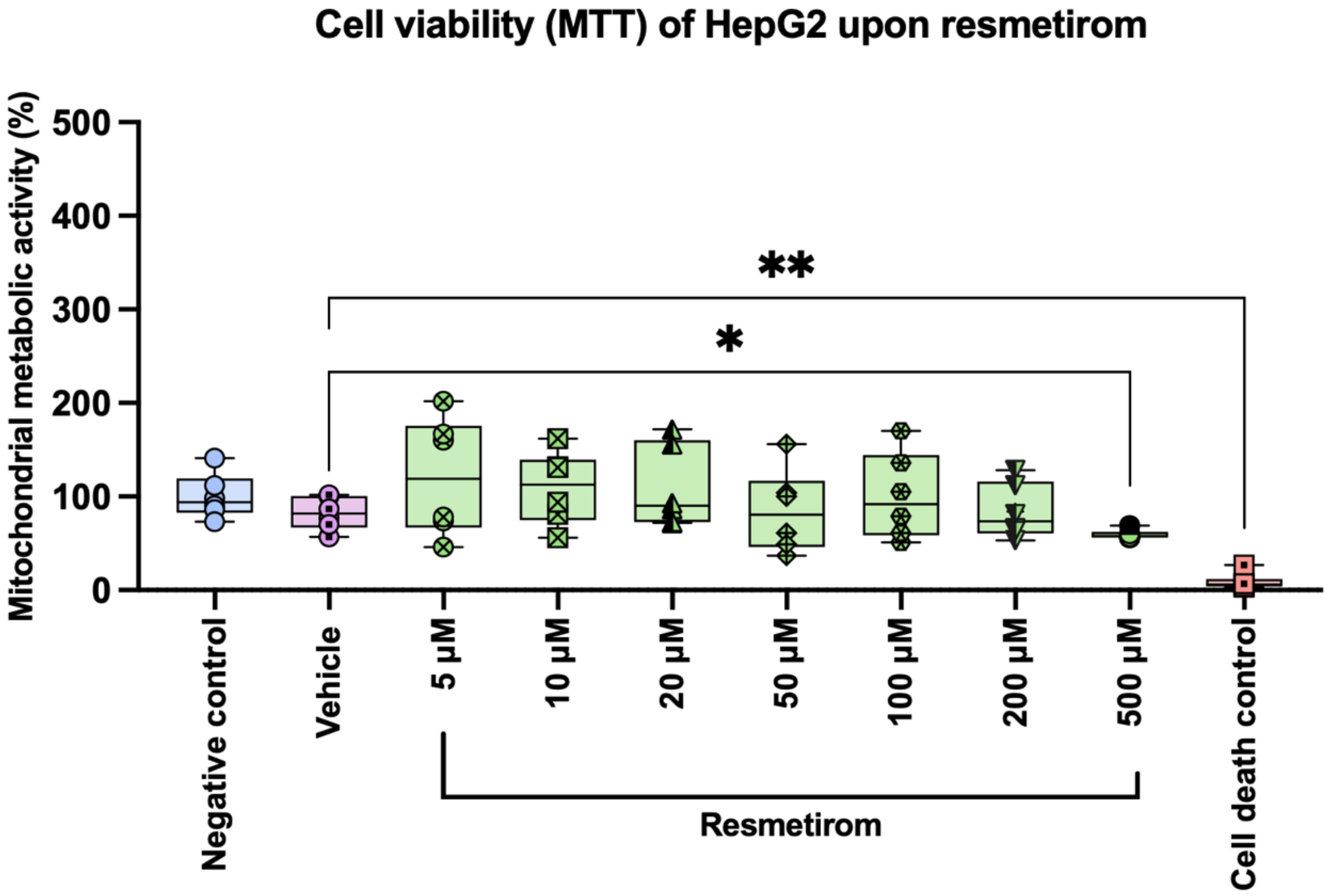

Supplementary Figure S3. Cell viability of HepG2 cells after 24 h treatment with resmetirom. Cell viability of HepG2 cells upon treatment with different resmetirom concentrations was examined by an MMT assay. Mann-Whitney test was performed, with * $p < 0.05$ and ** $p < 0.01$.

Case: HepG2

**Patient ID:**	**HepG2 p19**	**Operator Name:**	
**Patient Name:**		**Specimen:**	
**Date of Birth:**		**Received Date:**	**16-1-2025**
**Gender:**		**Completed:**	
**Clinical Indication:**			

Case Notes: **Analysis: 26-3-2025**

Case Results: **51~52,XY,t(1;21)(p22;p11),+2,+16,add(16)(p13),+17,?add(17)(p11.2),+20,+20,+mar[cp10]**

Final Results:

Microscope ID	

Cells Information:

#	Label	Slide-Cell:	Analysis State	X , Y	State By	Report	Notes	Results
1	>dgn	4-17	Karyotyped	18,64 X 8,66	EV	None		51,XY,t(1;21)(p22;p11),+2,9q-,+16,add(16)(p13),+17,?add(17)(p11.2)+20,+20,-21,+mar
2	>dgn	4-19	Karyotyped	18,04 X 8,96	EV	None		51,?X,Y,t(1;21)(p22;p11),+16,,add(16)(p13),+17,?add(17)9p11.2),+20,+20
3	>dgn	4-20	Karyotyped	10,63 X 9,15	EV	None		51,XY,t(1;21)(p22;p11),+2,+16,add(16)(p13),+17,?add(17)(p11.2)+20,+20,-21,+mar
4	>dgn	4-24	Karyotyped	17,18 X 10,18	EV	None		52,XY,t(1;21)(p22;p11),+2,+16,add(16)(p13),+17,?add(17)(p11.2),+20,+20,+mar
5	>dgn	5-7	Karyotyped	18,56 X 5,43	EV	None		52,XY,t(1;21)(p22;p11),+2,add(3)(p26),+16,add(16)(p13),+17,?add(17)(p11.2),+20,+20,+mar
6	>dgn	5-11	Karyotyped	3,62 X 6,12	EV	None		51,XY,t(1;21)(p22;p11),+2,+16,add(16)(p13),+17,?add(17)(p11.2),+20,+mar
7	>dgn	5-36	Karyotyped	2,25 X 23,34	EV	None		52,XY,t(1;21)(p22;p11),+2,+16,add(16)(p13),+17,?add(17)(p11.2),+20,+20,+mar
8	>dgn	5-37	Karyotyped	9,96 X 24,11	EV	None		52,XY,t(1;21)(p22;p11),+2,+16,add(16)(p13),del(16)(p11.2),+17,?add(17)p11),+20,+20,+mar
9	>dgn	6-26	Karyotyped	13,34 X 12,21	EV	None		52,XY,t(1;21)(p22;p11),+2,+16,add(16)(p13),+17,+?add(17)(p11.2),+20,+20,+mar
10	>dgn	6-28	Karyotyped	1,82 X 13,30	EV	None		50,XY,t(1;21)(p22;p11),+2,der(2)t(2;5)(q;q),-5,-13,+20,+20,+2mar

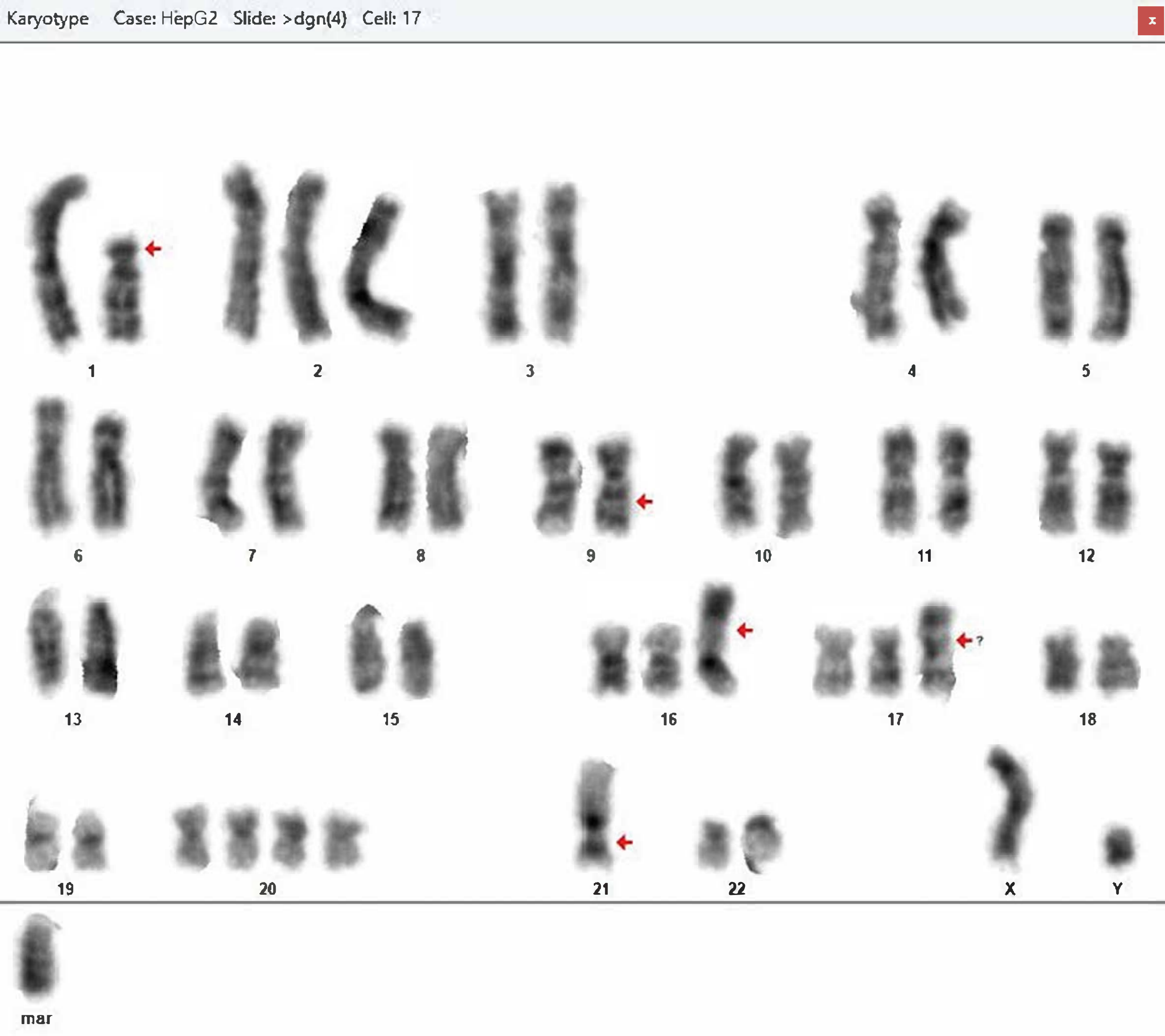
Karyotype Case: HepG2 Slide: >dgn(4) Cell: 17
x
1
2
3
4
5
6
7
8
9
10
11
12
13
14
15
16
17
?
18
19
20
21
22
X
Y
mar

Karyotype Case: HepG2 Slide: >dgn(4) Cell: 19

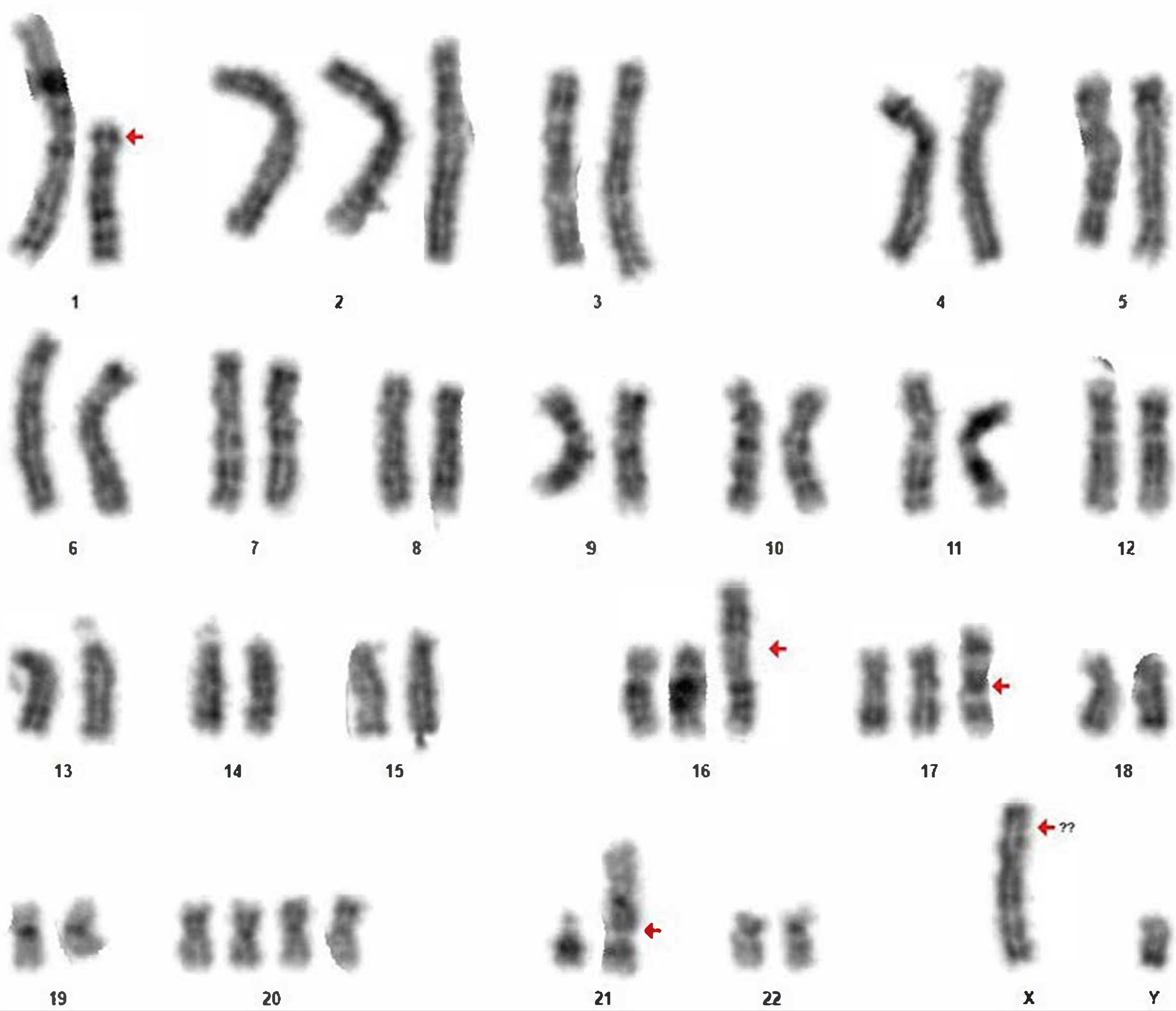

Karyotype Case: HepG2 Slide: >dgn(4) Cell: 20

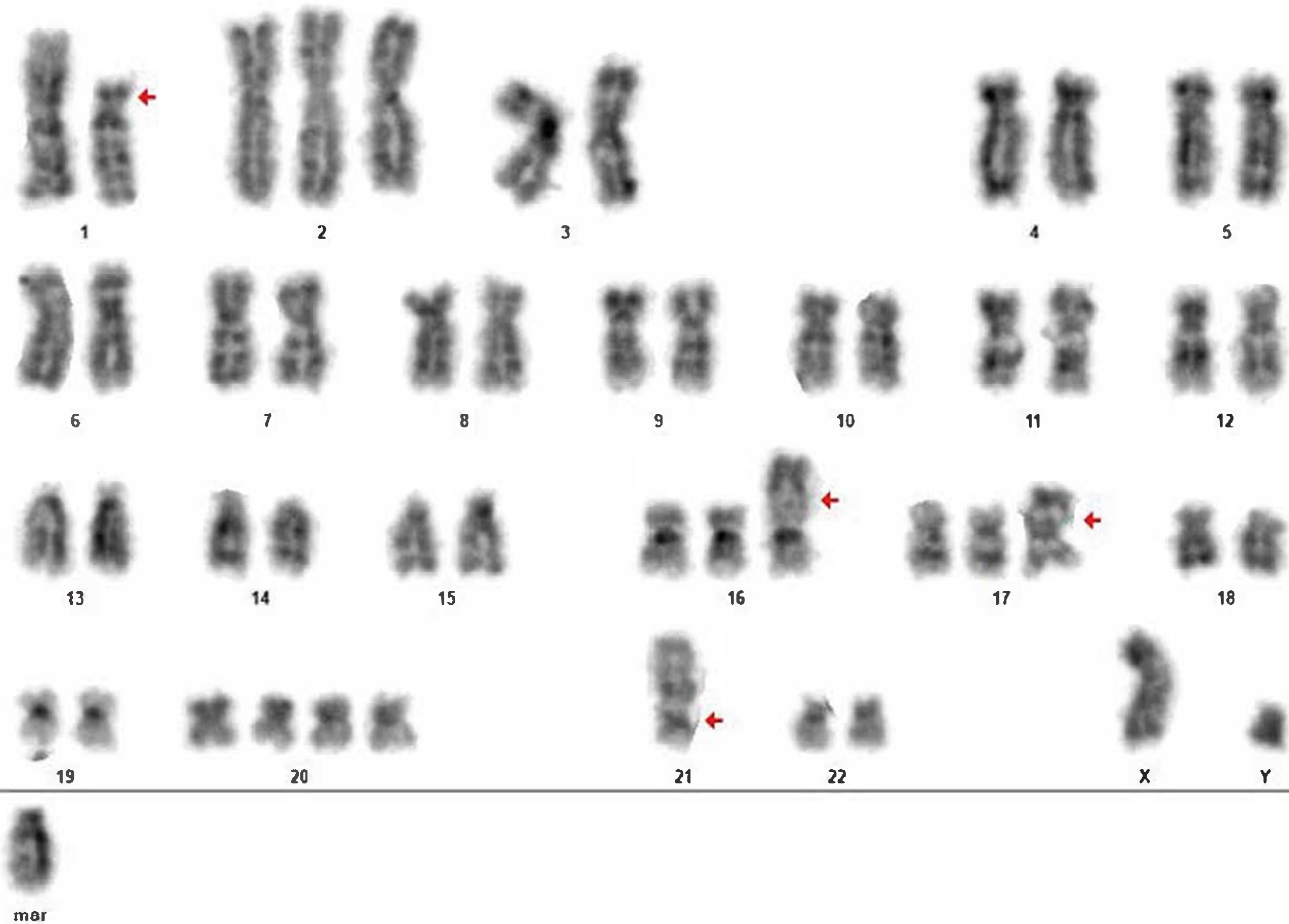

Karyotype Case: HepG2 Slide: >dgn(4) Cell: 24

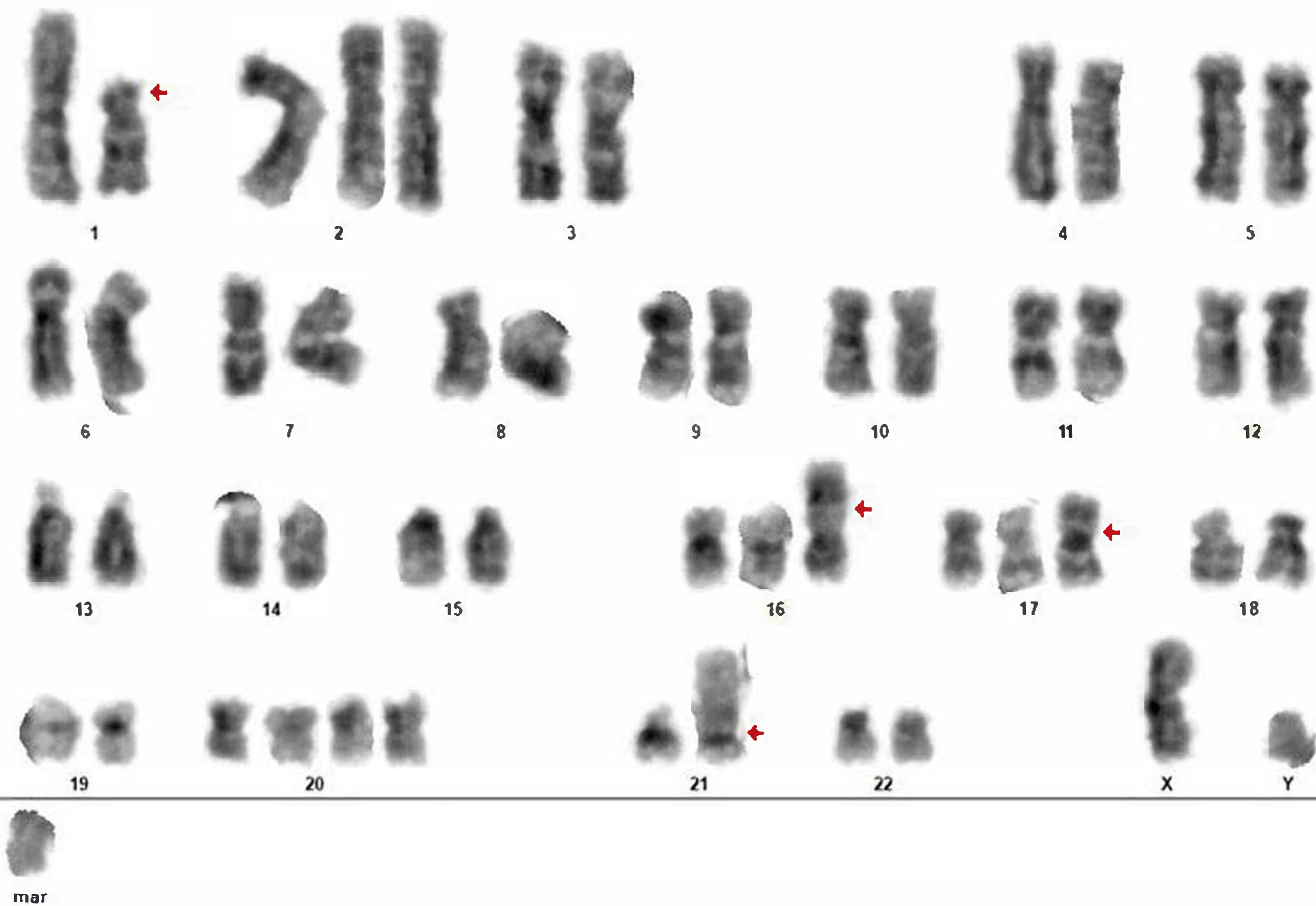

Karyotype Case: HepG2 Slide: >dgn(5) Cell: 7

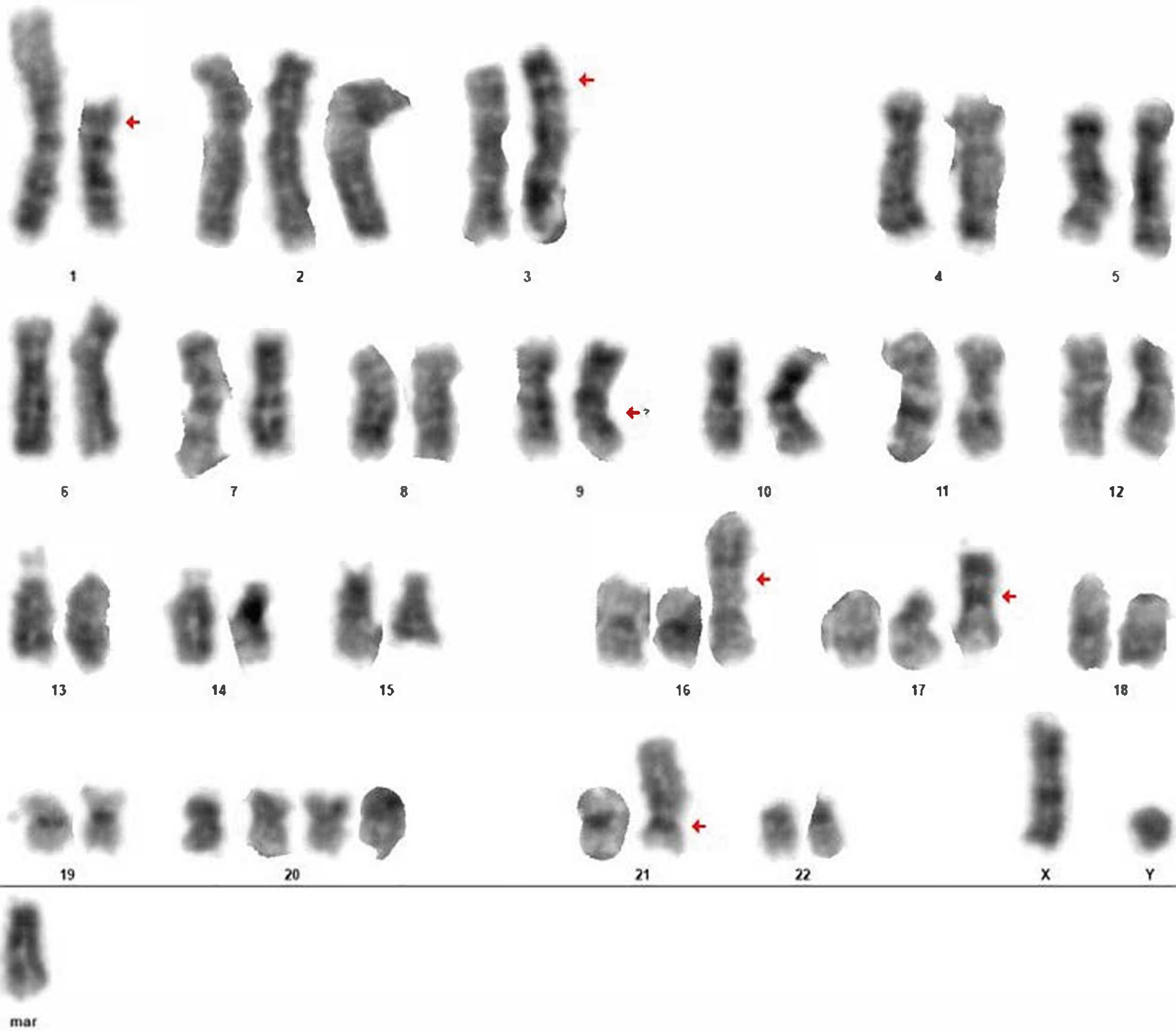

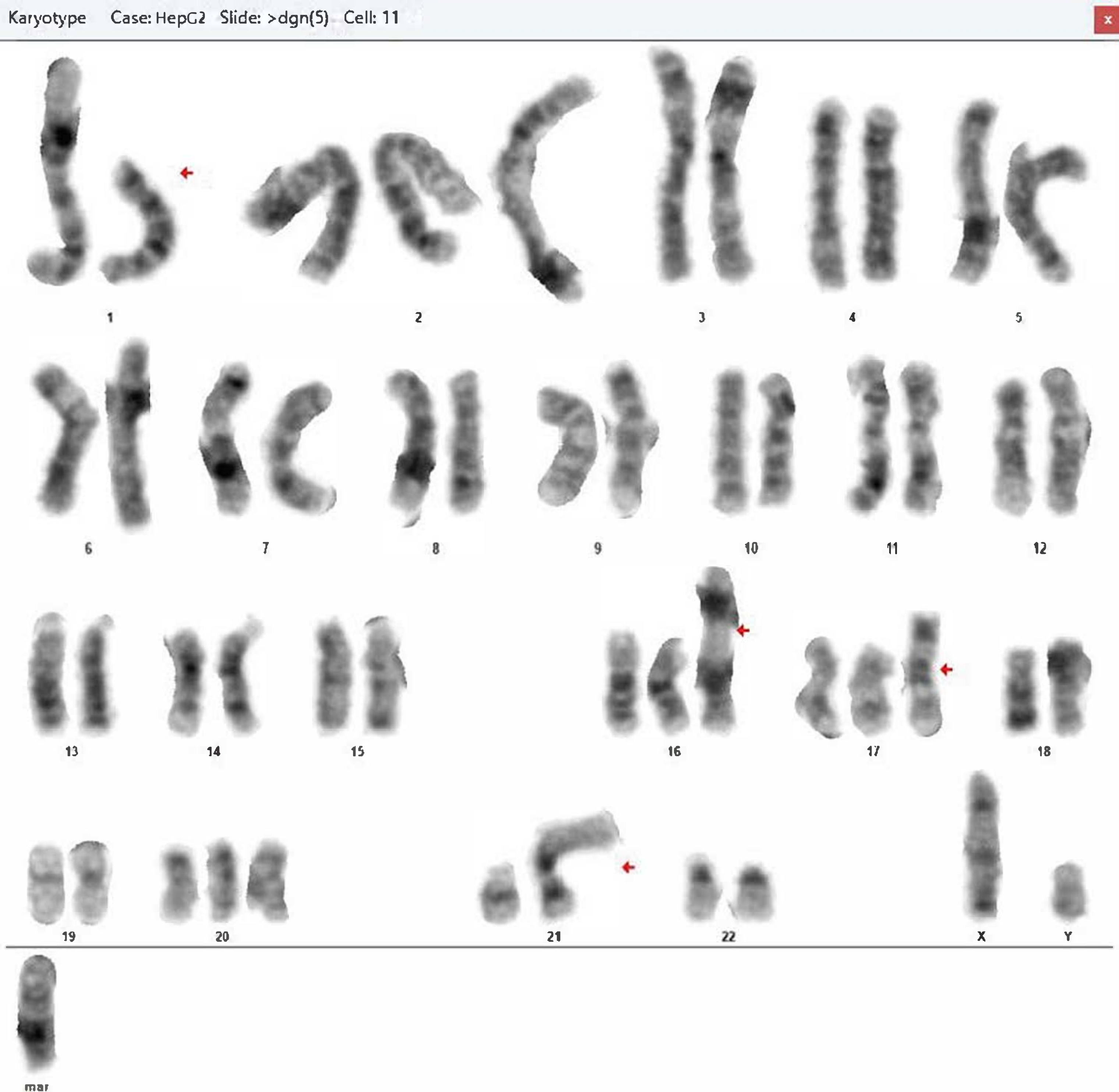
Karyotype Case: HepG2 Slide: >dgn(5) Cell: 11
1
2
3
4
5
6
7
8
9
10
11
12
13
14
15
16
17
18
19
20
21
22
X
Y
mar

Karyotype Case: HepG2 Slide: >dgn(5) Cell: 36

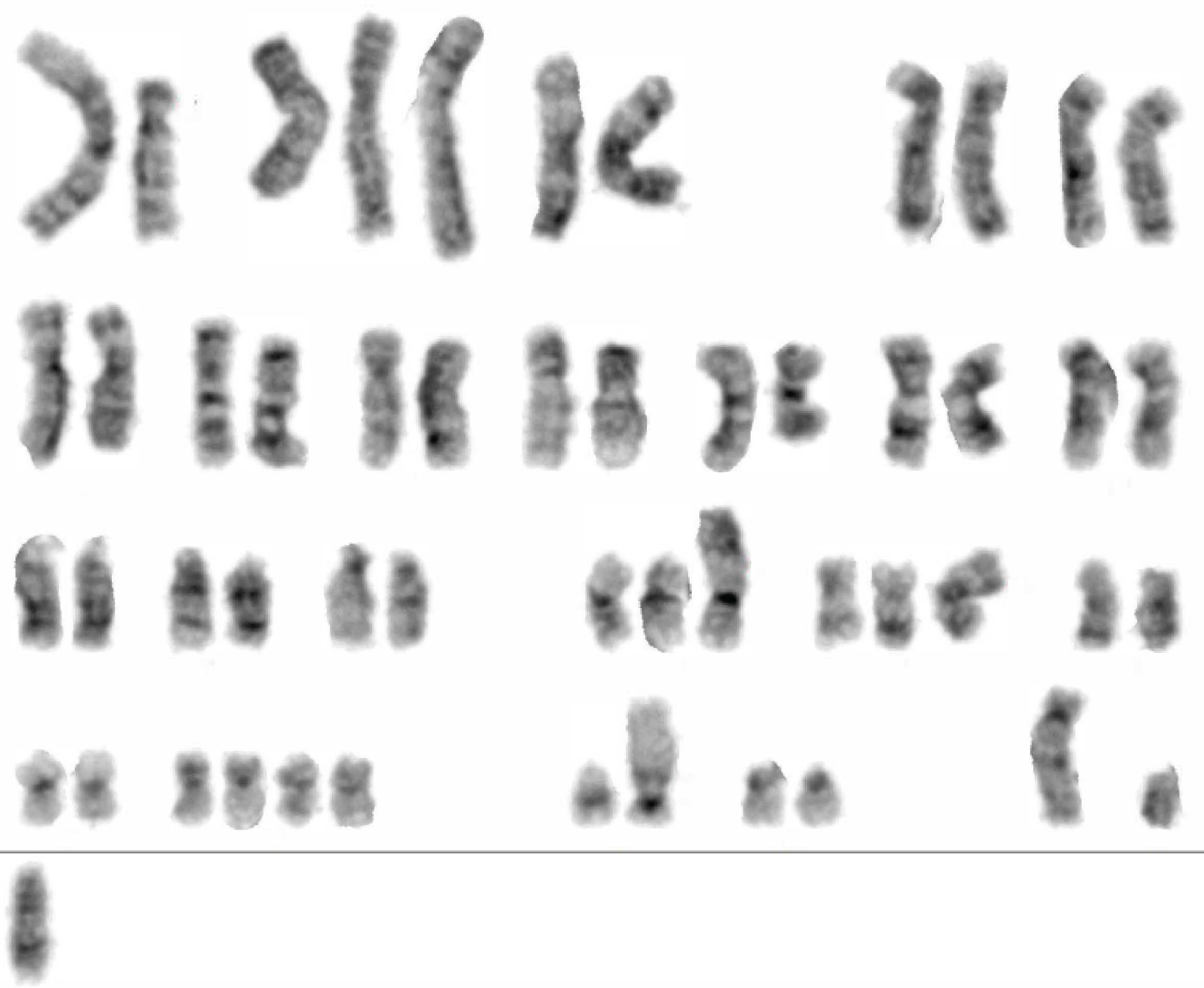

Karyotype Case: HepG2 Slide: >dgn(5) Cell: 36

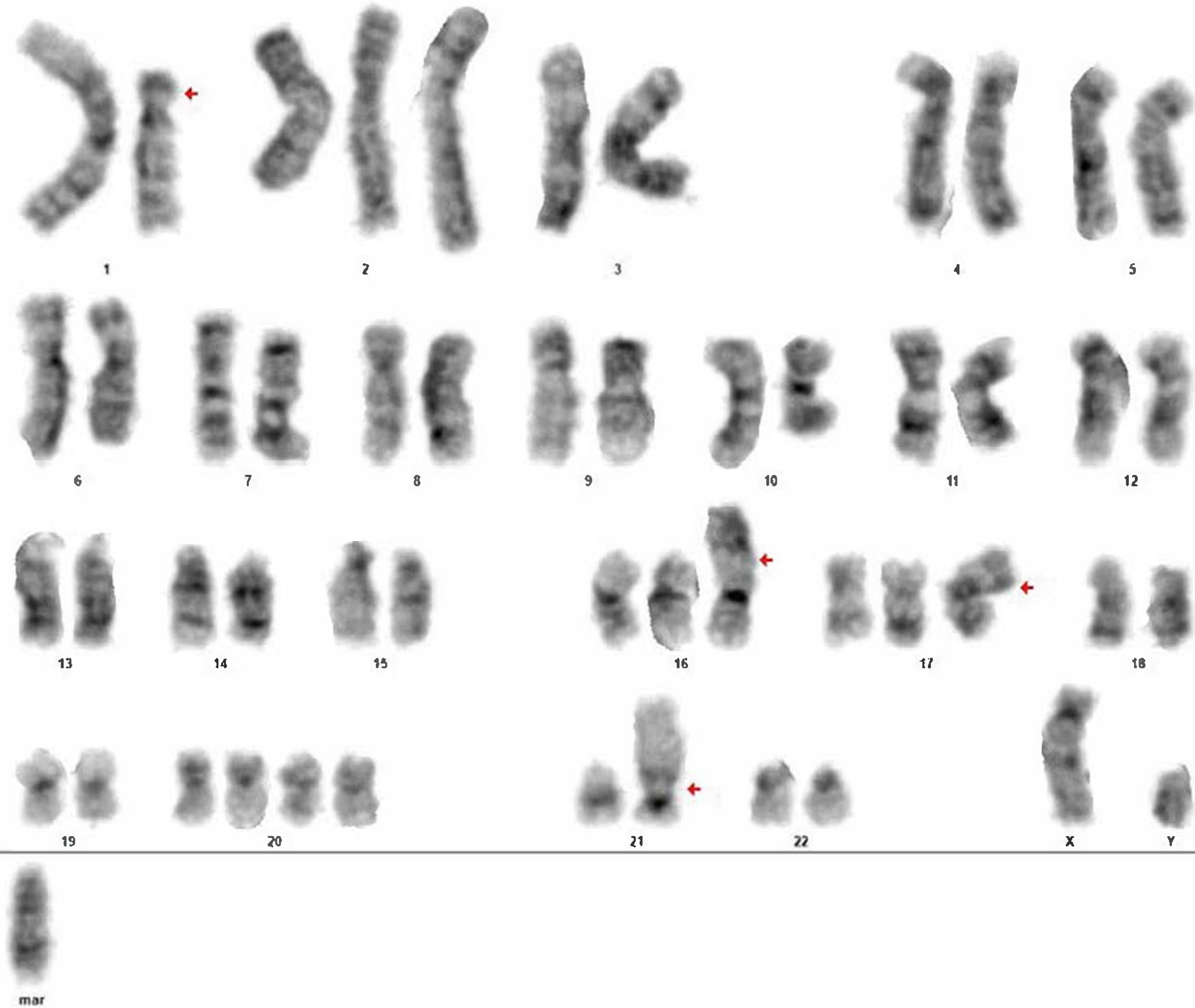

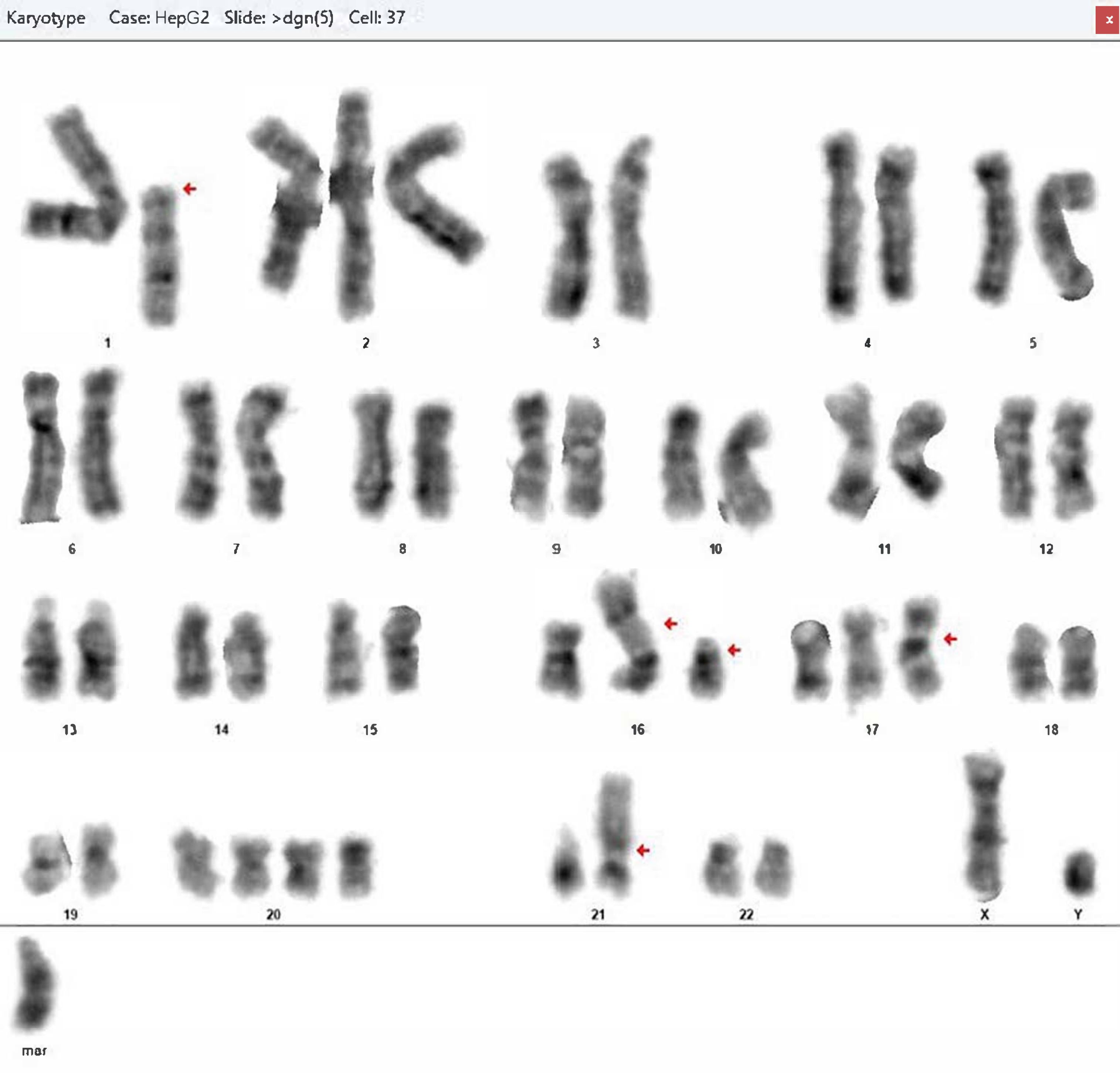
Karyotype Case: HepG2 Slide: >dgn(5) Cell: 37
1
2
3
4
5
6
7
8
9
10
11
12
13
14
15
16
17
18
19
20
21
22
X
Y
mar

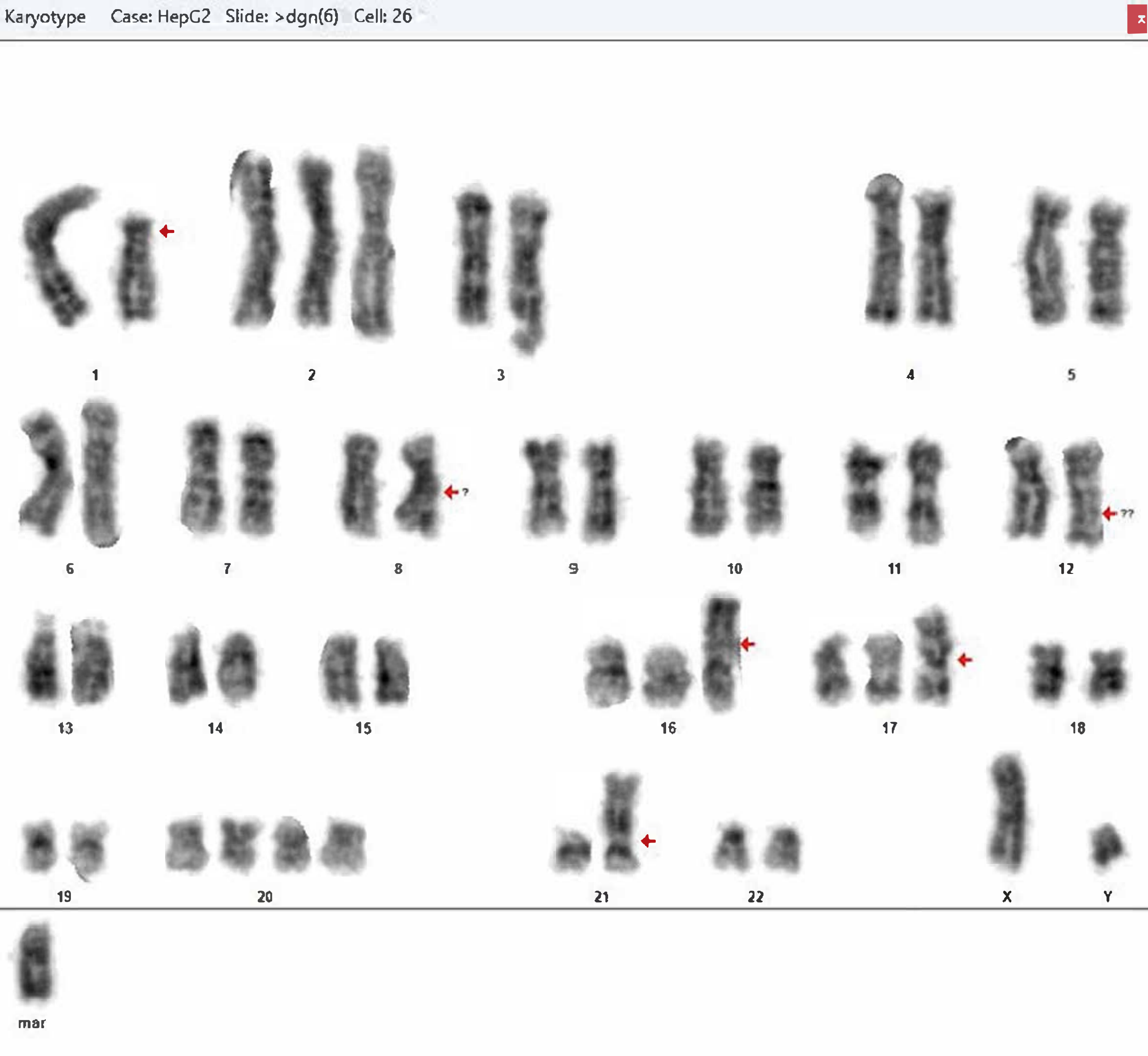

Karyotype Case: HepG2 Slide: >dgn(6) Cell: 26
1
2
3
4
5
6
7
8
?
9
10
11
12
??
13
14
15
16
17
18
19
20
21
22
X
Y
mar

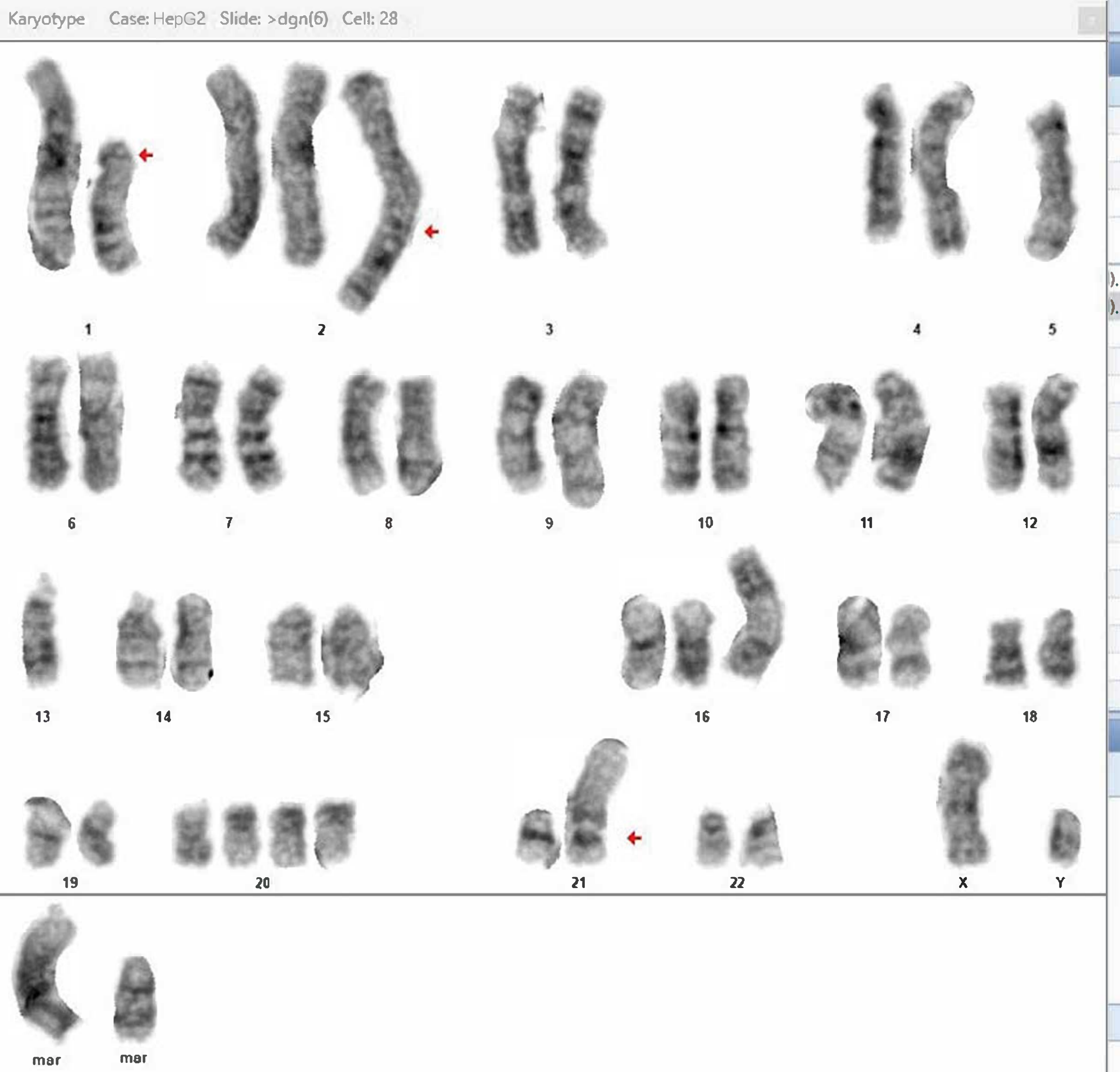

Karyotype
Case: HepG2
Slide: >dgn(6)
Cell: 28
1
2
3
4
5
6
7
8
9
10
11
12
13
14
15
16
17
18
19
20
21
22
X
Y
mar
mar

Case: Fa2N4

Patient ID: FA2N4

Patient Name: Ellen-Victoria

Date of Birth:

Gender:

Clinical Indication:

Operator Name:

Specimen:

Received Date: 16-1-2025

Completed:

Case Notes: Analysis: 28-3-2025
mar appears to be 20

Case Results:
line I:
43,X,-X,add(8)(p2?1),der(9;11)(q10;q10),-10,-13,-22,+2mar[cp3]

line II:
41~45,X,-X,add(8)(p2?1),-9,-10,del(11)(p11.2),-13,+17,-22,-22,+2mar[cp7]

Final Results:

Microscope ID	

Cells Information:

#	Label	Slide-Cell:	Analysis State	X , Y	State By	Report	Notes	Results
1	24h	2-9	Karyotyped	20,58 X 21,46	EV	None		II 45,XX,?4,add(8)(p2?1),-9,-10,del(11)(p11.2),-13,+17,-22,+2mar
2	24h	2-12	Karyotyped	11,36 X 25,70	EV	None		I 43,X,-X,add(8)(p2?1),der(9;11)(q10;q10),-10,-13,-22,+2mar
3	24h	2-13	Karyotyped	5,88 X 34,66	EV	None		I 43,XX,add(8)(p2?1),der(9;11)(q10;q10),-10,-13,-22,+mar
4	24h	2-16	Karyotyped	19,68 X 36,10	EV	None		II 43,XX,add(8)(p2?1),-10,add(11)(p11.2),add(12)([p12),-13,-18,-20,-22,+2mar
5	24h	2-25	Karyotyped	7,00 X 49,39	EV	None		II 41,XX,-8,add(8)(p2?1),-9,-10,del(11)(p11.2),-13,-19,-22,+mar
6	24h	7-2	Karyotyped	9,14 X 7,74	EV	None		II 42,X,-X,add(8)(p2?1),-9,-10,del(11)(p11.2),-13,-22,-22,+2mar
7	24h	7-18	Karyotyped	3,44 X 25,45	EV	None		II 43,X,-X,add(8)(p2?1),-9,-10,del(11)(p11.2),-13,+17,-22,-22,+2mar
8	24h	7-20	Karyotyped	8,22 X 32,32	EV	None		II 42,XX,add(8)(p2?1),-9,-10,del(11)(p11.2)-13,-16,-22,-22,+2mar
9	24h	7-24	Karyotyped	4,65 X 45,12	EV	None		I 43,X,-X,add(8)(p2?1),der(9;11)(q10;q10),-10,-13,-22,+2mar
10	24h	7-25	Karyotyped	4,31 X 45,09	EV	None		II 44,XX,add(8)(p2?1),-9,-10,del(11)(p11.2),-13,-22,+2mar

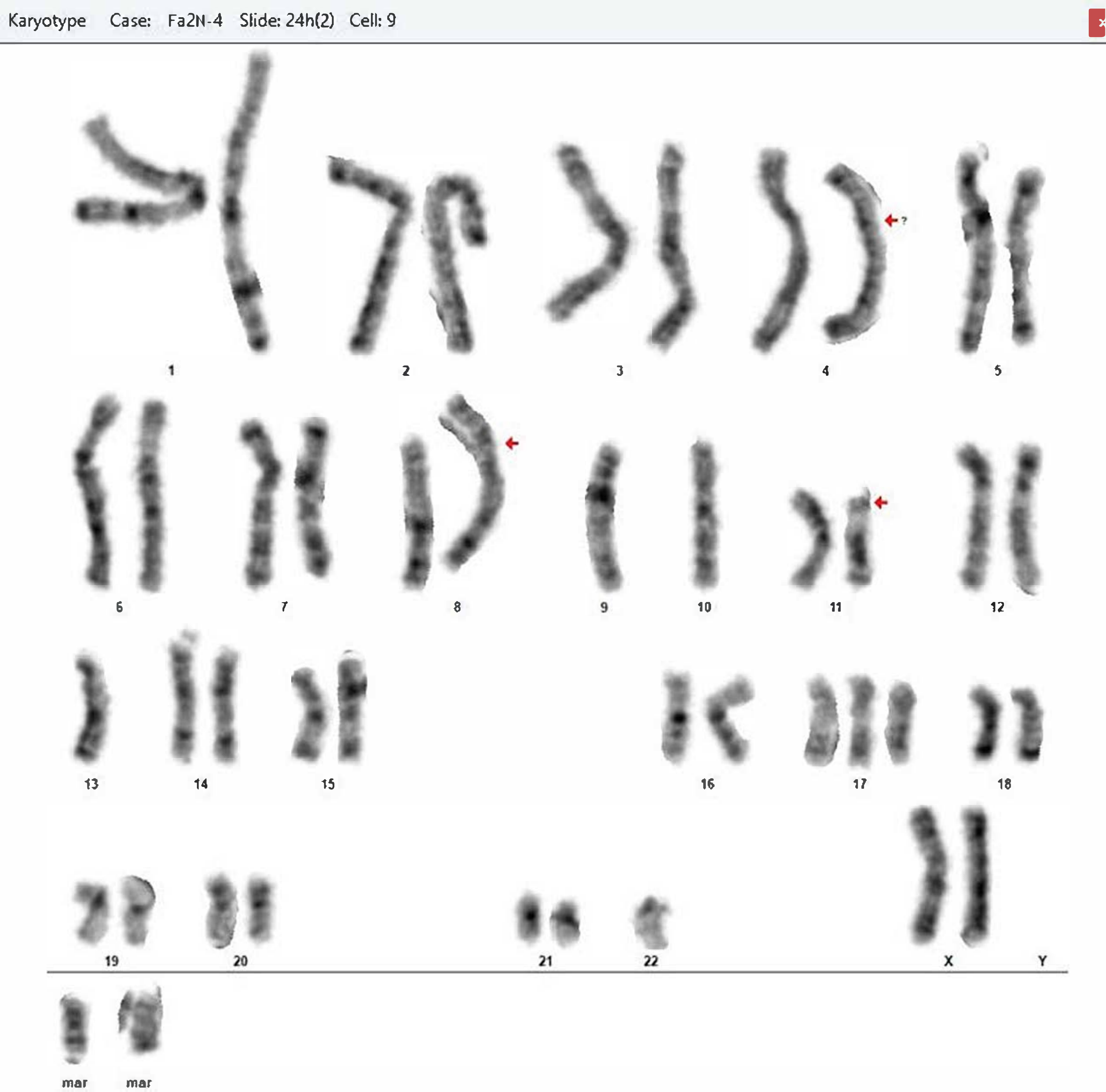
Karyotype Case: Fa2N-4 Slide: 24h(2) Cell: 9
1
2
3
4
?
5
6
7
8
9
10
11
12
13
14
15
16
17
18
19
20
21
22
X
Y
mar
mar

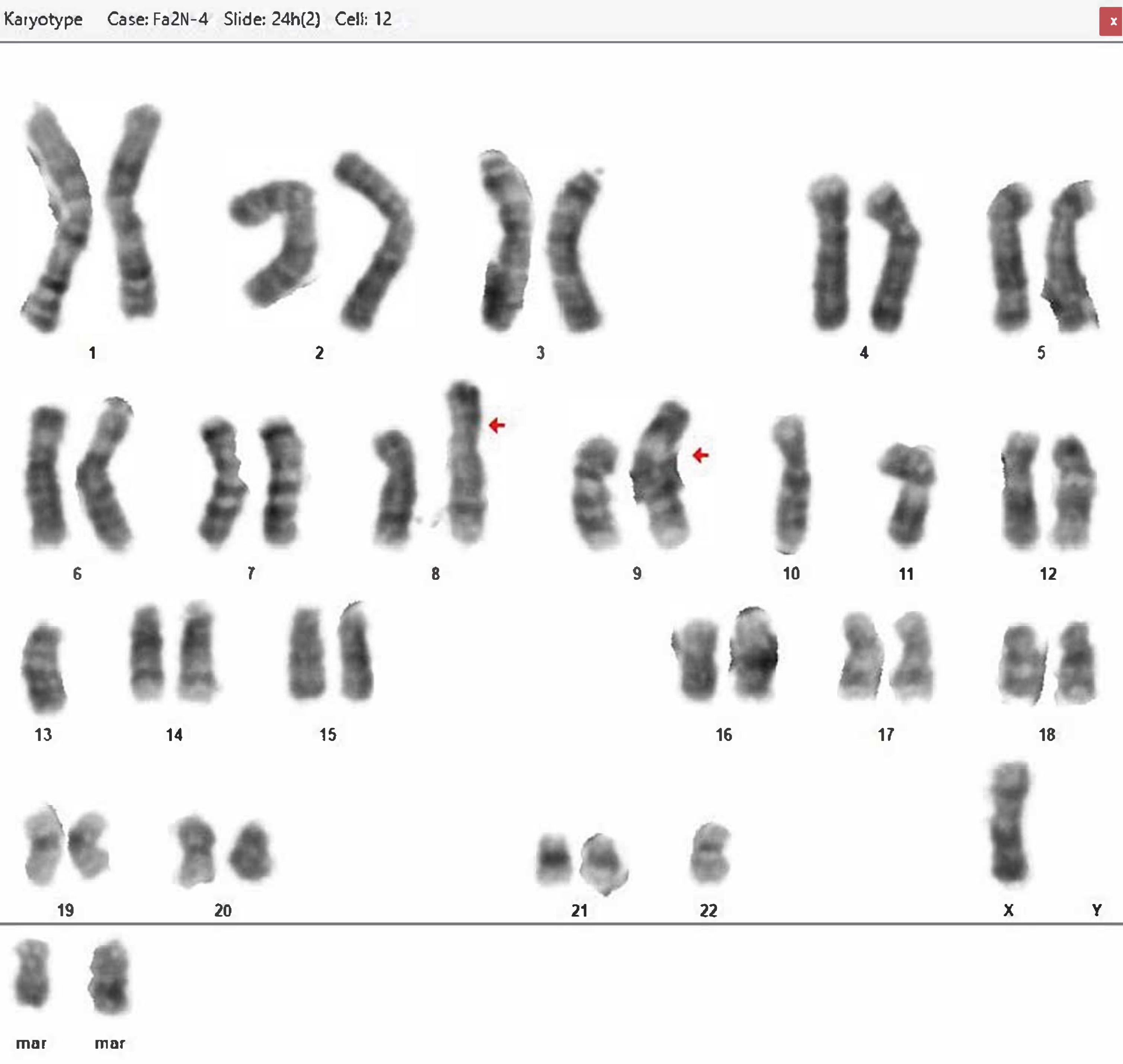
Karyotype Case: Fa2N-4 Slide: 24h(2) Cell: 12
1
2
3
4
5
6
7
8
9
10
11
12
13
14
15
16
17
18
19
20
21
22
X
Y
mar
mar

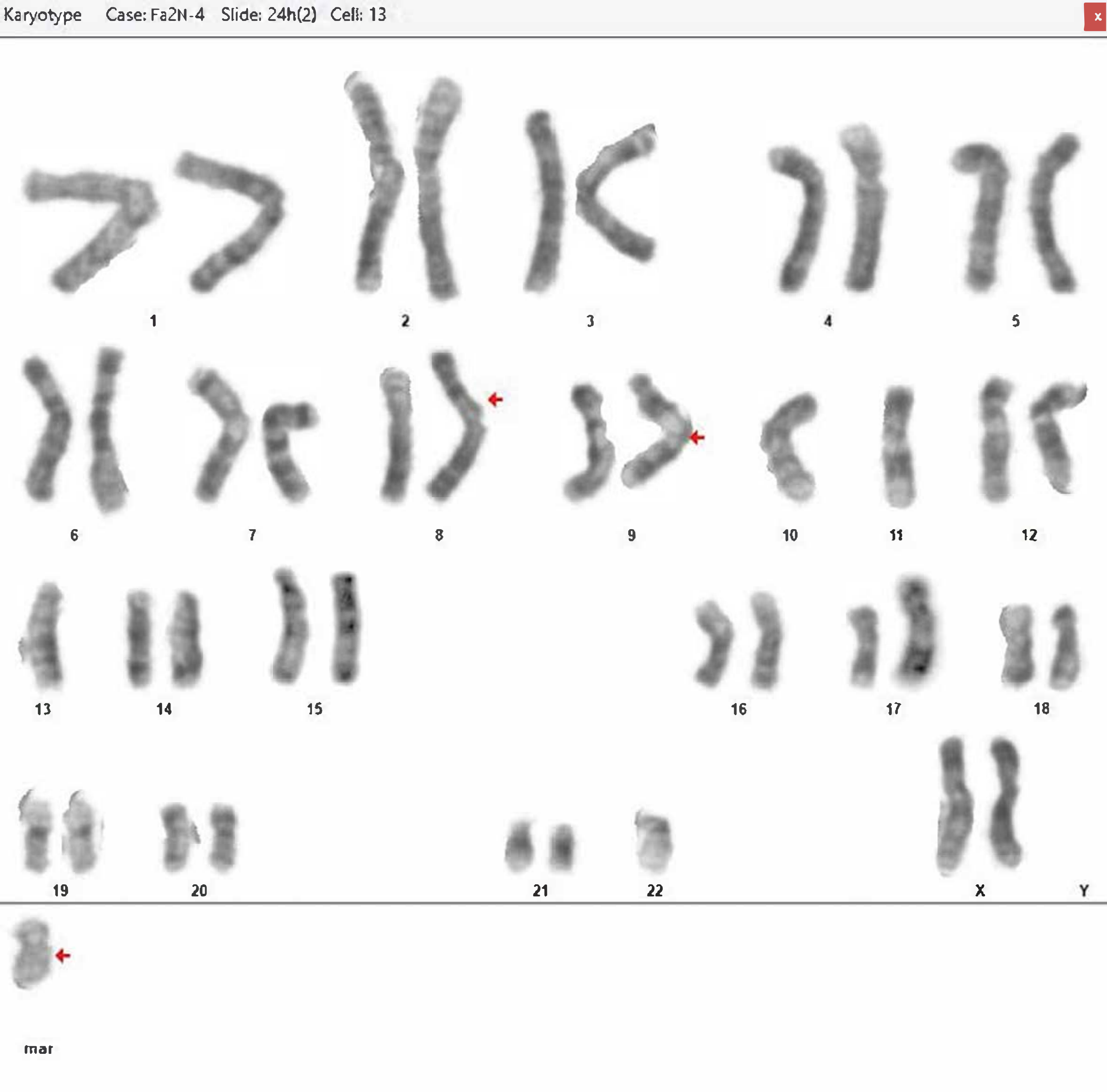
Karyotype Case: Fa2N-4 Slide: 24h(2) Cell: 13
1
2
3
4
5
6
7
8
9
10
11
12
13
14
15
16
17
18
19
20
21
22
X
Y
mar

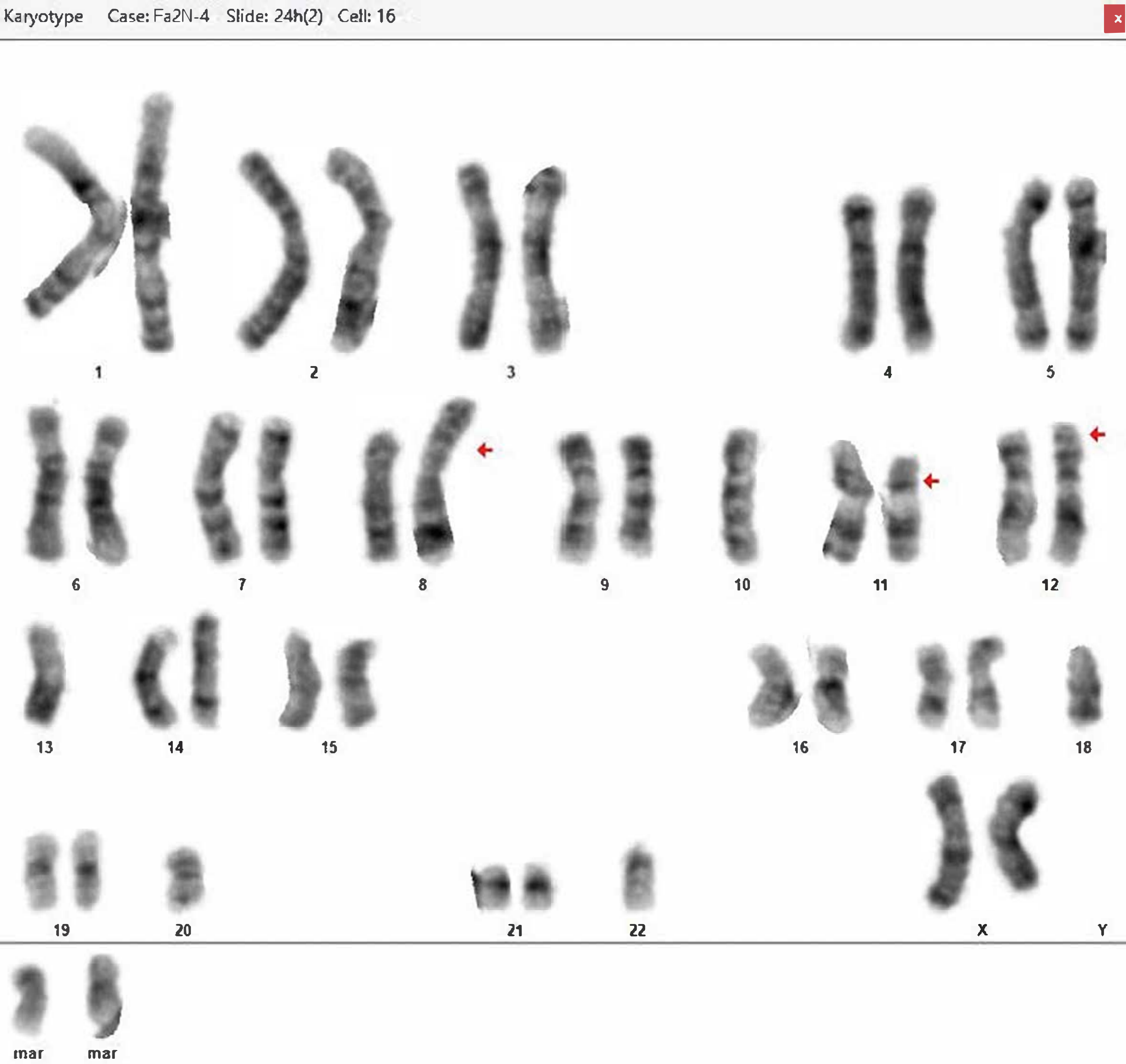
Karyotype Case: Fa2N-4 Slide: 24h(2) Cell: 16
1
2
3
4
5
6
7
8
9
10
11
12
13
14
15
16
17
18
19
20
21
22
X
Y
mar
mar

Karyotype Case: Fa2N-4 Slide: 24h(2) Cell: 25

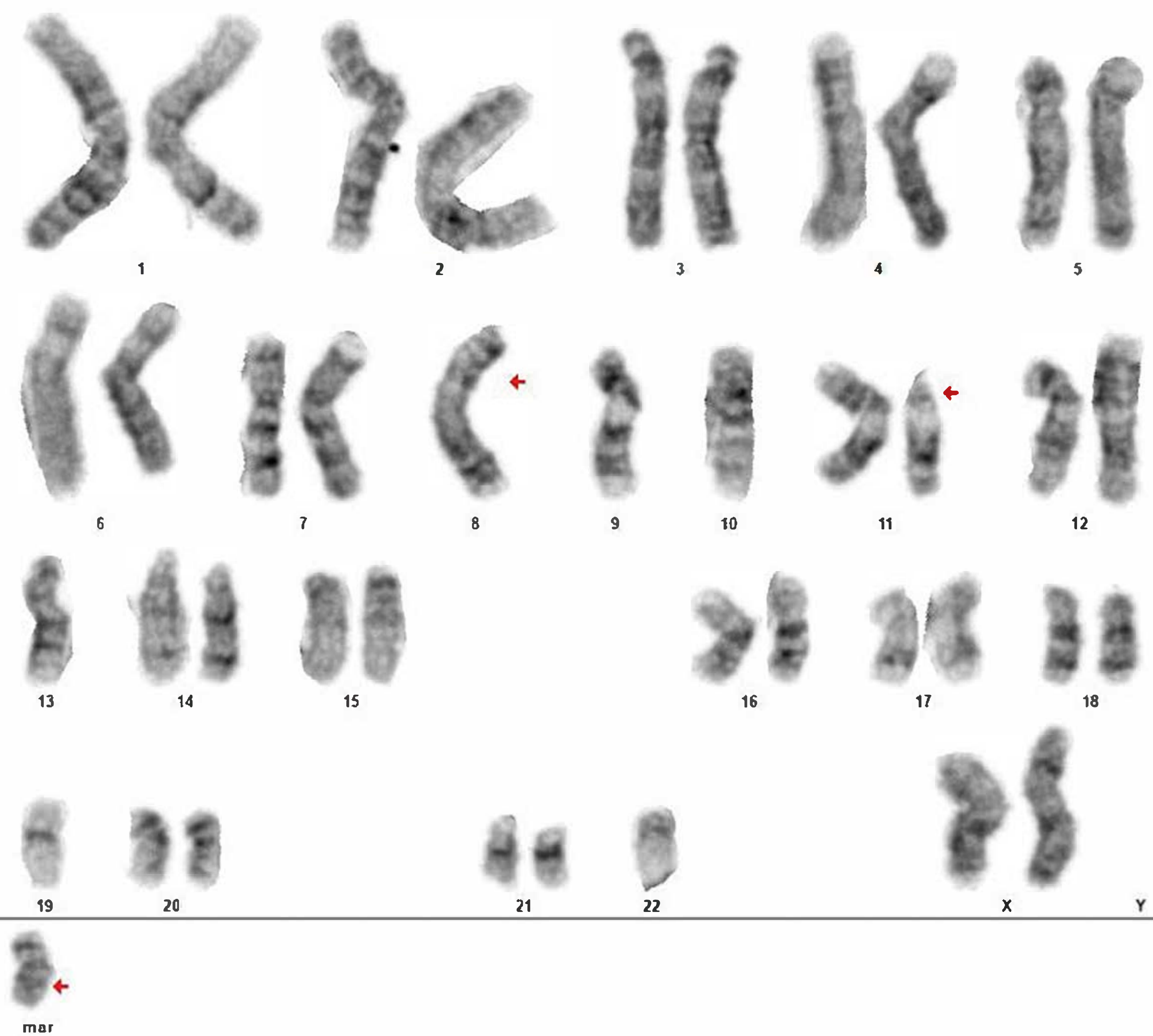

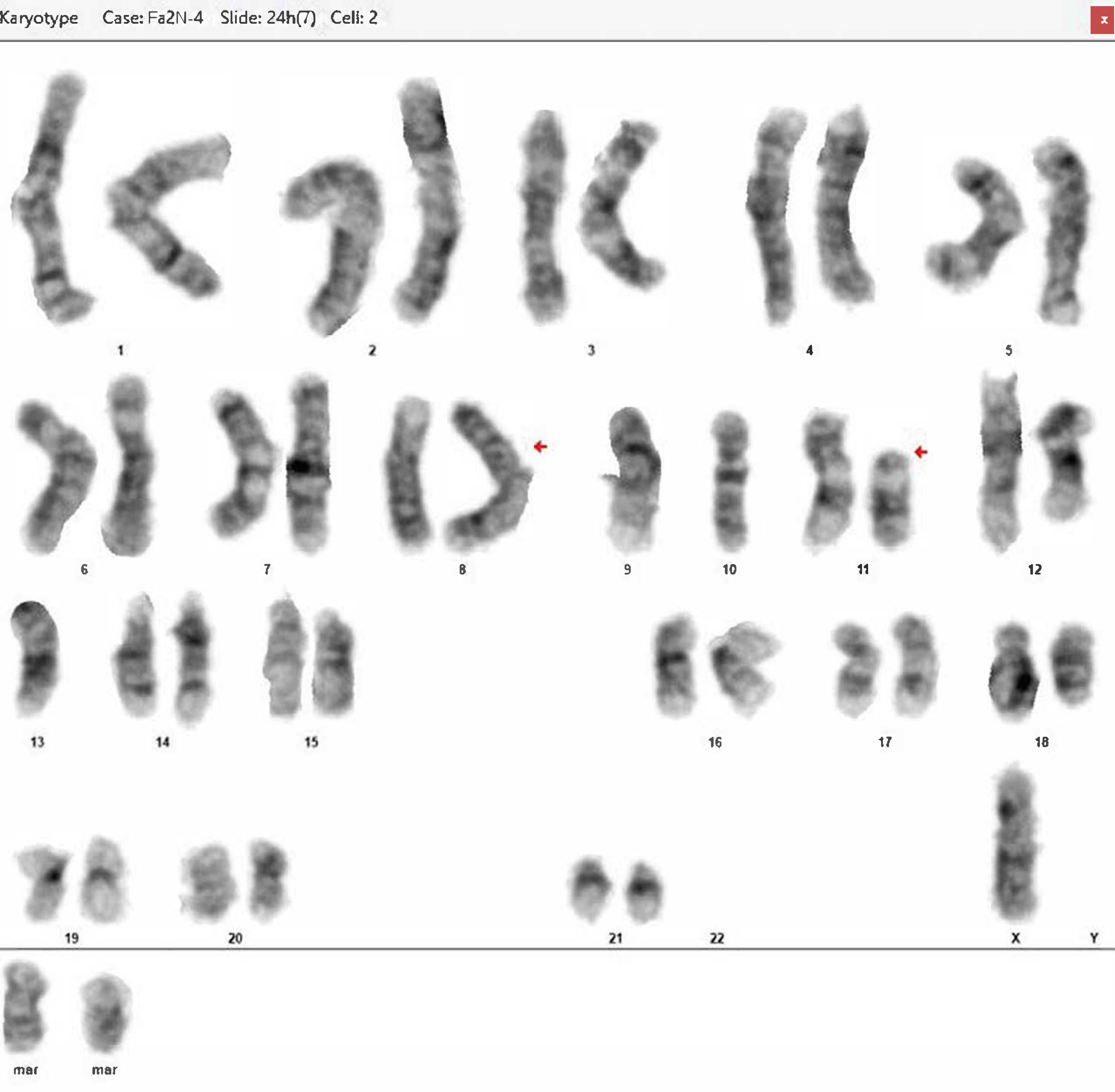
Karyotype Case: Fa2N-4 Slide: 24h(7) Cell: 2
1
2
3
4
5
6
7
8
9
10
11
12
13
14
15
16
17
18
19
20
21
22
X
Y
mar
mar

Karyotype Case: Fa2N 4 Slide: 24h(7) Cell: 18

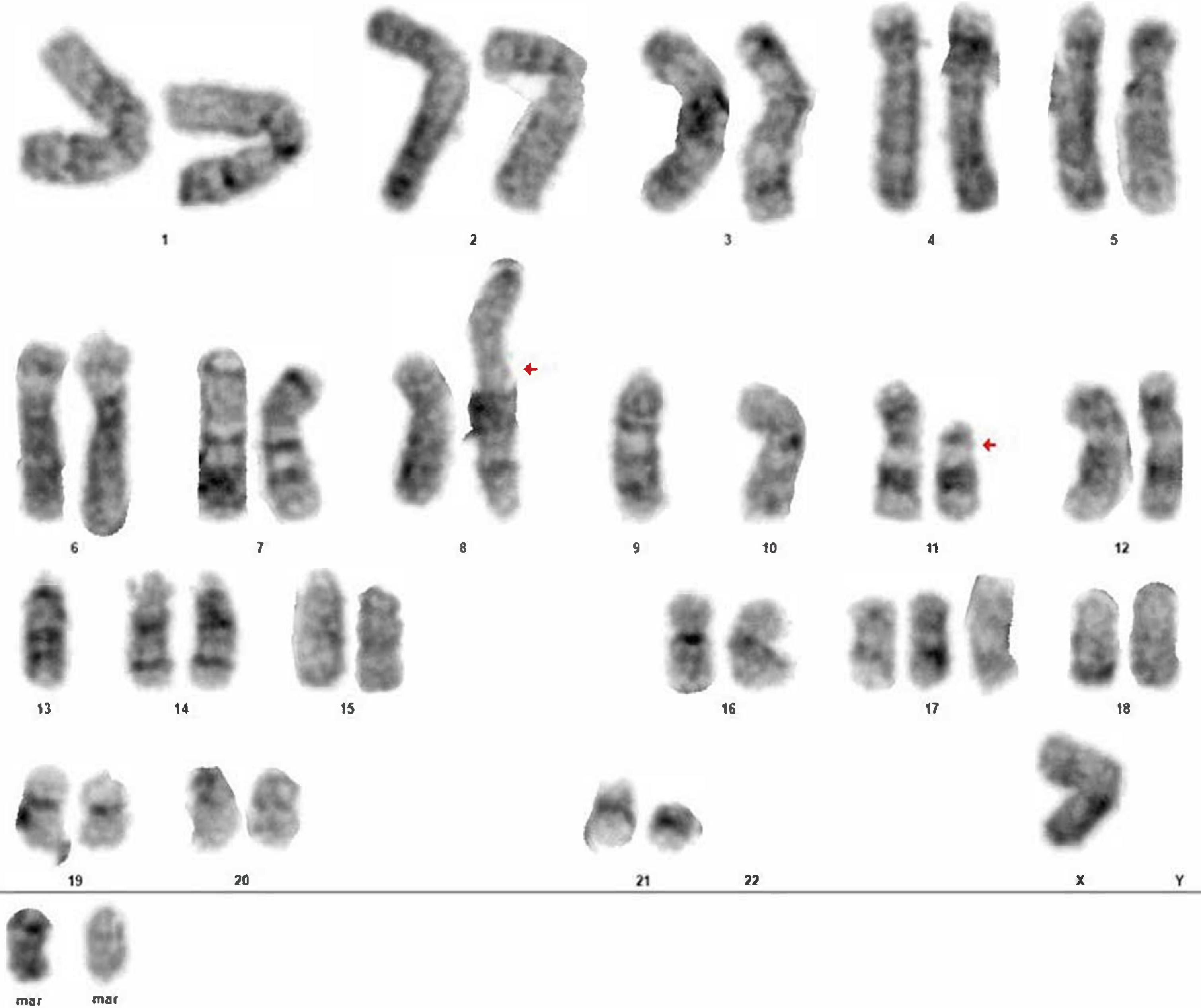

Karyotype Case: Fa2N-4 Slide: 24h(7) Cell: 20

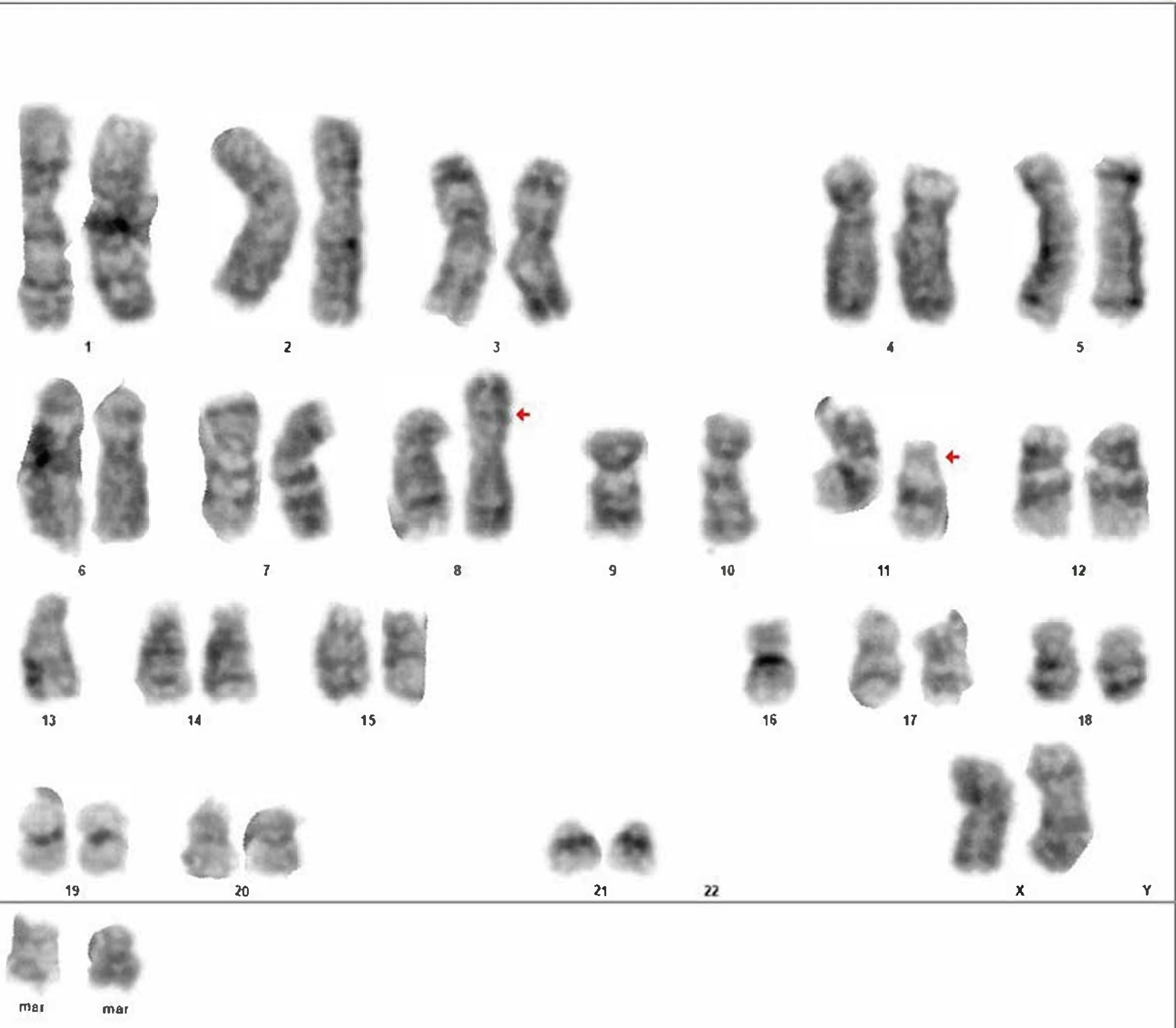

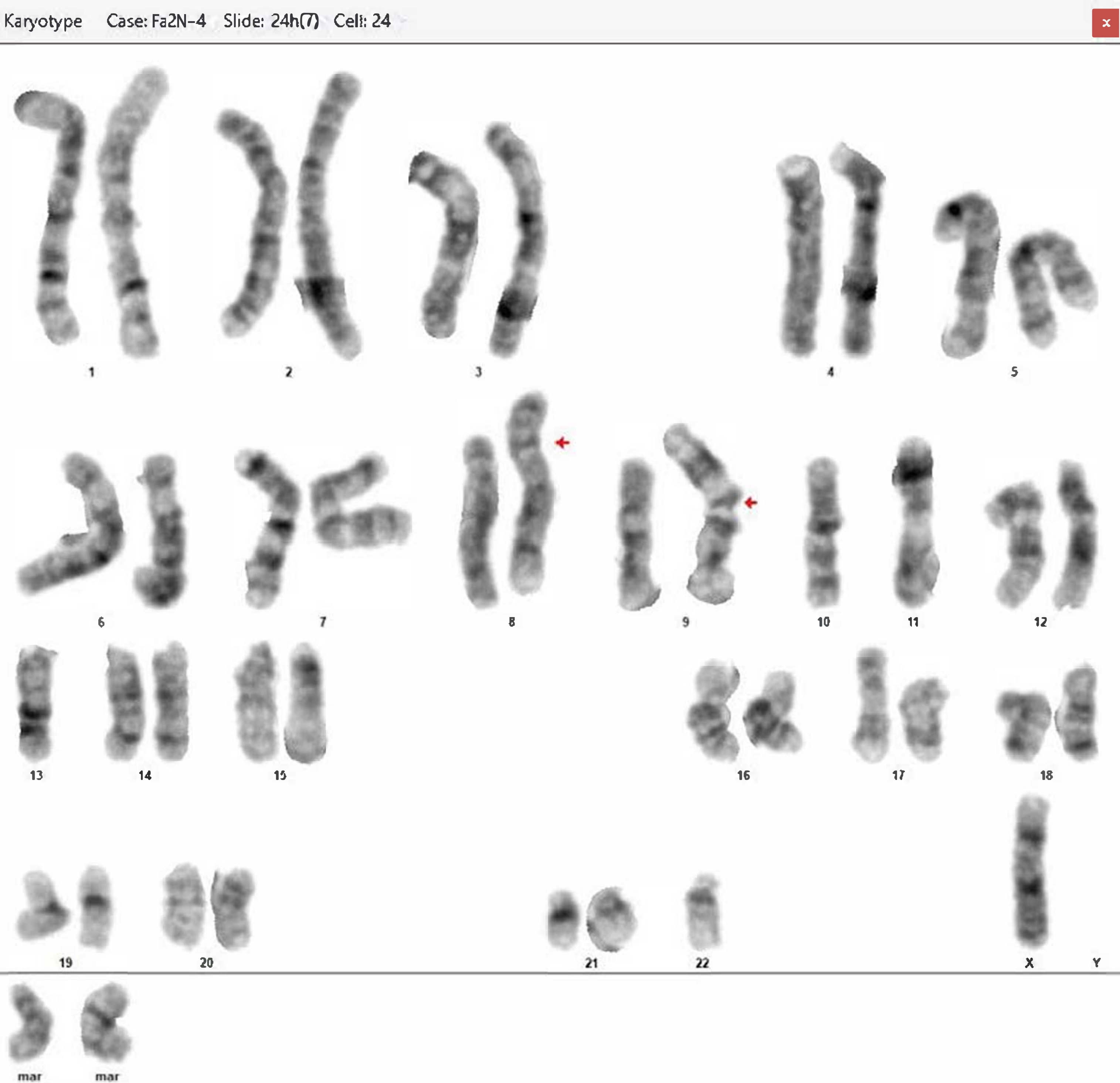
Karyotype Case: Fa2N-4 Slide: 24h(7) Cell: 24
1
2
3
4
5
6
7
8
9
10
11
12
13
14
15
16
17
18
19
20
21
22
X
Y
mar
mar

Karyotype Case: Fa2N-4 Slide: 24h(7) Cell: 25

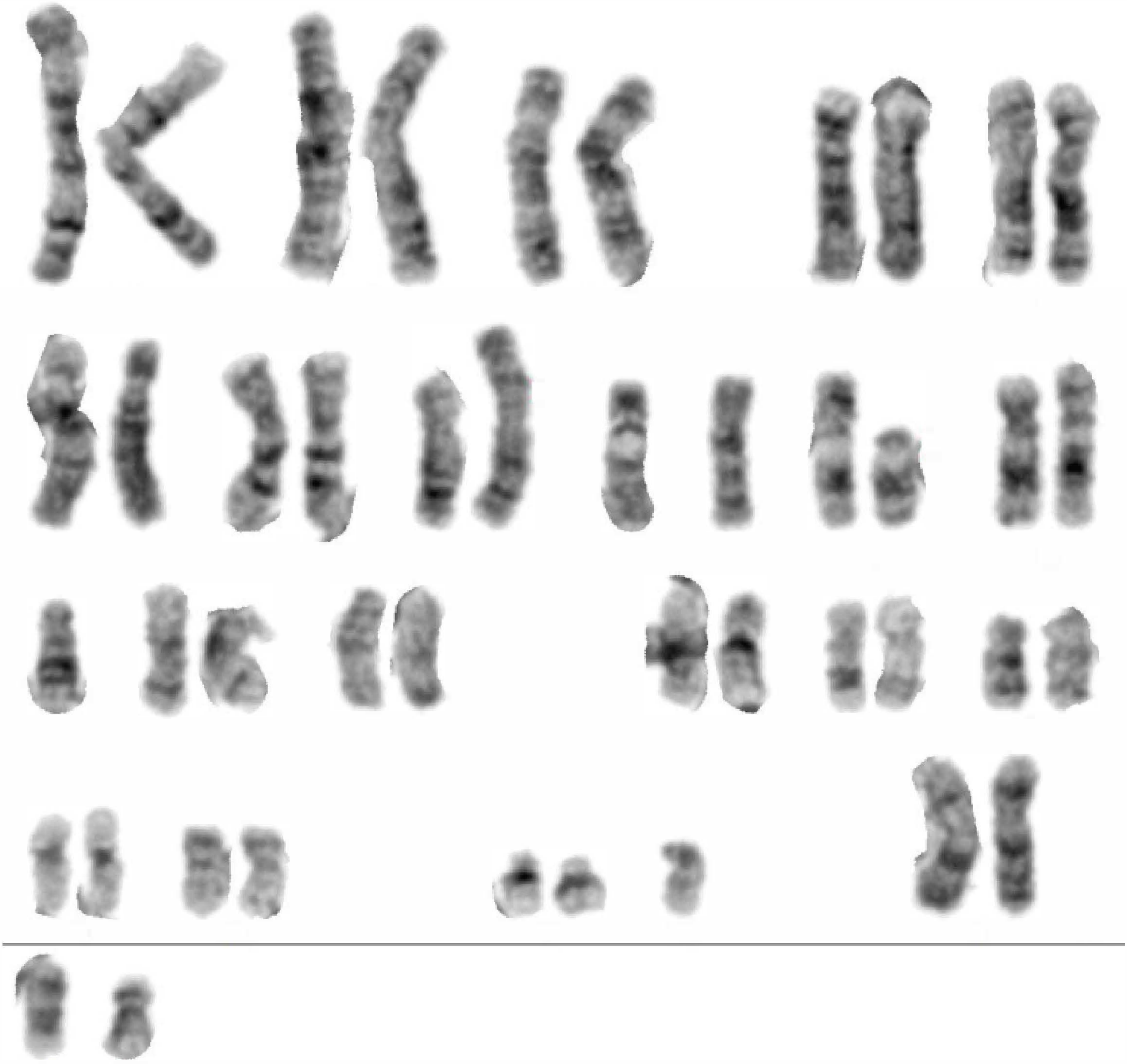

Karyotype Case: Fa2N-4 Slide: 24h(7) Cell: 25

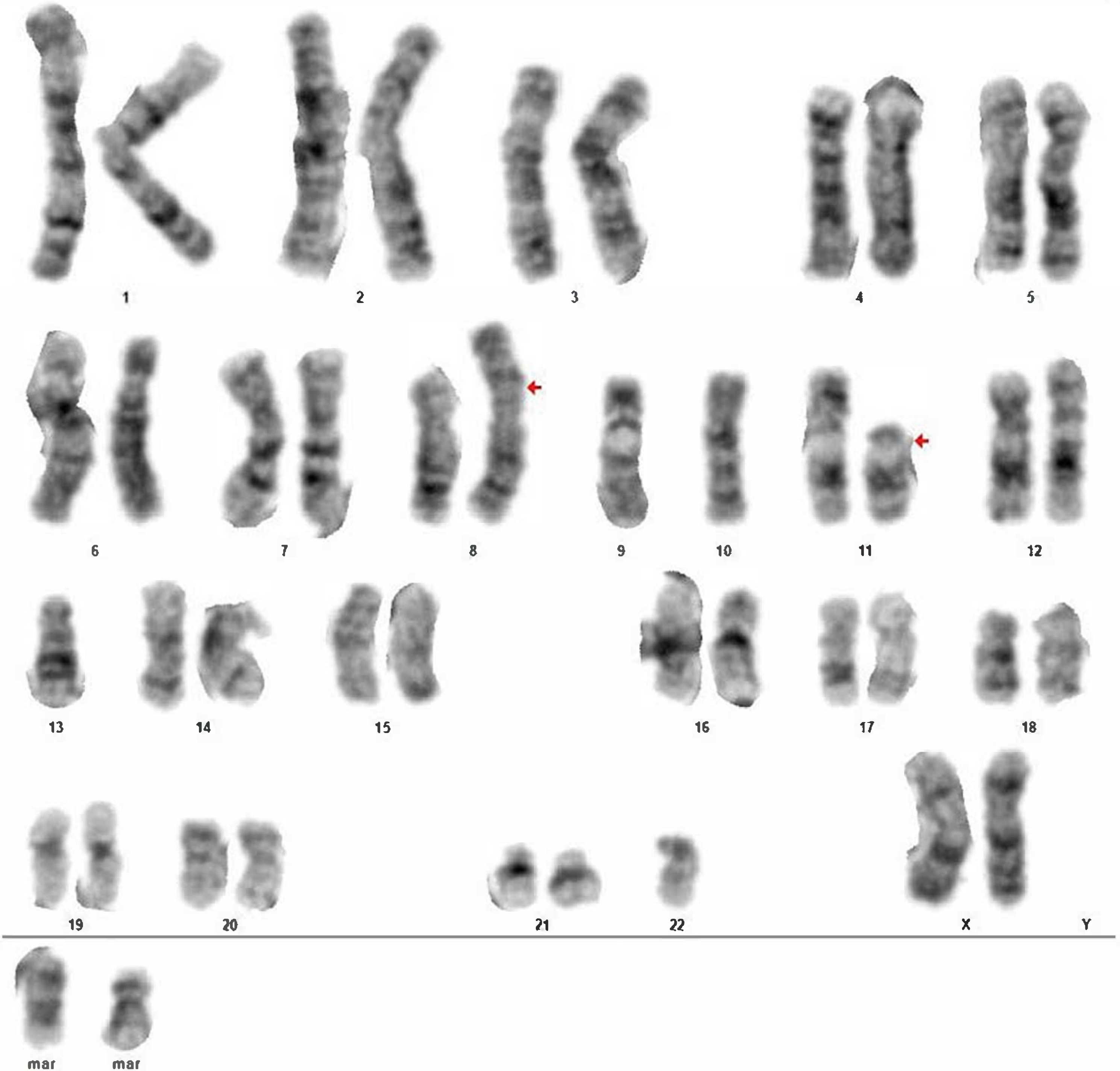

Supplement: Supplementary file 1 [file mmc1.pdf]
