# Supplementary material for: Evaluation of the Immortalized Primary Human Hepatocyte Cell Line Fa2N-4 as a Model for Metabolic Dysfunction–Associated Steatotic Liver Disease
Source: Gastro Hep Adv. 2026 Jul 22;5(11):101069. doi: 10.1016/j.gastha.2026.101069 (PMC13582011; doi:10.1016/j.gastha.2026.101069)

Gastro Hep Advances 2026;5:101069

ORIGINAL RESEARCH—BASIC

Evaluation of the Immortalized Primary Human Hepatocyte Cell Line Fa2N-4 as a Model for Metabolic Dysfunction–Associated Steatotic Liver Disease

Victoria E. J. M. Palasantzas,[1,2] Dicky Struik,[1] Trijnie Bos,[1] Mirjam H. Koster,[1] Jody Gelderloos-Arends,[2] Ellen R. Vos,[2] Krista K. van Dijk-Bos,[2] Sebo Withoff,[2] Jingyuan Fu,[1,2] Joanne A. Hoogerland,[2] and Johan W. Jonker[1]

[1]Department of Pediatrics, University Medical Center Groningen, Groningen, The Netherlands; and [2]Department of Genetics, University Medical Center Groningen, Groningen, The Netherlands

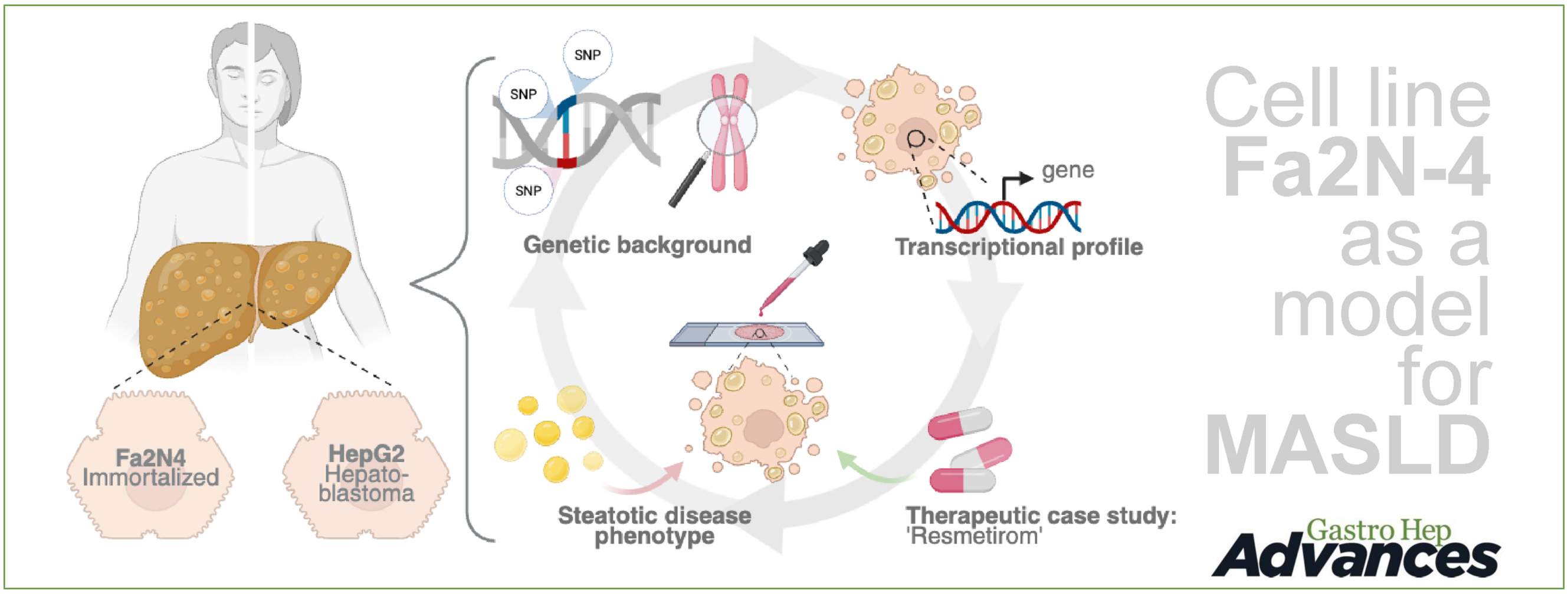

BACKGROUND AND AIMS: Metabolic dysfunction–associated steatotic liver disease (MASLD) is a leading cause of chronic liver disease worldwide. In vitro MASLD studies predominantly rely on hepatocellular carcinoma–derived cell lines such as HepG2 that are poorly differentiated and exhibit cancer-associated metabolic reprogramming that suppresses key adult hepatic functions. Primary human hepatocytes offer greater physiological relevance and capture inter-individual biological variability that reflects human population diversity, but their use is limited by high costs, availability, and rapid dedifferentiation in culture. Here, we characterized the immortalized primary human hepatocyte cell line Fa2N-4 as an alternative in vitro model for MASLD. **METHODS:** We performed a comparative analysis of Fa2N-4 and HepG2 cells, assessing their genomic architecture, lipid-induced steatosis, and pharmacological responsiveness. **RESULTS:** The Fa2N-4 and HepG2 cell models exhibited pronounced differences, including distinct karyotypes, divergent MASLD-associated genetic risk variant profiles, and markedly different transcriptional responses to lipid loading and drug treatment. We further examined the hepatic response to resmetirom, a first-in-class US Food and Drug Administration-approved therapy for treating metabolic dysfunction–associated steatohepatitis. Resmetirom, a thyroid hormone receptor-β agonist, reduced intracellular triglyceride accumulation in Fa2N-4 cells but not HepG2 cells, and this was accompanied by transcriptional changes in mitochondrial glycolysis and oxidative phosphorylation pathways. **CONCLUSION:** These findings demonstrate that hepatocyte

Abbreviations used in this paper: APOE, apolipoprotein E; ATG7, autophagy-related gene 7; ATP6, ATP synthase membrane subunit 6 (mitochondrial gene); CCL5, C-C motif chemokine ligand 5; CCL13, C-C motif chemokine ligand 13; CCL20, C-C motif chemokine ligand 20; CCN2, cellular communication network factor 2; CD44, cell surface glycoprotein CD44; CD73, 5′-nucleotidase ecto; CDCP1, CUB domain-containing protein 1; CIDEC, cell death–inducing DFFA-like effector C; COBLL1, cordon-bleu WH2 repeat protein-like 1; COX1-3, cytochrome c oxidase subunits I–III; CPT1A, carnitine palmitoyltransferase 1A; CREB3L3, cAMP-responsive element–binding protein 3-like 3; CXCL2, C-X-C motif chemokine ligand 2; CXCL8, C-X-C motif chemokine ligand 8; DEG, differentially expressed gene; DGAT2, diacylglycerol O-acyltransferase 2; DHRS3, dehydrogenase/reductase 3; ECH1, enoyl-CoA hydratase 1; ELOVL5, fatty acid elongase 5; ESRRA, estrogen-related receptor alpha; FADS1, fatty acid desaturase 1; FADS2, fatty acid desaturase 2; FDR, false discovery rate; FFAs, free fatty acids; GCKR, glucokinase regulatory protein; GRB14, growth factor receptor-bound protein 14; HSD17B13, hydroxysteroid 17-beta dehydrogenase 13; IFIT2, interferon-induced protein with tetratricopeptide repeats 2; INSIG1, insulin-induced gene 1; LD, lipid droplet; MASLD, metabolic dysfunction–associated steatotic liver disease; MBOAT7, membrane-bound O-acyltransferase domain–containing 7; ND2-6, NADH dehydrogenase subunits 2–6; NT5E, 5′-nucleotidase ecto (CD73); OLR1, oxidized LDL receptor 1; PLIN2, perilipin 2; PNPLA3, patatin-like phospholipase domain–containing 3; PPARα, peroxisome proliferator-activated receptor alpha; RARRES1, retinoic acid receptor responder 1; SAMD9, sterile alpha motif domain-containing protein 9; SCD, stearoyl-CoA desaturase; SIRT5, sirtuin 5; SNP, single nucleotide polymorphism; THRβ, thyroid hormone receptor beta.

Most current article

© 2026 The Author(s). Published by Elsevier Inc. on behalf of American Gastroenterological Association Institute. This is an open access article under the CC BY license (http://creativecommons.org/licenses/by/4.0/).
2772-5723
https://doi.org/10.1016/j.gastha.2026.101069

model selection critically influences experimental outcomes in MASLD research and highlight Fa2N-4 cells as a physiologically relevant platform for mechanistic and translational studies of MASLD therapeutics.

Keywords: MASLD; Hepatic Steatosis; HepG2; Fa2N-4; Hepatic Cell Lines

Introduction

Metabolic dysfunction–associated steatotic liver disease (MASLD) is now the most prevalent chronic liver disease worldwide, affecting approximately one-third of the global population.[1] MASLD encompasses a spectrum of hepatic conditions, ranging from simple hepatic fat accumulation, termed metabolic dysfunction–associated steatotic liver, to more advanced stages, including metabolic dysfunction–associated steatohepatitis, fibrosis, and cirrhosis. In the absence of effective interventions, disease progression may ultimately result in hepatocellular carcinoma and end-stage liver disease.[2] The pathogenesis of MASLD is multifactorial and involves a complex interplay of risk factors, including sedentary lifestyle, obesity, insulin resistance, genetic susceptibility, and alterations in the gut microbiota.[3–5]

First-line management of MASLD includes lifestyle modification for all patients, regardless of disease progression. In recent years, the US Food and Drug Administration and the European Medicines Agency have conditionally approved the pharmacological agents resmetirom and semaglutide for the treatment of metabolic dysfunction–associated steatohepatitis.[6–9] Although resmetirom, a selective thyroid hormone receptor-beta (THRβ) agonist, produces clinical benefit in a subset of patients, response rates are limited to approximately 30% to 40% of treated patients. By contrast, the glucagon-like peptide-1 receptor agonist semaglutide has demonstrated response rates of up to 62%,[10,11] emphasizing the unmet need for therapies with greater efficacy and broader patient applicability. A major bottleneck in the MASLD drug development pipeline remains the limited availability of reliable physiologically relevant preclinical models, both in vivo and in vitro.[12,13]

Liver tumor–derived cell lines, such as HepG2 cells, are the most widely used in vitro models for MASLD, despite their substantial differences from primary hepatocytes. Firstly, transcriptomic comparisons between hepatoma-derived hepatocyte cell lines (HepG2, Huh7, and Hep3B) vs primary hepatocytes have shown a downregulation of pathways regulated by peroxisome proliferator–activated receptor α (PPARα) and reduced expression of lipogenic enzymes such as diacylglycerol acyltransferase 2 (DGAT2) and fatty acid synthase.[14] Secondly, unlike primary hepatocytes, HepG2 and Huh7 cells are not sensitive to (additional) glucose or fructose upon lipid-accumulation stimuli, showcasing a difference in the context of steatosis.[15] Thirdly, genetic variants associated with MASLD susceptibility, including those in the patatin-like phospholipase domain–containing protein 3 gene (*PNPLA3*) and autophagy-related gene 7 (*ATG7*), have been identified in HepG2 cells.[16,17] By contrast, primary human hepatocytes offer greater physiological relevance, but their use as models is limited by cost, availability, donor variability, and rapid dedifferentiation.

To overcome these limitations, immortalized non-tumorigenic hepatocyte cell lines have been developed as alternative in vitro models.[18–20] Among these, Fa2N-4 cells are human primary hepatocyte–derived cells that were conditionally immortalized via transfection with a temperature-sensitive SV40 large T antigen.[21] Alongside their non-tumorous cell origin, they offer several other advantages, including a stable subculture for up to 40 passages[22] and tight control of immortalization with the ability to reverse immortalization.[23] Notably, in Fa2N-4 cells, drug metabolic activity (eg, CYP450 activity), which is typically low in hepatic cancer cell lines, is comparable to that in primary hepatocytes.[19,24] This raises the question of whether immortalized human hepatocytes could also offer benefits for metabolic disease modeling.

Upon stimulation with free fatty acids (FFAs), Fa2N-4 cells recapitulate key features of MASLD, including intracellular lipid accumulation and induction of the inflammatory chemokine CCL5, as demonstrated by Li et al (2017).[25] However, the extent to which this immortalized hepatocyte cell line accurately models hepatic steatosis and responds to therapeutic interventions remains incompletely characterized. In this study, we compare immortalized Fa2N-4 cells with tumor-derived HepG2 cells to delineate their responses to lipid accumulation and MASLD-related pharmacological agents, with the goal of informing the selection of appropriate hepatocyte models for MASLD research. Given their distinct cellular origins, we hypothesize that these models differ in their capacity to recapitulate MASLD-relevant metabolic and inflammatory processes.

Materials and Methods

Cell Culture

HepG2 cells (ATCC HB-8065, Manassas, Virginia, USA) were cultured in Dulbecco's Modified Eagle Medium with GlutaMAX (10569010, Thermo Fisher Scientific, Waltham, Massachusetts, USA) containing 25 mM glucose and supplemented with 10% heat-inactivated fetal bovine serum and 1% (A5256701, Gibco, Thermo Fisher Scientific) and 1% penicillin-streptomycin (15140122, Thermo Fisher Scientific). Fa2N-4 cells[24] were obtained from Tebubio (IFH15, Tebubio, Le Perray-en-Yvelines, France) and cultured on collagen-coated plates (0.02 mg/mL rat tail collagen I [10.9 mg/mL stock, 354249, Corning, New York, USA]) diluted in phosphate-buffered saline (PBS, 14,190–169, Thermo Scientific). Cells were maintained in Williams E medium with GlutaMax (32551087, Gibco, Thermo Fisher Scientific) containing 11 mM glucose and supplemented with 10% heat-inactivated fetal bovine serum, 1% penicillin-streptomycin, 20 mU/mL insulin (100 U/mL injection vial, Sanofi, Paris, France), and 100 nM dexamethasone (20 mg/mL stock, 9265331, Centrafarm, the Netherlands). For experiments, HepG2 and Fa2N-4 cells were seeded in 24-well plates at a density of 1.05×10^5 cells/cm^2 and incubated overnight to

reach 60–70% confluence prior to stimulation. Cell line authentication was confirmed by short tandem repeat profiling (Eurofins Genomics Europe Food/Environment/White Biotech Products and Services GmbH, Eurofins, Luxembourg City, Luxembourg). Cells were tested negative for mycoplasma contamination by polymerase chain reaction.

Induction of MASLD

To mimic hepatic steatosis in vitro, HepG2 and Fa2N-4 cells (up to passage 22 and 8, respectively) were stimulated with fructose (D-(−)-Fructose, F0127, Sigma, Merck KGaA) and a 1:2 ratio of palmitic acid:oleic acid (03880 and P9767, Sigma-Aldrich, Merck KGaA). A FFA-bovine serum albumin (BSA) complex was prepared by dissolving 10 mM palmitic acid or oleic acid (dissolved in 100% ethanol, dried under gaseous N_2) in 10% BSA, A6003, Sigma, Merck KGaA). The solution was adjusted to pH 7.4. An equal volume of BSA was used as a control. Cells were stimulated with 1 mM fructose and 600 μM FFA-BSA complex for 48 hours, unless otherwise specified. Resmetirom (MGL-3196, catalog #NC1772005, Selleckchem, Houston, Texas, USA) dissolved in dimethyl sulfoxide (DMSO) was added 24 hours after induction of steatosis at 200 μM, unless otherwise specified. D-(+)-Glucose (catalog G8644–100 ML, Sigma, Merck KGaA) and low-glucose-containing Dulbecco's Modified Eagle Medium (11885084, Gibco, Thermo Fisher Scientific) were used to modulate glucose concentration in specified experiments.

Cell Viability Analysis

Cell viability following resmetirom treatment (5–500 μM) was assessed using the 3-(4,5-dimethylthiazol-2-yl)-2,5-diphenyltetrazolium bromide assay. After treatment, media were replaced with fresh media containing 3-(4,5-dimethylthiazol-2-yl)-2,5-diphenyltetrazolium bromide (3-[4,5-dimethylthiazol-2-yl]-2,5-diphenyltetrazolium Bromide) (Invitrogen, Thermo Fisher, catalog #M-2128 and #V13154) and incubated for 4 hours. Formazan was dissolved in DMSO, and absorbance was measured at 540 nm using a Synergy H4 Hybrid Microplate Reader (BioTek, Winooski, VT, USA). Culture media without cells served as background, untreated cells as a negative control, and cells treated with 15% DMSO as a positive control (100% non-viable). Viability was expressed relative to vehicle (1% DMSO). Data were analyzed using GraphPad Prism 10.4.1 (San Diego, CA, USA).

Karyotyping

Cells were fixed in a 3:1 methanol:acetic acid solution and dropped onto microscope slides to prepare chromosome spreads. GTW-banding was performed using pancreatin and Giemsa staining (Merck KGaA) to visualize G-bands in metaphase nuclei. Metaphase images were captured with Applied Spectral Imaging. Karyotypes were analyzed according to the International System of Human Cytogenomic Nomenclature 2020.

Genetic Single Nucleotide Polymorphism Array (GSA)

DNA isolated from both cell lines was used in the Illumina HTS array with the GSAMD-24v3-0-EA research chip. Variant calling by Opticall software and data extracted from the VCF files are publically available here: https://zenodo.org/records/21904147 Supplementary File 4.

Intracellular Triglyceride Analysis

Cells were washed with PBS and collected in 1x TBS to quantify intracellular triglycerides. Cell lysates were prepared by sonication (Sonics Vibra-Cell VCX-500) for 10 seconds at 30% amplitude, and the protein concentration was determined using the bicinchoninic acid protein assay (Pierce, Thermo Fisher Scientific, catalog 23227).[26] Samples were normalized to the lowest protein concentration prior to lipid extraction. Lipids were extracted using the Bligh and Dyer method,[27] employing chloroform/methanol (2:1, v/v), followed by vortexing and centrifugation. The lipid-containing phase was dissolved in 2% Triton X-100 in chloroform, vortexed, and dried under a nitrogen stream. Dried lipids were resuspended in deionized water and incubated for 1 hour at 37 °C. Triglyceride concentrations were quantified using an enzymatic colorimetric assay (Triglycerides FS; 157109910917, Diasys Diagnostic Systems GmbH, Holzheim, Germany) and measured on a Synergy H4 Hybrid Microplate Reader (BioTek Instruments Inc). A glycerol standard curve (0.82–105 μM/mL) was generated using Precimat Glycerol (10166588, Roche Diagnostics GmbH, Mannheim, Germany).

Oil Red O Staining

Cells were cultured on coverslips coated with 1% collagen. After lipid loading, the cells were washed 3 times with PBS, fixed with 4% paraformaldehyde for 10 minutes, and washed 3 times with PBS. Oil Red O (O0625, Sigma, Merck KGaA) reagent was dissolved in isopropanol to prepare a 60% working solution in Milli-Q water. For neutral lipid staining, coverslips were rinsed with deionized water for 2 minutes and washed twice in 60% isopropanol. Coverslips were then incubated with Oil Red O solution for 10 minutes. Next, the coverslips were washed with 60% isopropanol, rinsed with tap water, stained with hematoxylin (1.04302, Merck KGaA) for 1.5 minutes, and rinsed 3 times in tap water. Coverslips were fixed to microscope slides with Aquatax (1.08562, Sigma, Merck KGaA, Darmstadt, Germany) and visualized using a digital slide scanner (NanoZoomer S360, C13220-04, Hamamatsu Photonics, Shizuoka, Japan). Representative images were taken using NDPView2 (U12388-01, Hamamatsu Photonics).

Quantification of Oil Red O Staining

Oil Red O staining was quantified using QuPath[28] (v0.51). Whole-slide images were loaded as brightfield H&E stainings. Lipid droplets (LDs) were detected using the pretrained StarDist[29] module for RGB images (he_heavy_augment.pb), supplemented with a custom script for LD identification (Supplementary File 1). Oil Red O signal was isolated via the eosin channel, using a prediction threshold of 0.4 and a pixel size of 0.25. Detected objects were measured via QuPath's detection measurement tools and subsequently filtered to exclude misidentified structures based on the following criteria: detection probability ≥0.75, circularity ≥0.93, and median intensity ≥0.2. Identical quantification areas were defined using QuPath's grid function, applying perimeters of

1000 μm across all slides and samples. LD size was expressed as surface area (μm^2). The average number of LDs per perimeter was calculated across biological (n $\geq$ 2) and technical (n $\geq$ 3) replicates. Statistical analyses and data visualization were performed using GraphPad Prism (v10.4.1 [532]), with results presented as median and interquartile range.

RNA Isolation

The RNeasy mini kit (74106, Qiagen, Aarhus, Denmark) was used to lyse the cells and isolate the RNA according to the manufacturer's protocol. Samples prepared for RNA sequencing (RNA-seq) were treated with RNase-free DNase kit (79254, Qiagen).

RNA Sequencing

RNA quality and quantity were assessed using an Agilent 5400, and all samples had RNA integrity scores above 8. Sequencing was performed at Novogene (Cambridge, UK), which performed non-strand-specific mRNA library preparation with poly(A) enrichment. Sequencing reads were aligned to the genome reference ID "ensembl_109_homo_sapiens_grch38_primary" using HISAT2 (v2.0.5). To quantify gene expression levels, featureCounts (v1.5.0-p3) was used. Downstream data analysis was performed in R (v4.3.1). DESeq2 was used to analyze differentially expressed genes (DEGs) (utilizing the Wald test) in the conditions vs controls.[30] The resulting *P* values were adjusted using the Benjamini-Hochberg method to control the false discovery rate (FDR). A *P* value $\leq$.05 threshold was set for significant differential gene expression. To generate plot, we used ggplot2_4.0.0 as well as org.Hs.eg.db_3.22.0 and AnnotationDbi_1.72.0 for gene annotations. Follow-up gene enrichment analysis was performed using the Gene Ontology Term and MSigDB Hallmarks datasets in iPathwayGuide (AdvaitaBio Corporation, https://ipathwayguide.advaitabio.com/)[31,32] and Clusterprofiler[33] for the network analysis. To visualize the .csv output from iPathwayGuide, we used ggplot2_4.0.0 in R to create dot plots. Additional visual enhancements in R were applied using scales_1.4.0, tidyselect_1.2.1, and RColorBrewer_1.1–3.

Statistical Analysis

Statistical analyses were performed using GraphPad Prism 10.4.1. Differences between groups were assessed using the unpaired Mann-Whitney *U* test or Kruskal-Wallis test, unless stated otherwise. Data are presented as the median of $\geq$3 independent replicates, except for karyotyping and GSA. A *P* value <.05 was considered statistically significant.

Results

Genetic and Karyotypic Characterization of Fa2N-4 and HepG2 Hepatocyte Models

Tumor-derived cell lines generally contain various genetic and chromosomal abnormalities accumulated during tumorigenesis, while SV40-immortalized lines are suggested to have greater genomic stability.[34–36] Because genetic factors account for approximately 25–50% of the interindividual susceptibility to MASLD,[37–39] we first characterized and compared the genetic background of the immortalized Fa2N-4 and tumor-derived HepG2 hepatocytes. To this end, we performed karyotypic analysis for chromosomal abnormalities and assessed the presence of MASLD-associated risk variants (Figure 1A and B).

Consistent with previous reports,[40] HepG2 cells exhibited extensive chromosomal abnormalities, including a translocation between chromosomes 1 and 21; structural additions on chromosomes 16 and 17 alongside trisomy of chromosome 2, 16, and 17; tetrasomy of chromosome 20; and a recurrent marker chromosome (Figure 1A and C). In contrast, the karyotype of Fa2N-4 cells, which had not previously been described, revealed a distinct aneuploidy pattern characterized by monosomy of chromosomes 9, 10, 13, 20, and 22 (Figure 1B). Two marker chromosomes were also observed, suggesting uncharacterized chromosomal rearrangements or partial chromosome losses (Figure 1C). HepG2 cells are male-derived (XY), whereas Fa2N-4 cells are female-derived (XX). Apart from chromosome 20, which is tetrasomic in HepG2 and monosomic in Fa2N-4 cells, the 2 cell lines shared minimal overlap in chromosomal abnormalities, confirming their fundamentally different genomic architectures.

We next assessed the MASLD-associated genetic variants in both cell lines. A total of 23 MASLD-related single nucleotide polymorphism (SNP) variants were curated from the literature (Supplementary Table 1).[41–45] These include both coding and non-coding regulatory variants. These were identified using GSA (original data available: https://zenodo.org/records/21904147) processing the GSA to VCF, all 23 SNP positions were called in Fa2N-4 cells. In HepG2 cells, 22 variants were called, but the *GCKR* missense variant rs1260326 could not be reliably called due to low genotype quality. Overall, the 2 cell lines exhibited limited overlap in MASLD-associated SNPs (Figure 2A). Fa2N-4 cells carried a higher number of risk alleles (total count 12 risk alleles) than HepG2 cells (6 risk alleles) (Supplementary Table 1). Functional annotation further revealed that the risk alleles present in HepG2 cells were predominantly associated with mitochondrial function and autophagy. In contrast, variants identified in Fa2N-4 cells were more strongly linked to lipid metabolism pathways (Figure 2B). Most of the variants found in both cell types were common (defined as $\geq$ 0.01),[46,47] with most alternative allele frequencies >0.2, based on the allele frequencies reported in the 1000 Genomes Project data.[48] Each cell line also harbored one rare variant (alternative allele frequency <0.01)—rs36117895 in *ATG7*[49] in HepG2 cells and rs7412 in *APOE* in Fa2N-4 cells.[50] Other notable risk variants in HepG2 include rs12216101 associated with *SIRT5* and in Fa2N-4, rs6834314 in *HSD17B13*, as well as multiple *MBOAT7*-TMC4 variants (rs8736, rs626283, rs641738).

Several MASLD-associated SNPs were located within chromosomally abnormal regions (Figure 2C). For instance, the HepG2 trisomy of chromosome 2 carries rs13389219 (*COBLL1-GRB14*) and rs780094 (*GCKR*), potentially influencing downstream gene expression. The other SNP loci were not previously associated with chromosomal abnormalities determined by karyotype. To assess potential

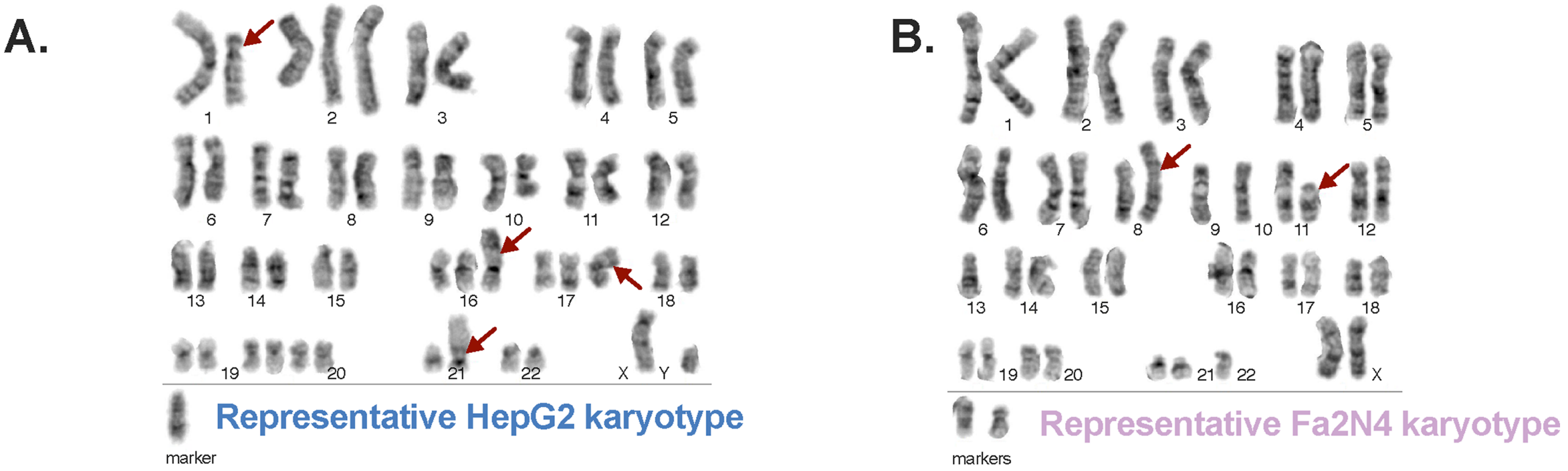

C.

Karyotype	HepG2	Fa2N4
ISCN results	51~52,XY,t(1;21)(p22;p11),+2,+16,add(16)(p13),+17,?add(17)(p11.2),+20,+20,+mar[cp10]	Line I: 43,X,-X,add(8)(p2?1),der(9;11)(q10;q10),-10,-13,-22,+2mar[cp3]<br>Line II: 41~45,X,-X,add(8)(p2?1),-9,-10,del(11)(p11.2),-13,+17,-22,-22,+2mar[cp7]
Number of chromosomes	51-52	41-45
Identified sex chromosomes	XY	XX
Abnormalities involving translocations, additions or deletions	Translocation from chromosome 1 → 21: p22 to p11<br>Addition on chromosome 16: p13<br>Addition on chromosome 17: p11.2	Addition on chromosome 8: p2-1<br>Deletion on chromosome 9<br>Deletion on chromosome 11: p11.2
Numerical gain or loss	Trisomy of chromosome 2 (+1), 16 (+1) and 17 (+1)<br>Tetrasomy of chromosome 20 (+2)	Monosomy of chromosome 9 (-1), 10 (-1), 13 (-1), 20 (-1) and 22 (-1)
Marker chromosomes	1 composite karyotype in at least 10 cells [cp10]	2 different composite karyotypes in at least 7 cells [cp7] of which one resembles chromosome 20

Figure 1. Representative karyogram of HepG2 and Fa2N-4. (A, B) Karyogram of (A) HepG2 and (B) Fa2N-4 cells, with translocations, additions, and deletions indicated with a red arrow. Marker chromosomes are shown: one unidentified marker 'mar' in the HepG2 cells (cp10) and two unidentified markers ('mar') in the Fa2N-4 cells (cp7). (C) Overview of identified abnormalities. Karyograms have been edited for clarity (enlargement of arrows and numbers). The original karyograms can be found in Supplementary File 3.

transcriptional changes based on the presence of the identified SNPs on their loci, we determined the expression levels of SNP-related genes using RNA-seq (Figure 2D). Transcripts corresponding to all MASLD-associated genes were detected in both cell lines. *COLBLL1/GRB14, GCKR, SIRT5, PNPLA3*, and *APOE* expression was significantly higher in HepG2 cells compared to Fa2N-4 cells, whereas *ATG7* and *MBOAT7* expression was significantly lower.

Together, these data demonstrate that HepG2 and Fa2N-4 cells harbor distinct chromosomal abnormalities and distinct MASLD-associated genetic risk profiles. Although the expression of SNP-associated genes was only slightly affected, we cannot exclude that this might be due to genetic differences. We suspect these genomic differences may differentially influence hepatocyte biology in steatotic liver disease models.

Differential Lipid Accumulation and Transcriptomic Remodeling in Fa2N-4 and HepG2 Hepatocyte Models

Given the distinct genetic profiles of Fa2N-4 and HepG2 cells, we examined whether these cell lines respond differently to steatosis induction. Under basal culture conditions, HepG2 cells already contained low but detectable levels of neutral lipids (Figure 3A and B), whereas Fa2N-4 cells showed negligible lipid accumulation (Figure 3C and D). Upon stimulation with FFA and fructose, intracellular triglyceride levels increased significantly in both cell lines. In HepG2 cells, triglycerides rose from a median of 60–286 nmol/mg protein ($P < .001$), accompanied by prominent neutral LDs, vacuolization, and a relatively small cytoplasmic volume (Figure 3B). In contrast, Fa2N-4 cells, which are characterized by a larger cytoplasm, showed an increase from a median of 0–138 nmol/mg protein following lipid loading (Figure 3D). The difference in intracellular triglyceride levels between the cell lines may be partly attributed to the substantial difference in glucose concentrations in their basal culture medium (25 mM in HepG2 vs 11 mM in Fa2N-4). Therefore, we assessed if intracellular triglyceride levels are affected by basal medium glucose concentration. Under unstimulated conditions, we observed no difference in intracellular triglyceride levels in both cell lines. Under steatotic conditions, however, increasing glucose from 11 mM to 25 mM

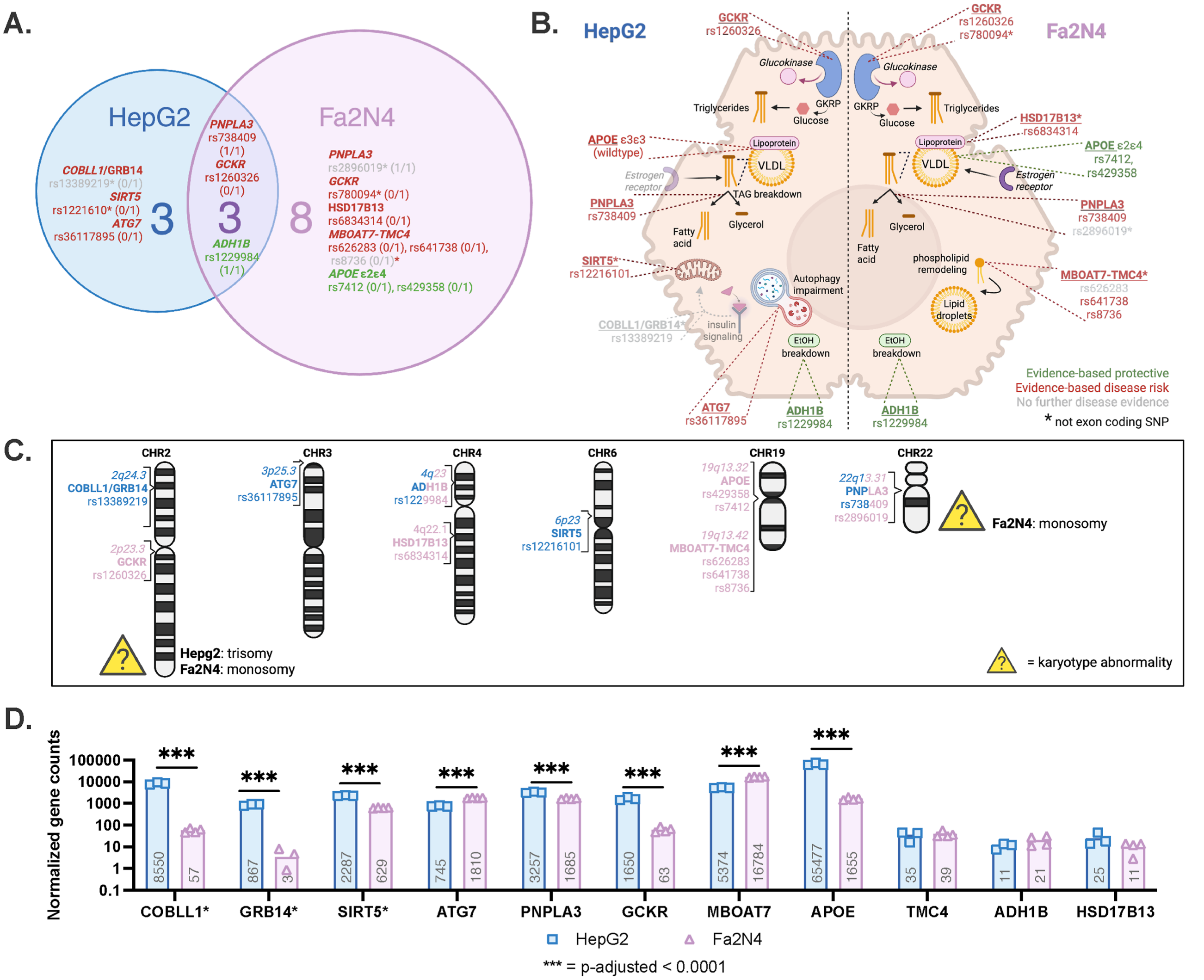

Figure 2. Causal SNPs for metabolic dysfunction–associated steatotic liver disease (MASLD) present in HepG2 and Fa2N-4 cells. (A) Venn diagram showing overlapping and unique single nucleotide polymorphisms (SNPs) per cell line. (B) Graphical abstract representing the function of associated genes and the potential hepatic intracellular effect with non-coding SNP variants (*), no further functional validation from genome-wide association study identifications (gray), and increased (red) or decreased (green) risk variant for MASLD. (C) Chromosome location of the identified SNPs and associated genes. (D) Normalized gene counts of the genes associated with the identified SNPs. This figure was created in BioRender. Palasantzas, V. (2026) (https://BioRender.com/6czejv3).

further elevated triglyceride accumulation in Fa2N-4 cells but not HepG2 cells (Supplementary Figure 1). We next assessed whether LD characteristics differed between cell lines, with LD size, area, and staining intensity quantified using the StarDist deep-learning module in QuPath[29] (Supplementary Figure 2). Although not statistically significant, HepG2 cells did show a trend toward larger LDs, with 81% of droplets exceeding 5 μm^2 compared to 66% in Fa2N-4 cells (Figure 3E and F).

To investigate whether transcriptional adaptations underlie differences in the steatosis phenotype, we performed transcriptomic analysis following steatosis induction. In HepG2 cells, most DEGs were mitochondrial-encoded components of the oxidative phosphorylation machinery, including *ND2–ND6*, *COX1–3*, and *ATP6* (Figure 3G). Pathway enrichment analysis revealed downregulation of mitochondrial ATP synthesis, electron transport chain activity, and cellular oxygen homeostasis (Figure 3I). These pathways were driven by a largely overlapping gene set (including *ND2-6*, *COX1-3*, and *ATP6*) in steatotic HepG2 cells, suggesting a reduction in mitochondrial respiratory capacity (Supplementary File 2, Supplementary Table 1). In contrast, Fa2N-4 cells exhibited increased expression of genes involved in lipid metabolism and catabolism (Figure 3H). Lipid loading induced upregulation of fatty acid β-oxidation genes (*CPT1A*, *ECH1*) and the LD-associated protein *PLIN2*, which stabilizes LDs and limits autophagy-mediated degradation.[51] In line, some lipophagy-related genes (*RAB7A, RAB10*, and *RAB18*) were expressed at higher levels at baseline in Fa2N-4 cells

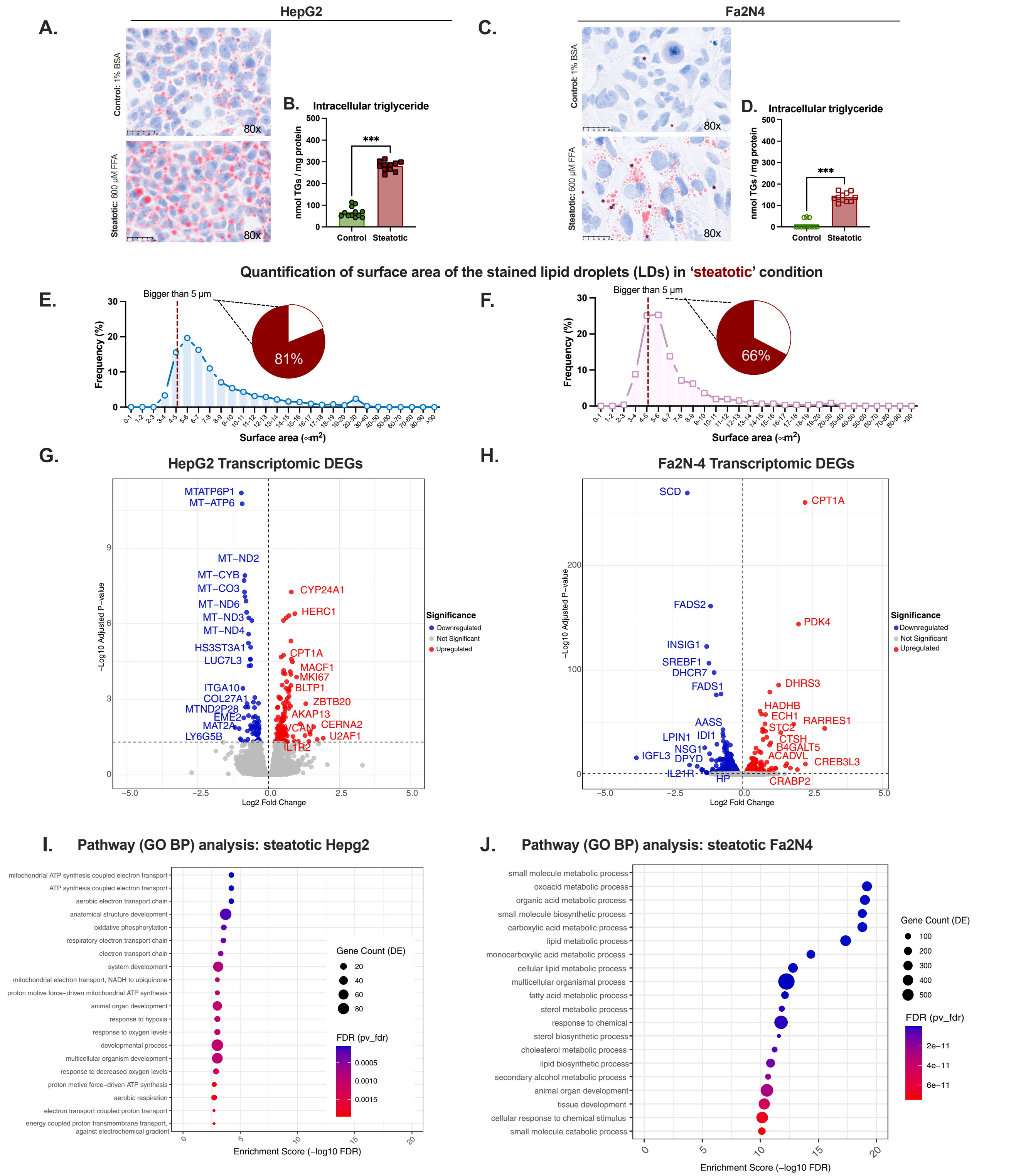

Figure 3. Steatotic morphology and transcriptional changes upon free fatty acid (FFA) and fructose stimulation. (A and B) Neutral lipid staining for (A) HepG2 cells stimulated with BSA (control) or 600 μM FFA and 1 mM fructose and (B) intracellular triglyceride quantification. (C and D) Neutral lipid staining for (C) Fa2N-4 cells stimulated with BSA (control) or 600 μM FFA and 1 mM fructose and (D) intracellular triglyceride quantification. (E and F) Lipid droplet (LD) size in (E) steatotic HepG2 cells and (F) steatotic Fa2N-4 cells. (G and H) Volcano plots of differentially expressed genes (DEGs; FDR $\leq$0.05) in (G) steatotic HepG2 cells and in (H) Fa2N-4 cells (compared to control). (I and J) Topology-based pathway analysis with the Gene Ontology Biological Processes database on DEGs identified in (I) steatotic HepG2 cells and (J) Fa2N4 cells (compared to control).

compared to HepG2 cells, although other lipid-droplet genes (*PLIN2, PNPLA2, LIPA*, and *ATG13*) had higher basal expression in HepG2 cells (Supplementary File 2, Supplementary Table 5). Concurrently, lipogenic regulators (*SREBF1*, *INSIG1*) and downstream enzymes (*SCD, FADS2*) were downregulated. Enrichment analysis highlighted metabolic pathways related to fatty acids, cholesterol, and sterols, including oxoacid and monocarboxylic acid metabolism (Figure 3J and Supplementary File 2, Supplementary Table 2). These pathways are central to the Krebs cycle and β-oxidation and may indicate that Fa2N-4 cells redirect excess lipids toward energy production via acetyl-CoA generation upstream of oxidative phosphorylation. Thus, despite a shared phenotype of intracellular lipid accumulation, HepG2 and Fa2N-4 cells exhibit fundamentally distinct transcriptional responses to lipid overload.

Resmetirom Selectively Reduces Intracellular Lipid Accumulation in Fa2N-4 Cells

Given the distinct lipid accumulation and transcriptional response to lipid loading we observed in Fa2N-4 and HepG2 cells, we next examined whether these models also differ in their response to resmetirom (MGL-1396), a THRβ agonist approved for the treatment of MASLD with progressive steatohepatitis and/or fibrosis.[52] Resmetirom has been reported to act primarily on mitochondrial pathways, including β-oxidation, mitophagy, mitochondrial biogenesis, and oxidative phosphorylation.[53] We first assessed resmetirom toxicity and found that cell viability remained above 60% in both cell lines (Supplementary Figure 3) at concentrations up to 200 μM, consistent with previous studies using HepG2 cells.[54] Based on these results, we treated steatotic Fa2N-4 and HepG2 cells with 200 μM resmetirom for 24 h in subsequent experiments. Resmetirom reduced positive lipid staining only in Fa2N-4 cells (Figure 4A and B), while LD size was not affected (Figure 4C and D). Intracellular triglyceride quantification confirmed a significant 37% reduction in Fa2N-4 cells ($P <$ .001), but only a modest 9% reduction in HepG2 cells (Figure 4E and F).

To gain insight into potential mechanisms underlying the lipid-lowering effect of resmetirom in Fa2N-4 cells, we performed transcriptome analysis. Both cell lines express THRβ, with the Fa2N-4 cells displaying an average normalized gene count of 512 and HepG2 cells an average normalized gene count of 1468 (Supplementary File, Supplementary Table 4). Treatment with resmetirom did not significantly change the expression of THRβ in either cell line (neither *P* value nor *P*-adjusted). Notably, classical lipid metabolism genes were not among the top DEGs, with the exception of *RARRES1*, which has been linked to impaired fatty acid metabolism[55] (Figure 4G). Several DEGs, including *RARRES1* and *DHRS3*, were associated with retinoid X receptor signaling, the heterodimeric partner of THRβ. Additional transcriptional changes included upregulation of *NT5E* (*CD73*), which is associated with anti-inflammatory and immunosuppressive functions, and downregulation of *CCN2*, indicative of anti-fibrotic activity.[56,57] Genes promoting liver regeneration, such as *CDCP1*[58] and *CD44*,[59] were also upregulated. Top upregulated genes in response to steatosis induction included CPT1α and PLIN2, and these remain upregulated after resmetirom treatment but are less affected in terms of fold change and significant outcome (Supplementary File 2, Supplementary Table 4). Unbiased pathway network analysis using Gene Ontology Biological Processes further revealed significant enrichment of glucose and glycogen metabolic pathways as a major cluster in the network of enriched pathways (Figure 4H).

Hallmark pathway analysis restricted to upregulated DEGs indicated activation of glycolysis, oxidative phosphorylation, and reactive oxygen species (Figure 4I). Rather than a uniform upregulation of the entire pathway, this enrichment in the glycolysis pathway was driven by a subset of genes, including rate-limiting enzymes such as *HK, PFKP/PFKFB4*, and *GAPDH* and associated metabolic regulators (Supplementary File 2, Supplementary Table 6). It should be noted that the glycolytic pathway contains several enzymes that also mediate reactions in the opposing gluconeogenic pathway. This metabolic shift in transcription of genes involved in glucose homeostasis may be mediated by upregulation of cAMP responsive element–binding protein 3 Like 3 (CREB3L3). CREB3L3 is a liver-specific endoplasmic reticulum-anchored transcription factor activated downstream of the estrogen-related receptor-γ (ESRRA), which was also upregulated. Consistent with reported CREB3L3-dependent metabolic remodeling,[60] we observed changes in expression of downstream targets, including an increase in the lipoprotein receptor *SCARB1*, downward trends in lipid storage-associated genes (*CIDEC*, FDR = 0.068) and fatty acid desaturase (*FADS1*, FDR = 0.052), and significant downregulation of the fatty acid elongation enzyme-encoding genes *ELOVL2* and *ELOVL5*. In contrast, the majority of downregulated genes clustered within immune and inflammatory signaling pathways, particularly TNFα and IFNα/γ signaling (Figure 4J). TNFα-associated genes suppressed by resmetirom included *OLR1*, a pro-inflammatory macrophage mediator[61]; *CXCL2*, a macrophage recruitment chemokine; and *IL7R*, an activator of the JAK/STAT pathway towards hepatocellular carcinoma.[62] IFNα/γ-related downregulated genes included antiviral effectors such as *IFIT1-3, IFI44*, and *OAS2*. These findings suggest that resmetirom induces an anti-inflammatory transcriptional signature at least in part by suppressing interferon-stimulated genes, which may additionally influence lipid handling via modulation of lipophagy and cholesterol metabolism.[63–65]

Collectively, these data provide insights into the difference in response to resmetirom between the Fa2N-4 cells and the HepG2 cells. Altogether, this study highlights the importance of cellular origin and metabolic competence in therapeutic response.

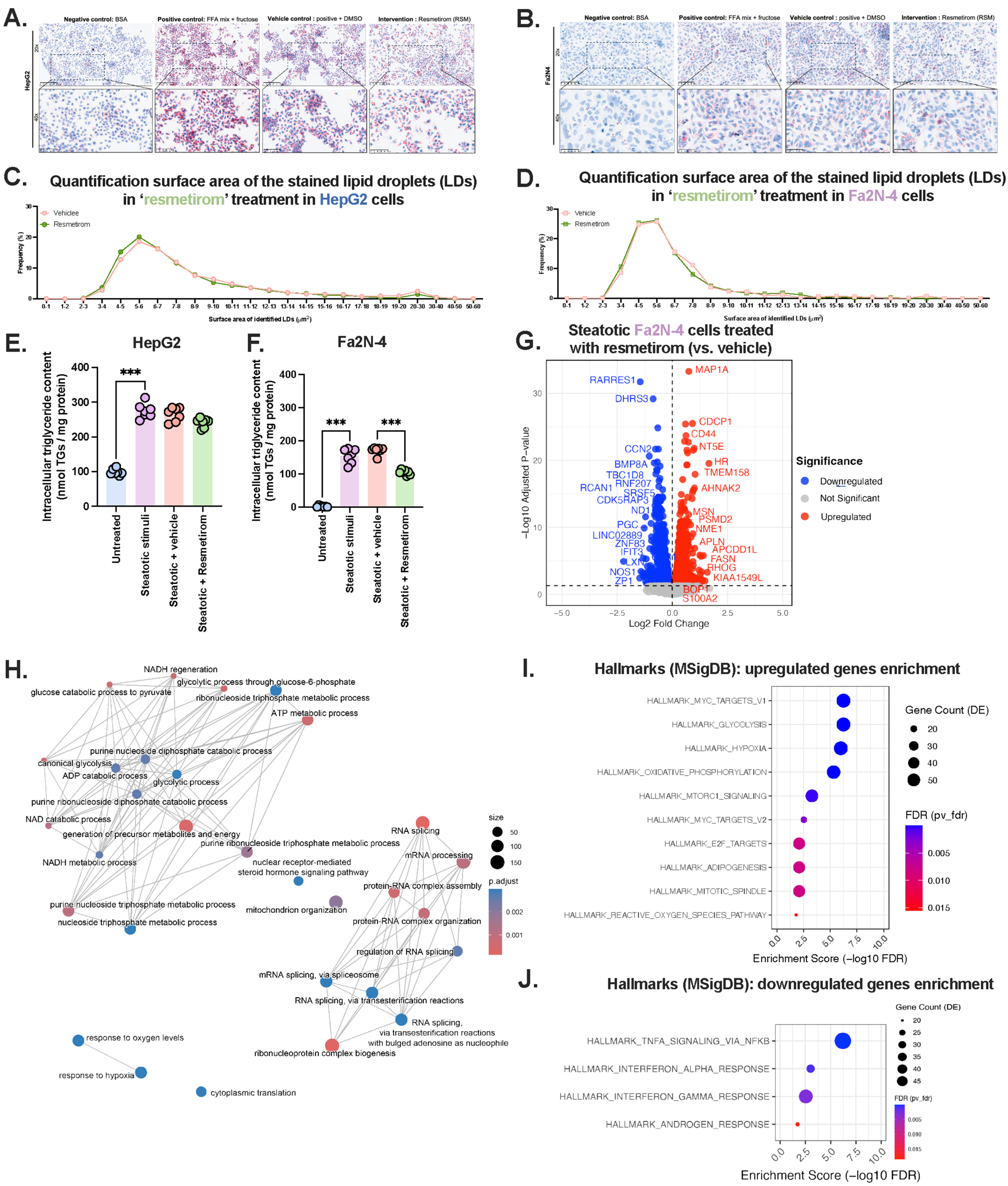

Figure 4. Anti-steatotic effect of resmetirom in HepG2 and Fa2N-4 hepatocyte cell lines and the glycolytic adaptation induced in responsive steatotic Fa2N-4 cells. (A and B) Neutral lipid stainings from stimulated (A) HepG2 cells or (B) Fa2N-4 cells at 20x and 40x magnification. Cells were stimulated for 48 hours with BSA (negative control), steatotic stimuli (positive control), or 24 hours steatotic stimuli with either vehicle or 200 μM resmetirom for an additional 24 hours. (C and D) Surface area of lipid droplets (LDs) quantified from scanned images of steatotic + vehicle-treated and steatotic + resmetirom-treated (C) HepG2 cells and (D) Fa2N-4 cells. (E and F) Intracellular triglycerides from (E) HepG2 cell lysates or (F) Fa2N-4 cell lysates after 48 -hour stimulations. (G) Volcano plot of differentially expressed genes (DEGs; FDR $\leq$0.05) of Fa2N-4 cells treated with steatotic stimuli and subsequently with vehicle or resmetirom. (H) Network analysis of Gene Ontology Biological Processes pathway analysis of all DEGs. (I and J) Significantly (I) upregulated and (J) downregulated DEGs based on the MSigDB database for Hallmarks.

Discussion

In this study, we systematically evaluated the immortalized primary human hepatocyte cell line Fa2N-4 as a model for MASLD. The Fa2N-4 cell line was compared to HepG2, the most commonly used tumor-derived hepatocyte cell line, with respect to genetic background, steatotic phenotype, transcriptional adaptations, and response to pharmacological intervention. Our findings demonstrate substantial differences between these models that have important implications for studying MASLD.

At the genomic level, the 2 cell lines displayed distinct chromosomal abnormalities. HepG2 cells exhibited tri- or tetrasomies, whereas Fa2N-4 cells showed monosomies, reflecting fundamentally different degrees of chromosomal stability. We also report the genotypes of 22 additional SNPs associated with MASLD. Both cell lines carried established MASLD-associated risk variants in *PNPLA3* (rs738409)[66] and *GCKR*, but each also harbored distinct additional variants. HepG2 cells contained 2 recently identified risk variants, in *SIRT5* (rs12216101)[67] and *ATG7* (rs36117895),[16] whereas Fa2N-4 cells carried a higher cumulative number of MASLD-associated risk alleles, including variants in *HSD17B13* (rs6834314)[68] and *MBOAT7-TMC4* (rs8736, rs626283, rs641738).[69,70]

Interpretation of genetic risk variants must be considered in the context of aneuploidy. On the one hand, genes such as *COBLL1-GRB14* and *GCKR* are located on chromosome 2, which is trisomic in HepG2 cells but monosomic in Fa2N-4 cells. Consistent with this, we observed increased expression of these genes in HepG2 cells and decreased expression in Fa2N-4 cells. On the other hand, high-throughput SNP array data alone cannot determine whether the detected risk alleles reside on the retained chromosome copy. Targeted validation (eg, Sanger sequencing) will be required to confirm allelic distribution and functional relevance. Such future investigation may also reveal an interaction between aneuploidy and SNP burden. The literature shows that HepG2 cells exhibit greater phenotypic variability within and across studies.[40,71–73] Furthermore, we hypothesize that Fa2N-4 cells, which carry more single chromosome copies, may exhibit a more consistent genotype–phenotype relationship. These findings are in line with studies on other human immortalized cell lines that report a more stable genotype.[34–36] Additionally, Fa2N-4 cells harbor multiple MASLD-relevant SNPs in *PNPLA3*, *GCKR*, and *MBOAT7-TMC4*, and the reduction in *PNPLA3* gene expression we observed mirrors findings in MASLD patients homozygous for the risk allele.[74] We hypothesize that these SNPs affect the development of steatosis. Importantly, several variants (eg, *GCKR* rs780094 and *MBOAT7* rs641738[75]) confer risk primarily in the homozygous state, underscoring the need for careful genotype interpretation.

Functionally, both cell lines accumulated lipids in response to fructose and FFA stimulation, but their basal and induced phenotypes differed. Under unstimulated conditions, HepG2 cells exhibited higher intracellular triglyceride levels compared to Fa2N-4 cells. Upon steatotic stimulation, LD size did not differ significantly between the models, yet absolute triglyceride levels remained distinct. More strikingly, the transcriptional responses to lipid loading diverged substantially. Prolonged exposure to fructose and FFAs led to downregulation of mitochondrial oxidative phosphorylation pathways in HepG2 cells, suggesting impaired mitochondrial function. In contrast, Fa2N-4 cells displayed adaptive transcriptional responses characterized by transcriptional upregulation of lipogenic and lipolytic pathways, including sterol and cholesterol biosynthesis. This transcriptional profile of the Fa2N-4 cells closely resembles the genes observed to be involved in MASLD progression, as reported in SteatoSITE bulk RNA-seq data,[76] MASL-specific hepatocytes,[77] and steatotic primary human hepatocytes stimulated with fatty acids.[78] The relatively modest transcriptional response we observed in steatotic HepG2 cells contrasts with several published studies.[79–81] However, this discrepancy is consistent with known variability in HepG2 transcriptomic datasets, which has been attributed to substantial inter-laboratory differences in culture conditions, including media composition and glucose concentrations.[14,73] By contrast, Fa2N-4 cells demonstrated a robust, disease-relevant transcriptional response to lipid loading, characterized by enrichment of genes involved in fatty acid and sterol metabolism, supporting their suitability as a transcriptionally responsive MASLD model.

These transcriptional differences also provide a potential mechanistic explanation for the divergent responses to resmetirom. Resmetirom reduced intracellular triglyceride levels in Fa2N-4 cells but not HepG2 cells, which may be caused by impaired mitochondrial function.[82] As a THRβ agonist, resmetirom reportedly enhances mitochondrial β-oxidation and lipid clearance.[83] In Fa2N-4 cells, resmetirom induced expression of CPT1α, suggesting increased mitochondrial β-oxidation, alongside changes in LD-associated genes, including upregulation of *PLIN2* and downregulation of *PLIN5*. Additionally, transcriptional repression of the chemokines *CXCL2*, *CXCL8*, *CCL13*, and *CCL20* and of other inflammatory mediators, such as *OLR1*, *IFIT2*, and *SAMD9*, indicated suppression of inflammatory gene expression.[61] These findings align with previous reports demonstrating the anti-steatotic and LD-modulating effects of resmetirom in hepatic organoids.[84] We did not observe a reduction in average LD surface area in Fa2N-4 cells, which may reflect methodological differences between immunofluorescence and Oil Red O staining, with the latter less sensitive for absolute LD quantification.[85] Transcriptome analysis further revealed resmetirom-associated shifts toward increased expression of genes involved in glucose homeostasis, consistent with known effects of thyroid hormones on hepatic glucose metabolism.[86,87]

Several limitations should be acknowledged. First, the functional consequences of chromosomal abnormalities, particularly those involving chromosome 20, which harbors the hepatic transcriptional regulator HNF4α, remain unclear

and may influence hepatocyte-specific gene regulation. Second, our comparisons were conducted under standard culture conditions for each cell line. Although intracellular triglyceride levels were not affected by glucose concentrations of 11 mM vs 25 mM and did recapitulate previous literature,[15] transcriptional effects at non-physiological glucose levels (>7.8 mM) cannot be excluded.[88] For example, De Gottardi *et al* (2007) cultured immortalized human hepatocytes, HepG2, and Huh-7 cells in identical medium with dexamethasone and insulin.[89] Similar to Fa2N-4, LDs only formed with FFA supplementation, leading the authors to conclude that immortalized human hepatocytes are a superior model for hepatic steatosis. Third, inter-laboratory variability,[90] particularly for HepG2 cells, may influence transcriptomic,[14] drug response,[91] and metabolic outcomes,[73] underscoring the need for standardized conditions and cross-laboratory validation.

Conclusion

In summary, our findings demonstrate that the immortalized human hepatocyte cell line Fa2N-4 provides a reproducible, disease-relevant model for studying steatotic liver disease. Fa2N-4 cells more faithfully recapitulate MASLD-associated transcriptional and lipid-accumulation features and pharmacological responsiveness to resmetirom. These results present an alternative hepatocyte cell line model for MASLD and emphasize the need for careful model characterization to study human disease biology. Moreover, using Fa2N-4 cells, we identified a previously unrecognized resmetirom-induced metabolic shift in glucose homeostasis-related gene expression that accompanies its anti-steatotic effects. Mechanistic insights into the molecular adaptations induced by resmetirom aid understanding of both the pathophysiology and effective targets of anti-steatotic treatments. Together, these insights may contribute to improved preclinical modeling and inform personalized therapeutic strategies to reduce the global burden of MASLD.

Declaration of Generative AI and AI-Assisted Technologies in the Writing Process

During the preparation of this work, the author(s) used Elicit (Elicit: The AI Research Assistant, https://elicit.com, accessed 26 October 2024) for the purpose of finding scientific research papers and Grammarly (2024, the Grammarly Handbook, https://www.grammarly.com/handbook/, accessed on 18 December 2025) for grammar and spelling checks. After using this tool/service, the author(s) reviewed and edited the content as needed and take(s) full responsibility for the content of the publication.

Supplementary Materials

Material associated with this article can be found, in the online version, at https://doi.org/10.1016/j.gastha.2026.101069.

References

1. Riazi K, Azhari H, Charette JH, et al. The prevalence and incidence of NAFLD Worldwide: a systematic review and meta-analysis. Lancet Gastroenterol Hepatol 2022;7:851–861.
2. Diehl AM, Day C. Cause, pathogenesis, and treatment of nonalcoholic steatohepatitis. N Engl J Med 2017;377:2063–2072.
3. Shen F, Zheng RD, Sun XQ, et al. Gut microbiota dysbiosis in patients with non-alcoholic fatty liver disease. Hepatobiliary Pancreat Dis Int 2017;16:375–381.
4. Stefan N, Häring HU, Cusi K. Non-alcoholic fatty liver disease: causes, diagnosis, cardiometabolic consequences, and treatment strategies. Lancet Diabetes Endocrinol 2019;7:313–324.
5. Kirpich IA, Marsano LS, McClain CJ. Gut-liver axis, nutrition, and non-alcoholic fatty liver disease. Clin Biochem 2015;48:923–930.
6. Dufour JF, Anstee QM, Bugianesi E, et al. Current therapies and new developments in NASH. Gut 2022;71:2123–2134.
7. Anon. FDA approves first MASH drug. Nat Biotechnol 2024;42:540.
8. Newsome PN, Buchholtz K, Cusi K, et al. A placebo-controlled trial of subcutaneous semaglutide in nonalcoholic steatohepatitis. N Engl J Med 2021;384:1113–1124.
9. Food and Drug Administration from U.S.A. FDA approves treatment for serious liver disease known as 'MASH'. FDA, 2025.
10. Sanyal AJ, Newsome PN, Kliers I, et al. Phase 3 trial of semaglutide in metabolic dysfunction–associated steatohepatitis. N Engl J Med 2025;392:2089–2099.
11. Harrison SA, Bedossa P, Guy CD, et al. A phase 3, randomized, controlled trial of resmetirom in NASH with liver fibrosis. N Engl J Med 2024;390:497–509.
12. Vacca M, Kamzolas I, Harder LM, et al. An unbiased ranking of murine dietary models based on their proximity to human Metabolic dysfunction-Associated Steatotic Liver Disease (MASLD). Nat Metab 2024;6:1178–1196.
13. Ramos MJ, Bandiera L, Menolascina F, et al. In vitro models for non-alcoholic fatty liver disease: Emerging platforms and their applications. iScience 2022;25:103549.
14. Arzumanian V, Pyatnitskiy M, Poverennaya E. Comparative transcriptomic analysis of three common liver cell lines. Int J Mol Sci 2023;24:8791.
15. Huggett ZJ, Smith A, de Vivo N, et al. A comparison of primary human hepatocytes and hepatoma cell lines to model the effects of fatty acids, fructose and glucose on liver cell lipid accumulation. Nutrients 2023;15:40.

16. Baselli GA, Jamialahmadi O, Pelusi S, et al. Rare ATG7 genetic variants predispose patients to severe fatty liver disease. J Hepatol 2022;77:596–606.
17. Dutta AK. Adiponutrin (PNPLA3) in liver fibrogenesis: is unaltered HepG2 cell line a better model system compared to murine models? Med Hypotheses 2015; 85:736–739.
18. Mills JB, Rose KA, Sadagopan N, et al. Induction of drug metabolism enzymes and MDR1 using a novel human hepatocyte cell line. J Pharmacol Exp Ther 2004;309:303–309.
19. Kenny JR, Chen L, McGinnity DF, et al. Efficient assessment of the utility of immortalized Fa2N-4 cells for cytochrome P450 (CYP) induction studies using multiplex quantitative reverse transcriptase-polymerase chain reaction (qRT-PCR) and substrate cassette methodologies. Xenobiotica 2008;38:1500–1517.
20. Ripp SL, Mills JB, Fahmi OA, et al. Use of immortalized human hepatocytes to predict the magnitude of clinical drug-drug interactions caused by CYP3A4 induction. Drug Metab Dispos 2006;34:1742–1748.
21. Ramboer E, de Craene B, de Kock J, et al. Strategies for immortalization of primary hepatocytes. J Hepatol 2014; 61:925.
22. Ni M, He J, Li T, et al. Establishment and characterization of SV40 T-Antigen immortalized porcine muscle satellite cell. Cells 2024;13:703.
23. May T, Hauser H, Wirth D. Transcriptional control of SV40 T-antigen expression allows a complete reversion of immortalization. Nucleic Acids Res 2004; 32:5529–5538.
24. Hariparsad N, Carr BA, Evers R, et al. Comparison of immortalized Fa2N-4 cells and human hepatocytes as in vitro models for cytochrome P450 induction. Drug Metab Dispos 2008;36:1046–1055.
25. Li BH, He FP, Yang X, et al. Steatosis induced CCL5 contributes to early-stage liver fibrosis in nonalcoholic fatty liver disease progress. Translational Res 2017; 180:103–117.e4.
26. Smith PK, Krohn RI, Hermanson GT, et al. Measurement of protein using bicinchoninic acid. Anal Biochem 1985; 150:76–85.
27. Sündermann A, Eggers LF, Schwudke D. Liquid extraction: Bligh and dyer. In: Encyclopedia of lipidomics. Netherlands: Springer, 2016:1–4.
28. Bankhead P, Loughrey MB, Fernández JA, et al. QuPath: open source software for digital pathology image analysis. Sci Rep 2017;7(1):1–7.
29. Schmidt U, Weigert M, Broaddus C, et al. Cell detection with star-convex polygons:Lecture notes in computer science (including subseries lecture notes in artificial intelligence and lecture notes in bioinformatics) 2018;11071 LNCS; 2018:265–273.
30. Love MI, Huber W, Anders S. Moderated estimation of fold change and dispersion for RNA-seq data with DESeq2. Genome Biol 2014;15:1–21.
31. Draghici S, Khatri P, Tarca AL, et al. A systems biology approach for pathway level analysis. Genome Res 2007; 17:1537–1545.
32. Donato M, Xu Z, Tomoiaga A, et al. Analysis and correction of crosstalk effects in pathway analysis. Genome Res 2013;23:1885–1893.
33. Yu G, Wang LG, Han Y, et al. clusterProfiler: an R package for comparing biological themes among gene clusters. OMICS 2012;16:284.
34. Kiyono T, Foster SA, Koop JI, et al. Both Rb/p16(INK4a) inactivation and telomerase activity are required to immortalize human epithelial cells. Nature 1998; 396:84–88.
35. Toouli CD, Huschtscha LI, Neumann AA, et al. Comparison of human mammary epithelial cells immortalized by simian virus 40 T-Antigen or by the telomerase catalytic subunit. Oncogene 2002;21:128–139.
36. Trakarnsanga K, Griffiths RE, Wilson MC, et al. An immortalized adult human erythroid line facilitates sustainable and scalable generation of functional red cells. Nat Commun 2017;8:14750.
37. Loomba R, Schork N, Chen CH, et al. Heritability of hepatic fibrosis and steatosis based on a prospective twin study. Gastroenterology 2015;149:1784.
38. Speliotes EK, Yerges-Armstrong LM, Wu J, et al. Genome-wide association analysis identifies variants associated with nonalcoholic fatty liver disease that have distinct effects on metabolic traits. PLoS Genet 2011;7:e1001324.
39. Palmer ND, Musani SK, Yerges-Armstrong LM, et al. Characterization of european-ancestry NAFLD-associated variants in individuals of African and Hispanic descent. Hepatology 2013;58:966.
40. Zhou B, Ho SS, Greer SU, et al. Haplotype-resolved and integrated genome analysis of the cancer cell line HepG2. Nucleic Acids Res 2019;47:3846.
41. Balcar L, Semmler G, Oberkofler H, et al. PNPLA3 is the dominant SNP linked to liver disease severity at time of first referral to a tertiary center. Dig Liver Dis 2022; 54:84–90.
42. Chen Y, Du X, Kuppa A, et al. Genome-wide association meta-analysis identifies 17 loci associated with nonalcoholic fatty liver disease. Nat Genet 2023; 55(10):1640–1650.
43. Sookoian S, Rotman Y, Valenti L. Genetics of metabolic dysfunction-associated steatotic liver disease: the state of the art update. Clin Gastroenterol Hepatol 2024; 22:2177–2187.e3.
44. Sveinbjornsson G, Ulfarsson MO, Thorolfsdottir RB, et al. Multiomics study of nonalcoholic fatty liver disease. Nat Genet 2022;54(11):1652–1663.
45. Li Y, van den Berg EH, Kurilshikov A, et al. Genome-wide studies reveal genetic risk factors for hepatic fat content. Genomics Proteomics Bioinformatics 2024;22: qzae031.
46. Rehm HL, Berg JS, Brooks LD, et al. ClinGen — the clinical genome resource. N Engl J Med 2015; 372:2235–2242.
47. Richards S, Aziz N, Bale S, et al. Standards and guidelines for the interpretation of sequence variants: a joint consensus recommendation of the American College of Medical Genetics and Genomics and the Association for Molecular Pathology. Genet Med 2015;17:405–424.
48. Byrska-Bishop M, Evani US, Zhao X, et al. High-coverage whole-genome sequencing of the expanded 1000 genomes project cohort including 602 trios. Cell 2022;185:3426–3440.e19.

49. Kent JW. Rare variants, common markers: synthetic association and beyond. Genet Epidemiol 2011; 35(Suppl 1):S80–S84.
50. Huebbe P, Bilke S, Rueter J, et al. Human APOE4 protects high-fat and high-sucrose diet fed targeted replacement mice against fatty liver disease compared to APOE3. Aging Dis 2024;15:259.
51. Tsai TH, Chen E, Li L, et al. The constitutive lipid droplet protein PLIN2 regulates autophagy in liver. Autophagy 2017;13:1130–1144.
52. Harrison SA. Use of resmetirom in patients with metabolic dysfunction-associated steatohepatitis. Gastroenterol Hepatol (N Y) 2024;20:355.
53. Sinha RA, Singh BK, Yen PM. Direct effects of thyroid hormones on hepatic lipid metabolism. Nat Rev Endocrinol 2018;14:259.
54. Wang X, Wang L, Geng L, et al. Resmetirom ameliorates NASH-model mice by suppressing STAT3 and NF-κB signaling pathways in an RGS5-Dependent manner. Int J Mol Sci 2023;24:5843.
55. Maimouni S, Issa N, Cheng S, et al. Tumor suppressor RARRES1- A novel regulator of fatty acid metabolism in epithelial cells. PLoS One 2018;13:e0208756.
56. Rachfal AW, Brigstock DR. Connective tissue growth factor (CTGF/CCN2) in hepatic fibrosis. Hepatol Res 2003;26:1–9.
57. Liao X, Bu Y, Jiang S, et al. CCN2–MAPK–Id-1 loop feedback amplification is involved in maintaining stemness in oxaliplatin-resistant hepatocellular carcinoma. Hepatol Int 2019;13(4):440–453.
58. Zhang RR, Zheng YW, Li B, et al. Hepatic stem cells with self-renewal and liver repopulation potential are harbored in CDCP1-positive subpopulations of human fetal liver cells. Stem Cell Res Ther 2018;9(1):29.
59. Kim HY, Baek GH, Lee W, et al. CD44 is involved in liver regeneration through enhanced uptake of extracellular cystine. Clin Transl Med 2022;12:e873.
60. Nakagawa Y, Satoh A, Tezuka H, et al. CREB3L3 controls fatty acid oxidation and ketogenesis in synergy with PPARα. Sci Rep 2016;6(1):39182.
61. Papachristoforou E, Kong K, Colella F, et al. A human single-cell atlas identifies OLR1+ scar-associated macrophages as a therapeutic target for chronic liver disease. bioRxiv 2025 August 28 [Epubahead of print].
62. Annadurai P, Isaac AE. Unveiling the role of IL7R in metabolism-associated fatty liver disease leading to hepatocellular carcinoma through transcriptomic and machine learning approaches. Discov Oncol 2025;16:873.
63. McAllaster MR, Bhushan J, Balce DR, et al. Autophagy gene-dependent intracellular immunity triggered by interferon-γ. mBio 2023;14:e0233223.
64. Friedline RH, Noh HL, Suk S, et al. IFNγ-IL12 axis regulates intercellular crosstalk in metabolic dysfunction-associated steatotic liver disease. Nat Commun 2024; 15(1):5506.
65. Monson EA, Crosse KM, Duan M, et al. Intracellular lipid droplet accumulation occurs early following viral infection and is required for an efficient interferon response. Nat Commun 2021;12:4303.
66. Gunn PJ, Green CJ, Pramfalk C, et al. In vitro cellular models of human hepatic fatty acid metabolism: differences between Huh7 and HepG2 cell lines in human and fetal bovine culturing serum. Physiol Rep 2017;5: e13532.
67. Salomone F, Pipitone RM, Longo M, et al. SIRT5 rs12216101 T>G variant is associated with liver damage and mitochondrial dysfunction in patients with non-alcoholic fatty liver disease. J Hepatol 2024;80:10–19.
68. Ma Y, Belyaeva OV, Brown PM, et al. 17-Beta hydroxysteroid dehydrogenase 13 is a hepatic retinol dehydrogenase associated with histological features of nonalcoholic fatty liver disease. Hepatology 2019; 69:1504–1519.
69. Umano GR, Caprio S, di Sessa A, et al. The rs626283 variant in the MBOAT7 gene is associated with insulin resistance and fatty liver in Caucasian obese youth. Am J Gastroenterol 2018;113:376–383.
70. Mancina RM, Dongiovanni P, Petta S, et al. The MBOAT7-TMC4 variant rs641738 increases risk of nonalcoholic fatty liver disease in individuals of European descent. Gastroenterology 2016;150:1219.
71. Arzumanian VA, Kiseleva OI, Poverennaya EV. The curious case of the HepG2 cell line: 40 years of expertise. Int J Mol Sci 2021;22:13135.
72. Ardisasmita AI, Schene IF, Joore IP, et al. A comprehensive transcriptomic comparison of hepatocyte model systems improves selection of models for experimental use. Commun Biol 2022;5(1):1094.
73. Palasantzas VEJM, Struik D, Bos T, et al. Assessing nutraceuticals for hepatic steatosis: a standardized in vitro approach. Nutrients 2026;18:388.
74. Liu W, Anstee QM, Wang X, et al. Transcriptional regulation of PNPLA3 and its impact on susceptibility to Nonalcoholic Fatty Liver Disease (NAFLD) in humans. Aging (Albany NY) 2016;9:26.
75. Zain SM, Mohamed Z, Mohamed R. Common variant in the glucokinase regulatory gene rs780094 and risk of nonalcoholic fatty liver disease: a meta-analysis. J Gastroenterol Hepatol 2015;30:21–27.
76. Kendall TJ, Jimenez-Ramos M, Turner F, et al. An integrated gene-to-outcome multimodal database for metabolic dysfunction-associated steatotic liver disease. Nat Med 2023;29:2939–2953.
77. Park S, Lee S-H, Han S, et al. Paired snRNA-seq and scRNA-seq analysis of MASLD patients to identify early-stage markers for disease progression. Hepatol Commun 2025;9:e0820.
78. Kwon Y, Gottmann P, Wang S, et al. Induction of steatosis in primary human hepatocytes recapitulates key pathophysiological aspects of metabolic dysfunction-associated steatotic liver disease. J Hepatol 2025; 82:18–27.
79. Atanasovska B, Rensen SS, Marsman G, et al. Long non-coding RNAs involved in progression of non-alcoholic fatty liver disease to steatohepatitis. Cells 2021;10:1883.
80. Errafii K, Khalifa O, Al-Akl NS, et al. Comparative transcriptome analysis reveals that Exendin-4 improves steatosis in HepG2 cells by modulating signaling pathways related to lipid metabolism. Biomedicines 2022;10:1020.
81. de la Morena DG. Probiotic potential and antimicrobial activity of Bacteroidales strains to fight fatty liver disease. Thesis fully internal (DIV), University of Groningen, 2024.

82. Ritter MJ, Amano I, Hollenberg AN. Thyroid hormone signaling and the liver. Hepatology 2020;72:742–752.
83. Ratziu V, Scanlan TS, Bruinstroop E. Thyroid hormone receptor-β analogues for the treatment of metabolic dysfunction-associated steatohepatitis (MASH). J Hepatol 2025;82:375–387.
84. Li J, Ayada I, Pan Q. Resmetirom directly inhibits lipid accumulation in human liver-derived organoids. J Hepatol 2025;83:e86–e87.
85. Strauss JA, Shepherd DA, Macey M, et al. Divergence exists in the subcellular distribution of intramuscular triglyceride in human skeletal muscle dependent on the choice of lipid dye. Histochem Cell Biol 2020; 154(4):369–382.
86. Baquer NZ, Cascales M, Mc Lean P, et al. Effects of thyroid hormone deficiency on the distribution of hepatic metabolites and control of pathways of carbohydrate metabolism in liver and adipose tissue of the rat. Eur J Biochem 1976;68:403–413.
87. Minderop RH, Hoeppner W, Seitz HJ. Regulation of hepatic glucokinase gene expression: role of carbohydrates, and glucocorticoid and thyroid hormones. Eur J Biochem 1987;164:181–187.
88. Mc Kee TJ, Komarova SV. Is it time to reinvent basic cell culture medium? Am J Physiol Cell Physiol 2017; 312:C624–C626.
89. de Gottardi A, Vinciguerra M, Sgroi A, et al. Microarray analyses and molecular profiling of steatosis induction in immortalized human hepatocytes. Lab Invest 2007; 87:792–806.
90. Tyakht AV, Ilina EN, Alexeev DG, et al. RNA-Seq gene expression profiling of HepG2 cells: the influence of experimental factors and comparison with liver tissue. BMC Genomics 2014;15:1–9.
91. Sison-Young RL, Lauschke VM, Johann E, et al. A multicenter assessment of single-cell models aligned to standard measures of cell health for prediction of acute hepatotoxicity. Arch Toxicol 2017;91:1385–1400.

Received March 5, 2026. Accepted July 14, 2026.

Correspondence:
Address correspondence to: Johan W. Jonker, PhD, Department of Pediatrics, University Medical Center Groningen, Hanzeplein 1, 9713 Groningen, The Netherlands. e-mail: j.w.jonker@umcg.nl.

Acknowledgments:
The authors would like to thank Roos Eilers, Alfredo Rios-Ocampo, Tim van Zutphen, Sofija Stojanovic, Rutger Moddermans, Jelle Slager, and Daoming Wang for their technical support. Furthermore, the authors thank Kate McIntyre (PhD, in-house editor for the Department of Genetics at the University Medical Center Groninge, the Netherlands) for editing and reviewing the manuscript prior to submission. The graphic abstract was created in BioRender. Palasantzas, V. (2026) (https://BioRender.com/tqc205r). Part of the work has been performed at the UMCG Imaging and Microscopy Center, which is sponsored by NL-BioImaging Infrastructure (NL-BI, NWO 184.036.012).

Authors' Contributions:
Victoria E.J.M. Palasantzas: Main design and direction; performed the in vitro experiments, processed the experimental data, performed the analyses, performed histology staining on coverslips, and analyzed histological features and staining; writing – original draft. Dicky Struik: Processed the experimental data, performed the analyses, and reviewed the manuscript at several stages. Trijnie Bos: Measured the intracellular triglycerides and performed the initial analyses. Mirjam H. Koster: Scanned the coverslips to create histological images. Jody Gelderloos-Arends: Facilitated and performed the global screen array. Ellen R. Vos: Performed the karyotyping. Krista K. van Dijk-Bos: Facilitated and organized the global screening array analysis from direction to data processing. Sebo Withoff: Designed and directed the experimental work and reviewed the analyzed data. Jingyuan Fu: Designed and directed the experimental work and reviewed the data and manuscript. Joanne A. Hoogerland: Designed and directed the experimental work, analyzed and interpreted data analysis, and reviewed the manuscript. Johan W. Jonker: Acquisition of funding, designed and directed the experimental work, analyzed and interpreted data analysis, and wrote the manuscript. All authors discussed the results and commented on the manuscript.

Conflicts of Interest:
The authors disclose no conflicts.

Funding:
Victoria E.J.M. Palasantzas received a PhD fellowship from the Graduate School of Medical Sciences, University of Groningen. Sebo Withoff is supported by the Netherlands Organ-on-Chip Initiative, a Netherlands Organization for Scientific Research (NWO) Gravitation project (024.003.001) funded by the Ministry of Education, Culture, and Science of the government of the Netherlands. Johan W. Jonker is supported by NWO VICI grant 016.176.640. Jingyuan Fu is supported by a European Research Council Consolidator grant (grant agreement No. 101001678), an NWO VICI grant (VI.C.202.022), an NWO KIC grant (KICH1.LWV04.21.013), the AMMODO Science Award 2023 for Biomedical Sciences from Stichting Ammodo, and the Dutch Heart Foundation AtheroNeth project.

Ethical Statement:
This study did not involve human participants, human biological material, or animal subjects; therefore, no ethical considerations are required for this study.

Data Transparency Statement:
All data, analytic methods, and study materials will be made available to other researchers upon reasonable request. The authors did not preregister the research with an analysis plan in an independent institutional registry. All authors had access to the study data and have reviewed and approved the final manuscript. The raw bulk RNA sequencing data are submitted to NCBI's GEO under accession number GSE324401.

Reporting Guidelines:
STROPS guideline, ISCN guideline.

Gastro Hep Advances, Volume 5

Supplemental information

Evaluation of the Immortalized Primary Human Hepatocyte Cell Line Fa2N-4 as a Model for Metabolic Dysfunction–Associated Steatotic Liver Disease

Victoria E.J.M. Palasantzas, Dicky Struik, Trijnie Bos, Mirjam H. Koster, Jody Gelderloos-Arends, Ellen R. Vos, Krista K. van Dijk-Bos, Sebo Withoff, Jingyuan Fu, Joanne A. Hoogerland, and Johan W. Jonker

OVERVIEW SUPPLEMENTAL TABLE AND FIGURES

Supplementary File 1 (below)

Supplementary Table 1: Genetic variation in known MASLD single nucleotide polymorphisms (SNPs) identified in hepatic cell lines.......... 4

Supplementary Figure S1. Basal glucose concentration in HepG2 and Fa2N-4 cell media does not change intracellular triglycerides. 5

Supplementary Figure S2. Quantification of Oil red O stainings in QuPath with the StarDist module.......... 6

Supplementary Figure S3. Cell viability of HepG2 cells after 24 h treatment with resmetirom. 7

Supplementary File 2 (separate .xsls)

Table 1 - HepG2 PEA steatosis

Table 2 - Fa2N-4 PEA steatosis

Table 3 - HepG2 DEGs

Table 4 - FA2N-4 DEGs

Table 5 - DEGs HepG2 vs. Fa2N-4

Table 6 -Fa2N-4+RSM Glyc

Supplementary File 3 Karyograms (below)

Reports and original data (.pdf and .png)

Characteristics known SNPs present in SNP array<br>Used dbSNP NCBI[77] and literature[36–40,78]								HepG2<br>South American White descent, XY, 15 years				Fa2N-4<br>Unknown White descent, XX, 12 years			
**CHR**	**Locus**	**SNP ID**	**REF**	**ALT**	**SNP Effect**	**ALT Allele frequency**[41]	**Variant effect for MASLD**	**Result**	**Genotype**	**GT Quality**	**Risk allele**	**Result**	**Geno type**	**GT Quality**	**Risk allele**
2	*COBLL1 / GRB14*	rs13389219	C	T	Intronic variant	0.4080	Increase	Heterozygote ALT	0/1	89%	+1	Homozygote REF	0/0	93%	0
2	*GCKR*	rs780094	T	C	Intronic variant	0.7049	Increase	Heterozygote ALT	0/1	70%	+1	Heterozygote ALT	0/1	58%	+1
2	*GCKR*	rs1260326	T	C	P446L	0.7130	Increase	missing call	./.	6%	.	Heterozygote ALT	0/1	35%	+1
3	*ATG7*	rs36117895	T	C	V471A	0.0298	Increase	Heterozygote ALT	0/1	80%	+1	Homozygote REF	0/0	80%	0
3	*PPARG*	rs17036160	C	T	P12A	0.0670	Increase	Homozygote REF	0/0	83%	0	Homozygote REF	0/0	83%	0
4	*ADH1B*	rs1229984	T	C	H48R	0.8535	Decrease	Homozygote ALT	1/1	59%	0	Homozygote ALT	1/1	59%	0
4	*HSD17B13*	rs6834314	A	G	Intergenic	0.2299	Increase	Homozygote REF	0/0	70%	0	Heterozygote ALT	0/1	70%	+1
6	*HFE*	rs1800562	G	A	C282Y	0.0122	Increase	Homozygote REF	0/0	93%	0	Homozygote REF	0/0	93%	0
6	*HFE*	rs1799945	C	G	H63D	0.0739	Increase	Homozygote REF	0/0	87%	0	Homozygote REF	0/0	87%	0
6	*SIRT5*	rs12216101	T	G	Intronic	0.2174	Increase	Heterozygote ALT	0/1	36%	+1	Homozygote REF	0/0	87%	0
11	*PNPLA2*	rs140201358	C	G	N252K	0.0045	Increase	Homozygote REF	0/0	79%	0	Homozygote REF	0/0	79%	0
14	*SERPINA1*	rs28929474	C	T	E366K	0.0044	Increase	Homozygote REF	0/0	67%	0	Homozygote REF	0/0	67%	0
16	*FTO*	rs17817449	T	G	Intronic	0.3106	Increase	Homozygote REF	0/0	92%	0	Homozygote REF	0/0	37%	0
17	*APOH*	rs1801689	A	C	C325G	0.0183	Increase	Homozygote REF	0/0	76%	0	Homozygote REF	0/0	76%	0
19	*APOE*	rs429358	T	C	C130R	0.1512	Increase (Apo-ε3/ε3)	Homozygote REF	0/0	33%	0	Heterozygote ALT	0/1	33%	+1
		rs7412	C	T	R176C	0.0771	Decrease (Apo-ε4ε4, ε2ε4)	Homozygote REF<br>Genotype ®ε3ε3	0/0	70%	0	Heterozygote ALT<br>Genotype® ε2ε4	0/1	70%	+1
19	*MBOAT7 / TMC4*	rs8736	C	T	3 Prime UTR Variant – Upstream variant	0.6334	Increase	Homozygote REF	0/0	69%	0	Heterozygote ALT	0/1	69%	+1
19	*MBOAT7 / TMC4*	rs626283	G	C	Downstream - Upstream variant	0.6302	Increase	Homozygote REF	0/0	93%	0	Heterozygote ALT	0/1	93%	+1
19	*MBOAT7*	rs641738	C	T	Downstream variant - G17E	0.6287	Increase	Homozygote REF	0/0	82%	0	Heterozygote ALT	0/1	82%	+1
19	*TM6SF2*	rs58542926	C	T	E167K	0.0661	Increase	Homozygote REF	0/0	89%	0	Homozygote REF	0/0	89%	0
19	*TM6SF2*	rs187429064	A	G	L156P	0.0036	Increase	Homozygote REF	0/0	79%	0	Homozygote REF	0/0	79%	0
22	*PNPLA3*	rs738409	C	G	I148M	0.2644	Increase	Homozygote ALT	1/1	83%	+2	Homozygote ALT	1/1	83%	+2
22	*PNPLA3*	rs2896019	T	G	Intron variant	0.2626	Increase	Homozygote REF	0/0	85%	0	Homozygote ALT	1/1	85%	+2
22	*PNPLA3*	rs6006460	G	T	S453I	0.0336	Decrease	Homozygote REF	0/0	87%	0	Homozygote REF	0/0	87%	0
							**Total count of present risk alleles**				**6**				**12**

Supplementary Table 1: Genetic variation in known MASLD single nucleotide polymorphisms (SNPs) identified in hepatic cell lines. A multi-ethnic global screening array with the GRCh37 reference identified genetic variations in MASLD. Information derived from dbSNP NCBI for the annotated gene names, SNP effect, and alternative allele frequency based on 1000Genomes_30X Global[41]. The total count of risk alleles was calculated by the presence of disease-risk associated SNPs, amounting to +1 per present risk allele (in some cases, the reference allele is the risk allele, in other cases, an alternative). Abbreviations used: REF, reference allele; ALT, alternative allele; AF, allele frequency (of reference allele).

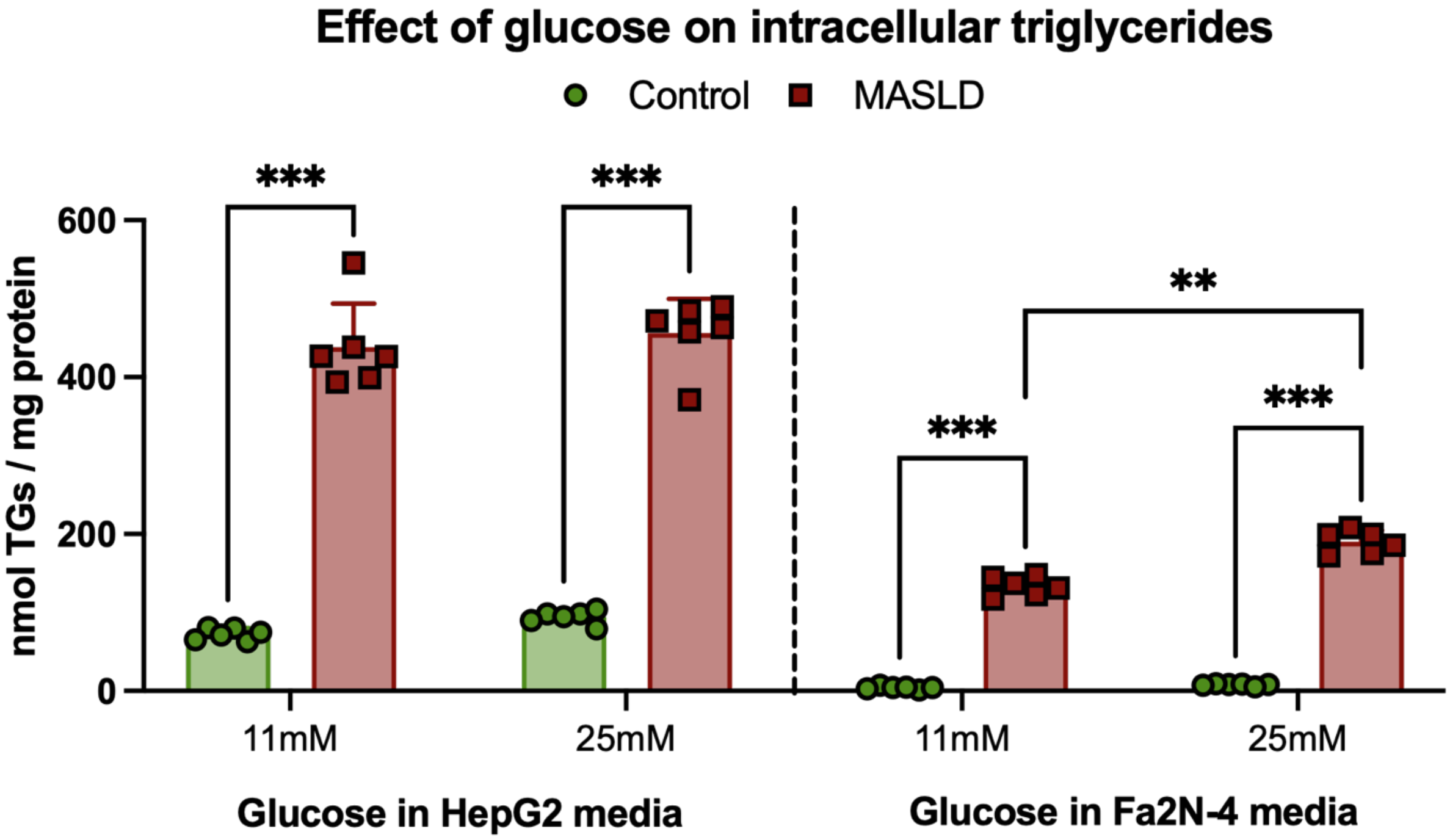

Supplementary Figure S1. Basal glucose concentration in HepG2 and Fa2N-4 cell media does not change intracellular triglycerides. Intracellular triglyceride levels of HepG2 and Fa2N-4 cells upon culturing in 11 mM and 25 mM glucose. 2-Way ANOVA test with *** $p < 0.001$ or ** $p < 0.01$ indicated.

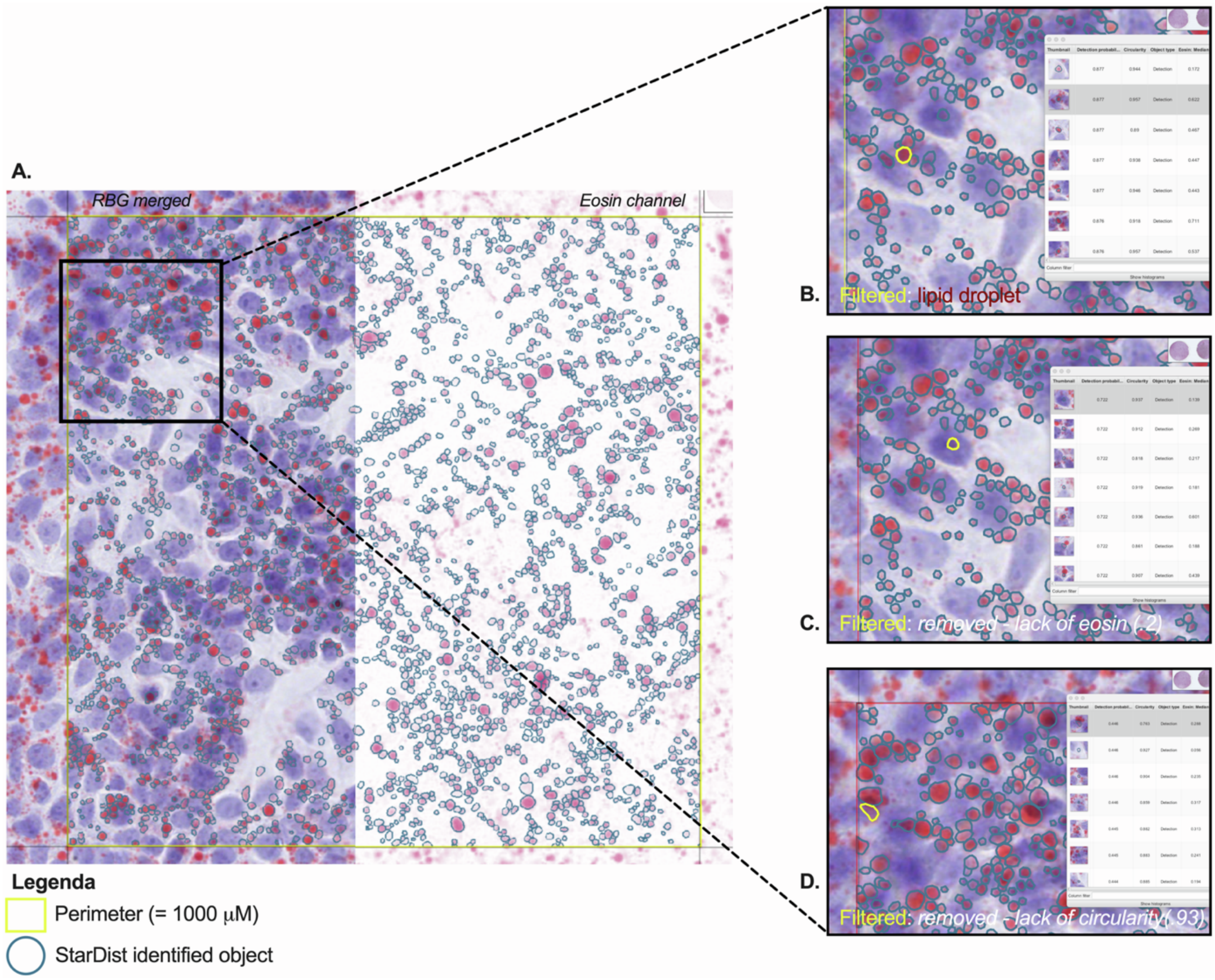

Supplementary Figure S2. Quantification of Oil red O stainings in QuPath with the StarDist module. (A) Representative image taken from a slide with steatotic HepG2 cells. On the right side, the merged RGB image is shown. On the left, the Eosin-only channel is displayed. (B) Example of an object identified as lipid droplet after applying filters on detection probability, circularity, and eosin channel. (C) Example of an object removed by filtering based on the lack of a threshold in the eosin channel of 0.2 and above. (D) Example of object removed by filtering based on lack of circularity threshold of 0.93 and above. Not shown is detection probability inclusion of ≥ 0.75.

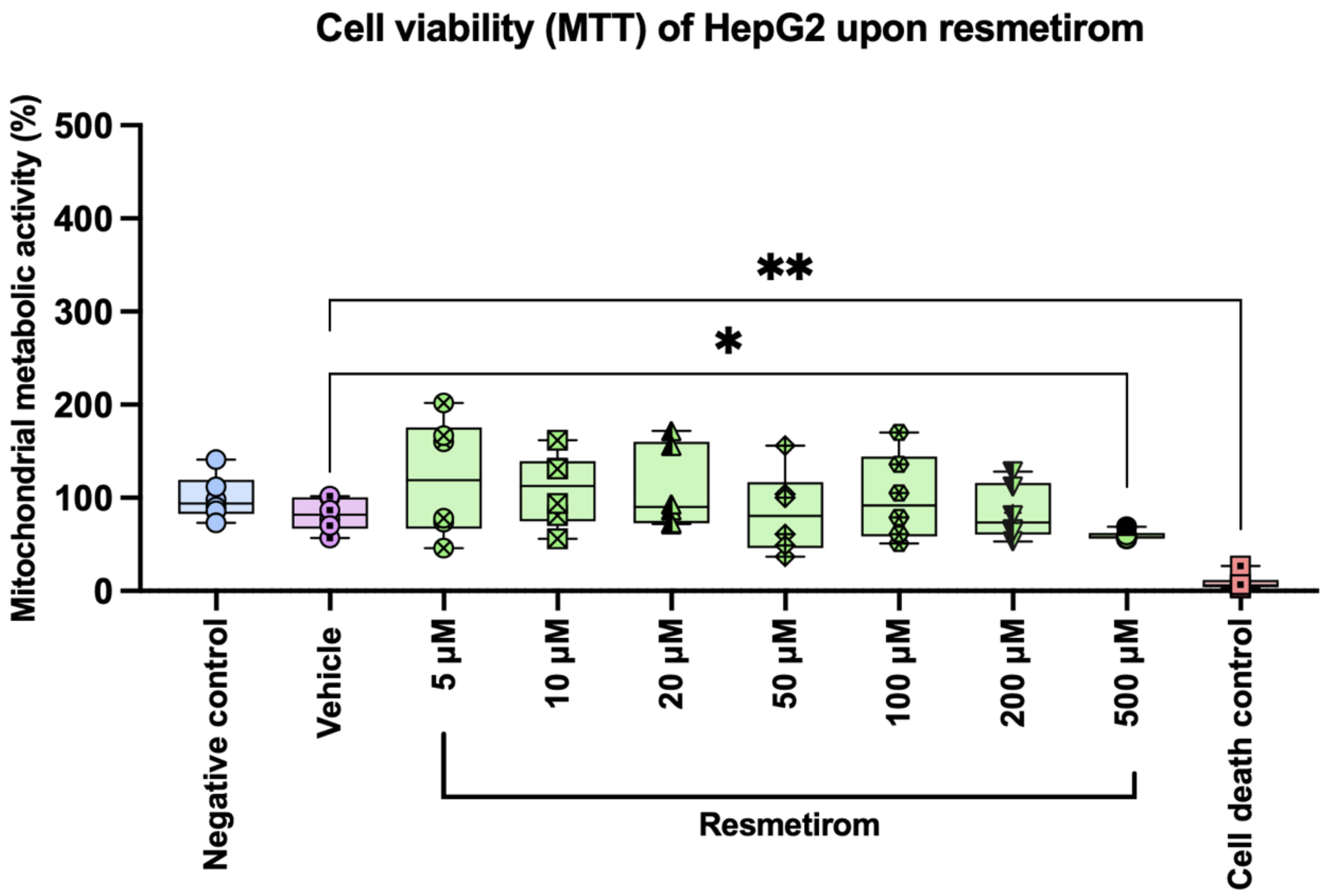

Supplementary Figure S3. Cell viability of HepG2 cells after 24 h treatment with resmetirom. Cell viability of HepG2 cells upon treatment with different resmetirom concentrations was examined by an MMT assay. Mann-Whitney test was performed, with * $p < 0.05$ and ** $p < 0.01$.

Case: HepG2

Patient ID: HepG2 p19
Patient Name:
Date of Birth:
Gender:
Clinical Indication:

Operator Name:
Specimen:
Received Date: 16-1-2025
Completed:

Case Notes: Analysis: 26-3-2025

Case Results: 51~52,XY,t(1;21)(p22;p11),+2,+16,add(16)(p13),+17,?add(17)(p11.2),+20,+20,+mar[cp10]

Final Results:

Microscope ID	

Cells Information:

#	Label	Slide-Cell:	Analysis State	X , Y	State By	Report	Notes	Results
1	>dgn	4-17	Karyotyped	18,64 X 8,66	EV	None		51,XY,t(1;21)(p22;p11),+2,9q-,+16,add(16)(p13),+17,?add(17)(p11.2)+20,+20,-21,+mar
2	>dgn	4-19	Karyotyped	18,04 X 8,96	EV	None		51,?X,Y,t(1;21)(p22;p11),+16,,add(16)(p13),+17,?add(17)9p11.2),+20,+20
3	>dgn	4-20	Karyotyped	10,63 X 9,15	EV	None		51,XY,t(1;21)(p22;p11),+2,+16,add(16)(p13),+17,?add(17)(p11.2)+20,+20,-21,+mar
4	>dgn	4-24	Karyotyped	17,18 X 10,18	EV	None		52,XY,t(1;21)(p22;p11),+2,+16,add(16)(p13),+17,?add(17)(p11.2),+20,+20,+mar
5	>dgn	5-7	Karyotyped	18,56 X 5,43	EV	None		52,XY,t(1;21)(p22;p11),+2,add(3)(p26),+16,add(16)(p13),+17,?add(17)(p11.2),+20,+20,+mar
6	>dgn	5-11	Karyotyped	3,62 X 6,12	EV	None		51,XY,t(1;21)(p22;p11),+2,+16,add(16)(p13),+17,?add(17)(p11.2),+20,+mar
7	>dgn	5-36	Karyotyped	2,25 X 23,34	EV	None		52,XY,t(1;21)(p22;p11),+2,+16,add(16)(p13),+17,?add(17)(p11.2),+20,+20,+mar
8	>dgn	5-37	Karyotyped	9,96 X 24,11	EV	None		52,XY,t(1;21)(p22;p11),+2,+16,add(16)(p13),del(16)(p11.2),+17,?add(17)p11),+20,+20,+mar
9	>dgn	6-26	Karyotyped	13,34 X 12,21	EV	None		52,XY,t(1;21)(p22;p11),+2,+16,add(16)(p13),+17,+?add(17)(p11.2),+20,+20,+mar
10	>dgn	6-28	Karyotyped	1,82 X 13,30	EV	None		50,XY,t(1;21)(p22;p11),+2,der(2)t(2;5)(q;q),-5,-13,+20,+20,+2mar

Karyotype Case: HepG2 Slide: >dgn(4) Cell: 17

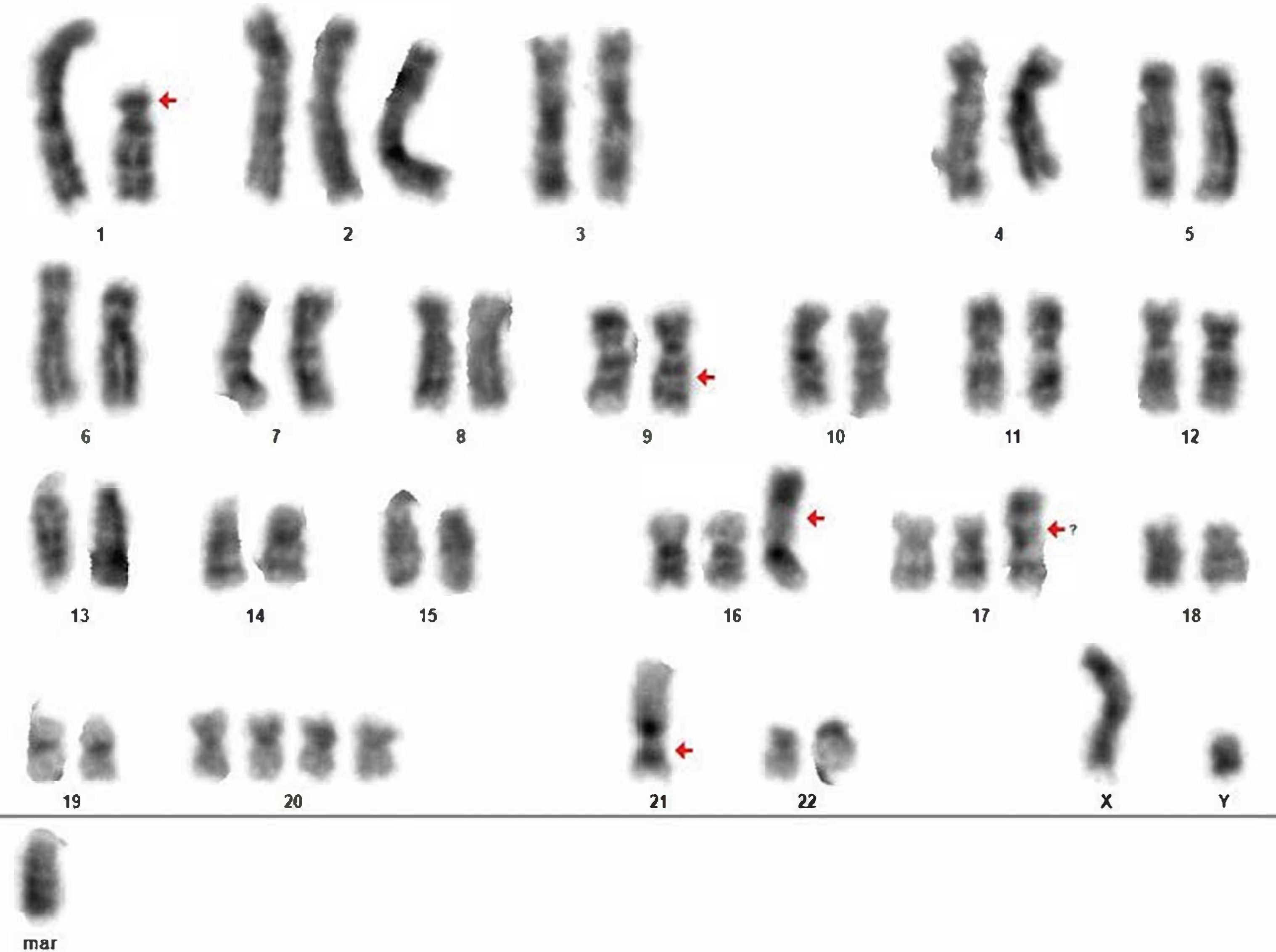

Karyotype Case: HepG2 Slide: >dgn(4) Cell: 19

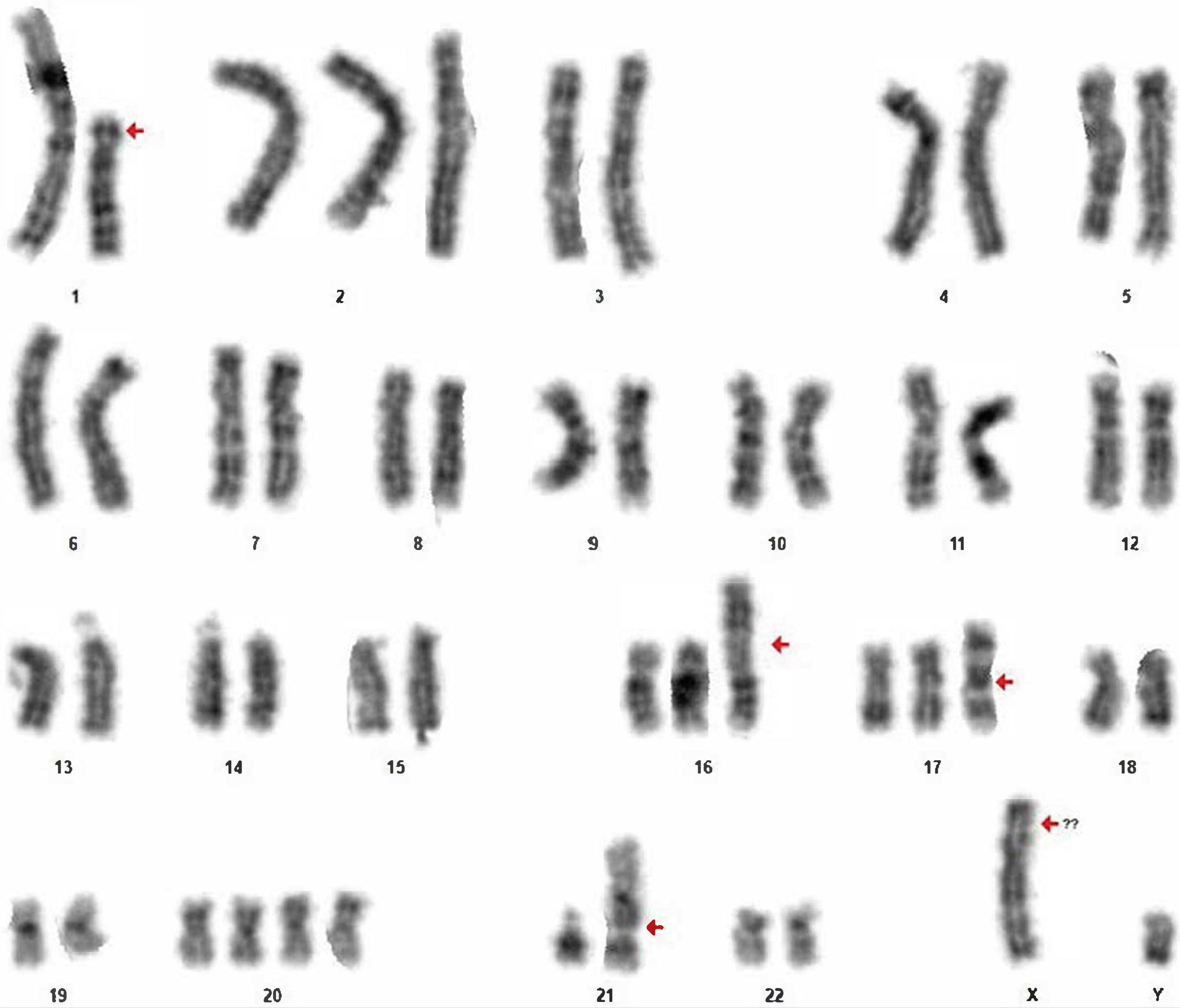

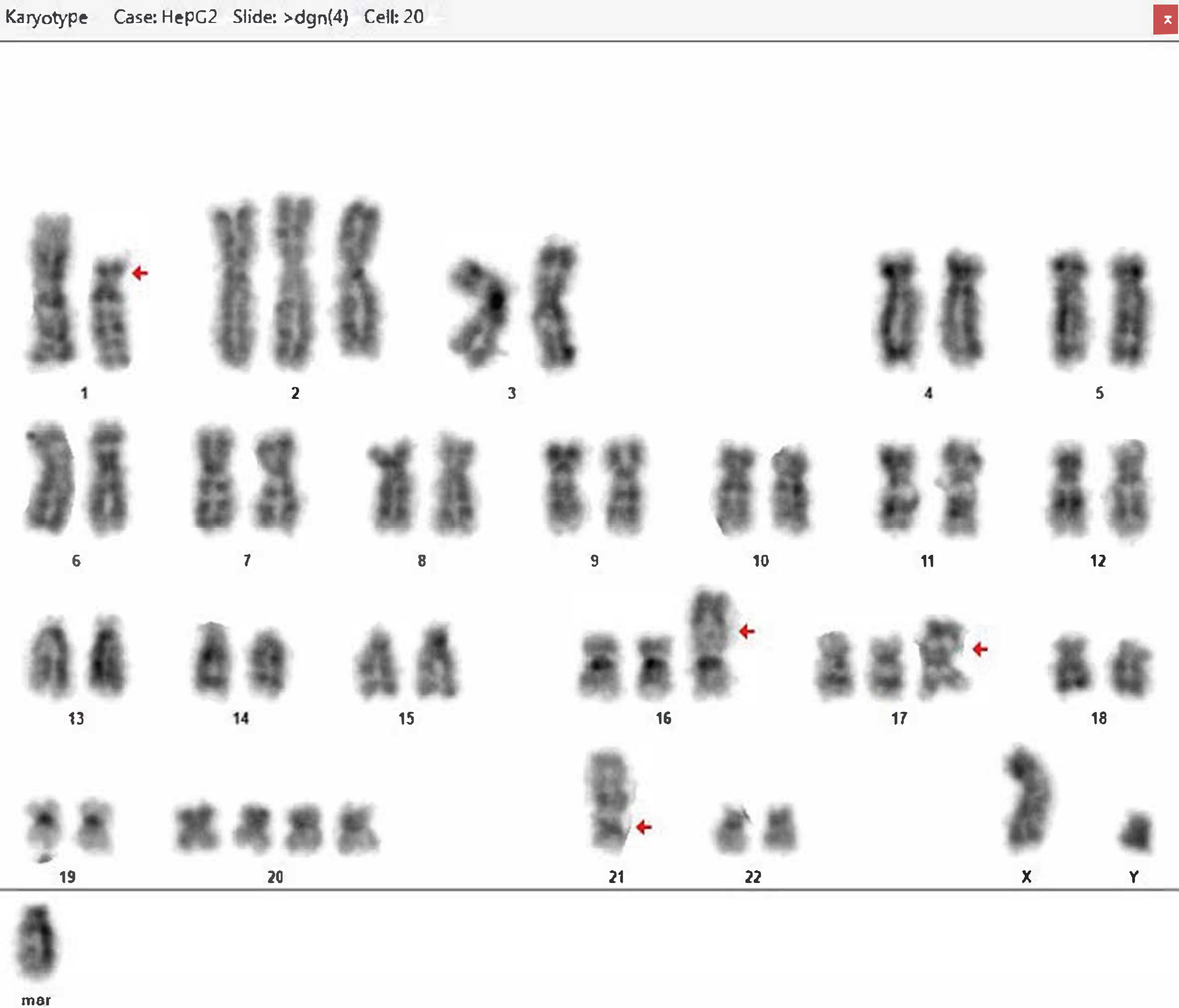

Karyotype Case: HepG2 Slide: >dgn(4) Cell: 20
1
2
3
4
5
6
7
8
9
10
11
12
13
14
15
16
17
18
19
20
21
22
X
Y
mar

Karyotype Case: HepG2 Slide: >dgn(4) Cell: 24

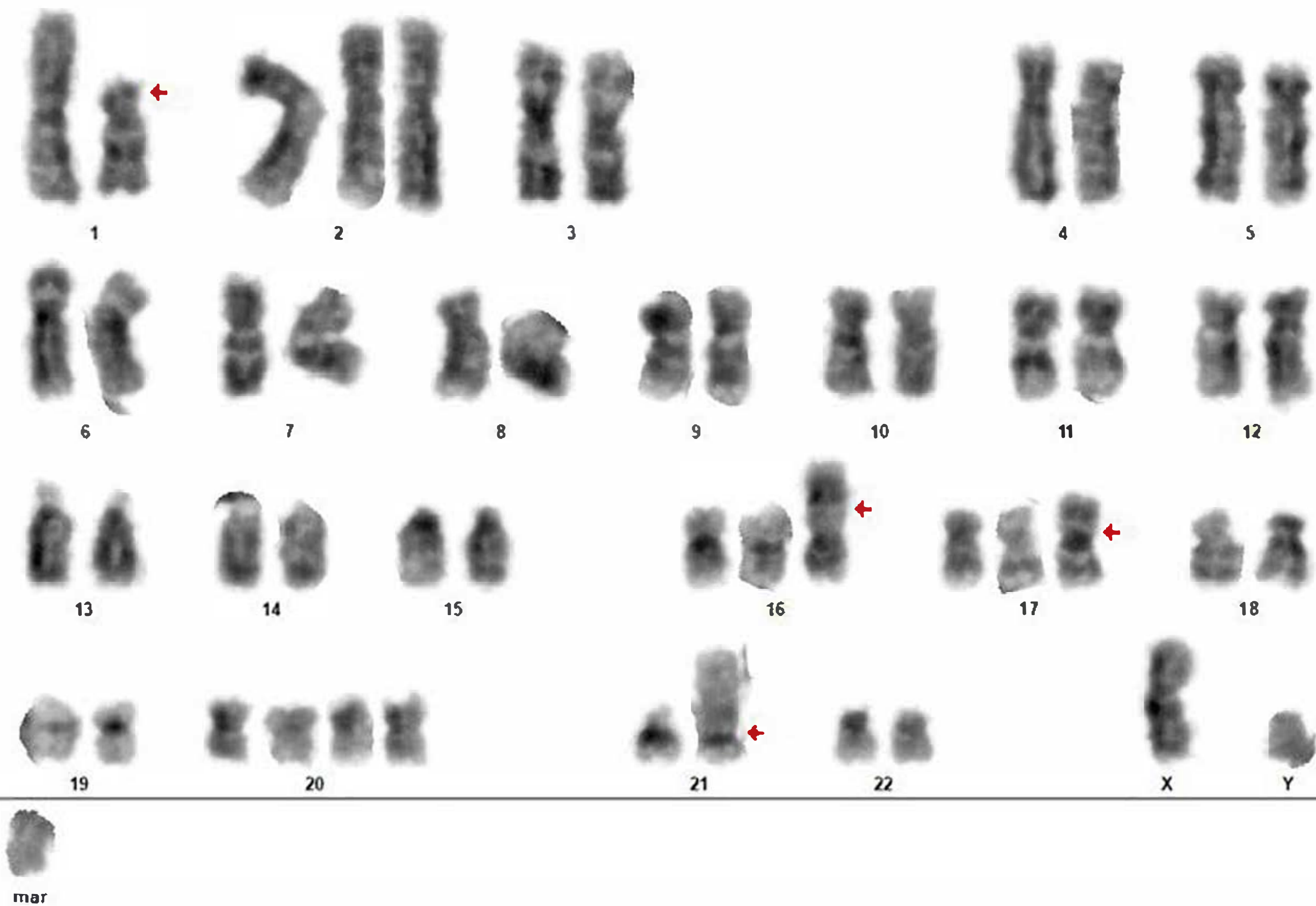

Karyotype Case: HepG2 Slide: >dgn(5) Cell: 7

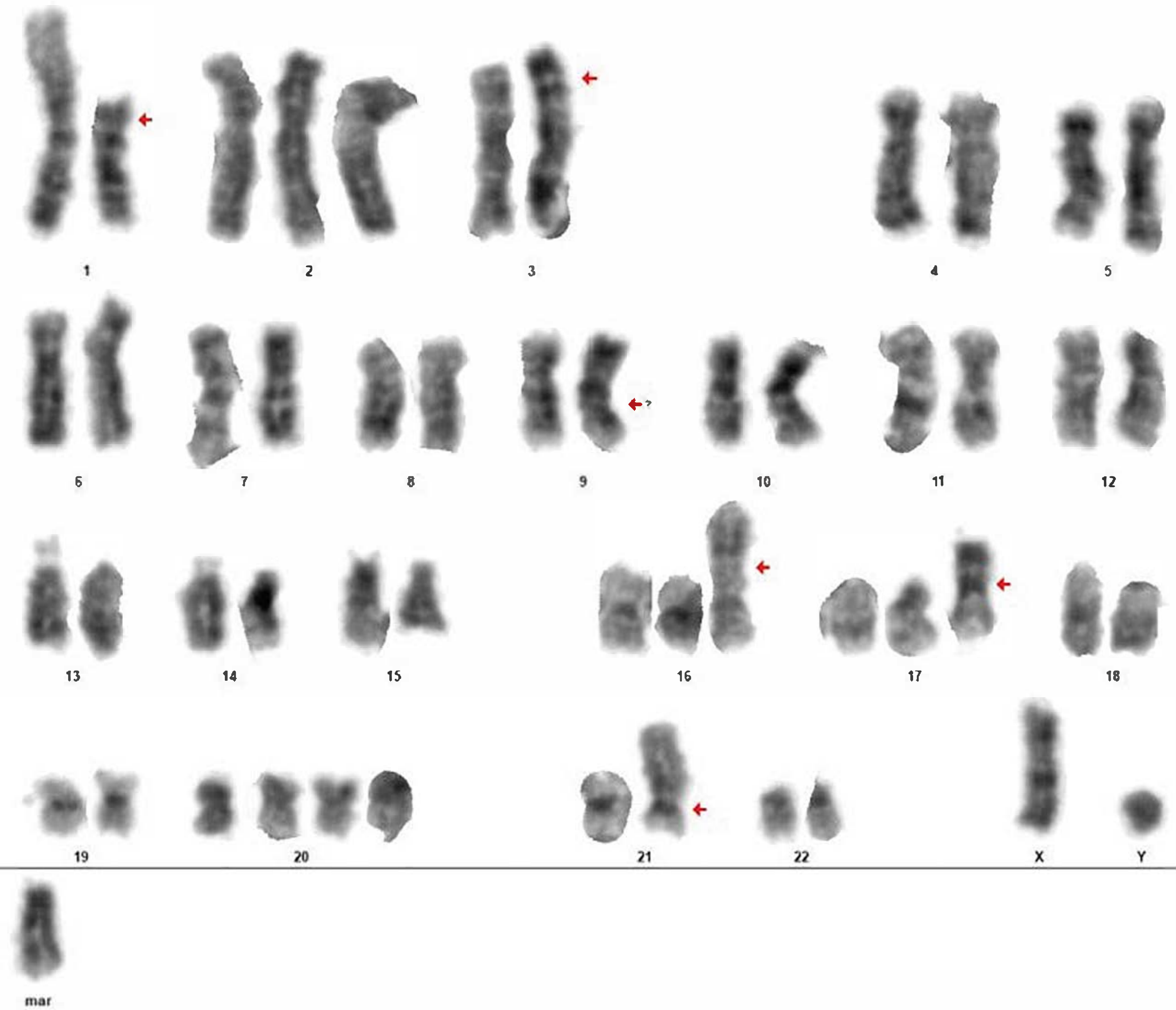

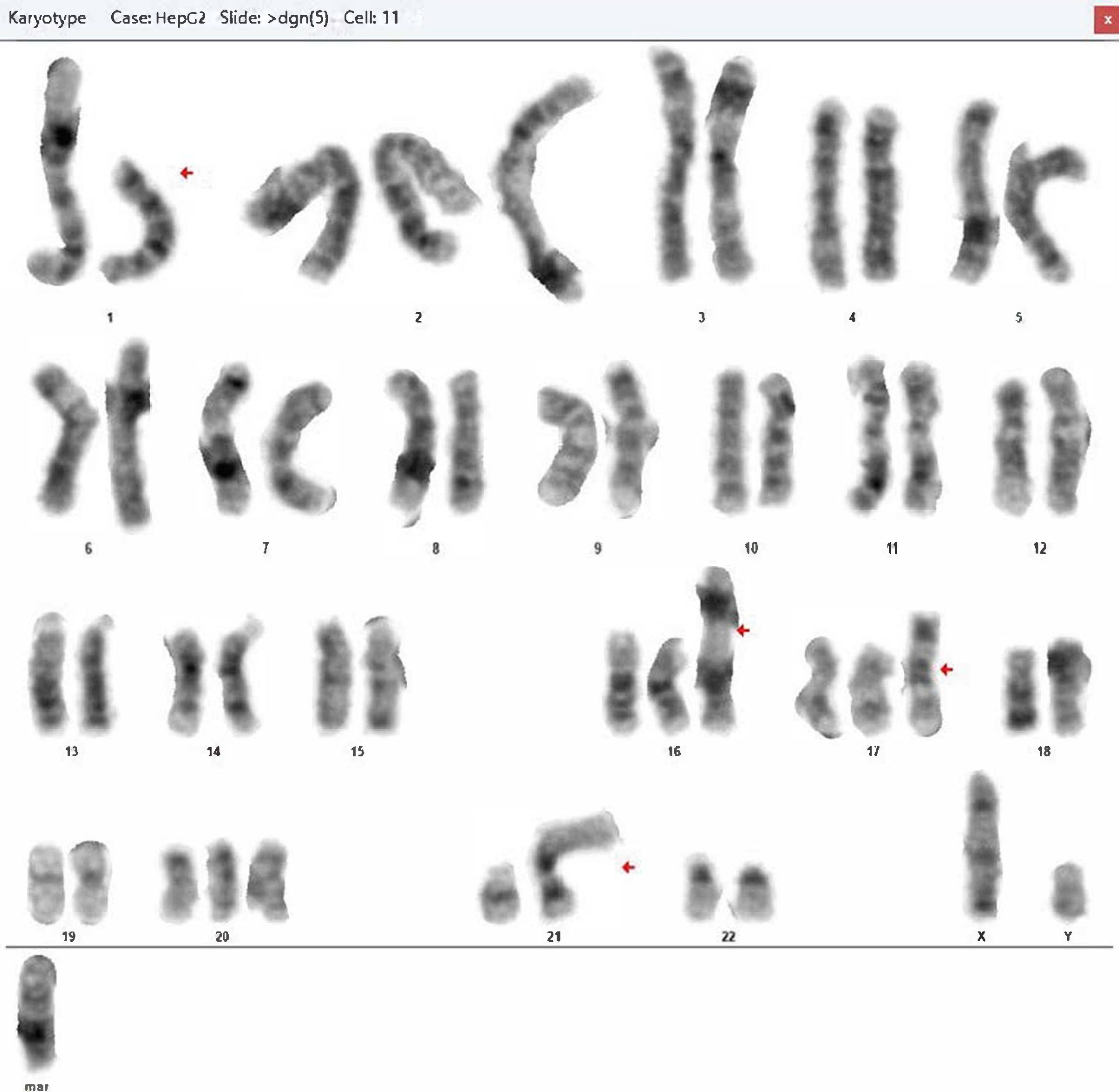
Karyotype
Case: HepG2
Slide: >dgn(5)
Cell: 11
1
2
3
4
5
6
7
8
9
10
11
12
13
14
15
16
17
18
19
20
21
22
X
Y
mar

Karyotype Case: HepG2 Slide: >dgn(5) Cell: 36

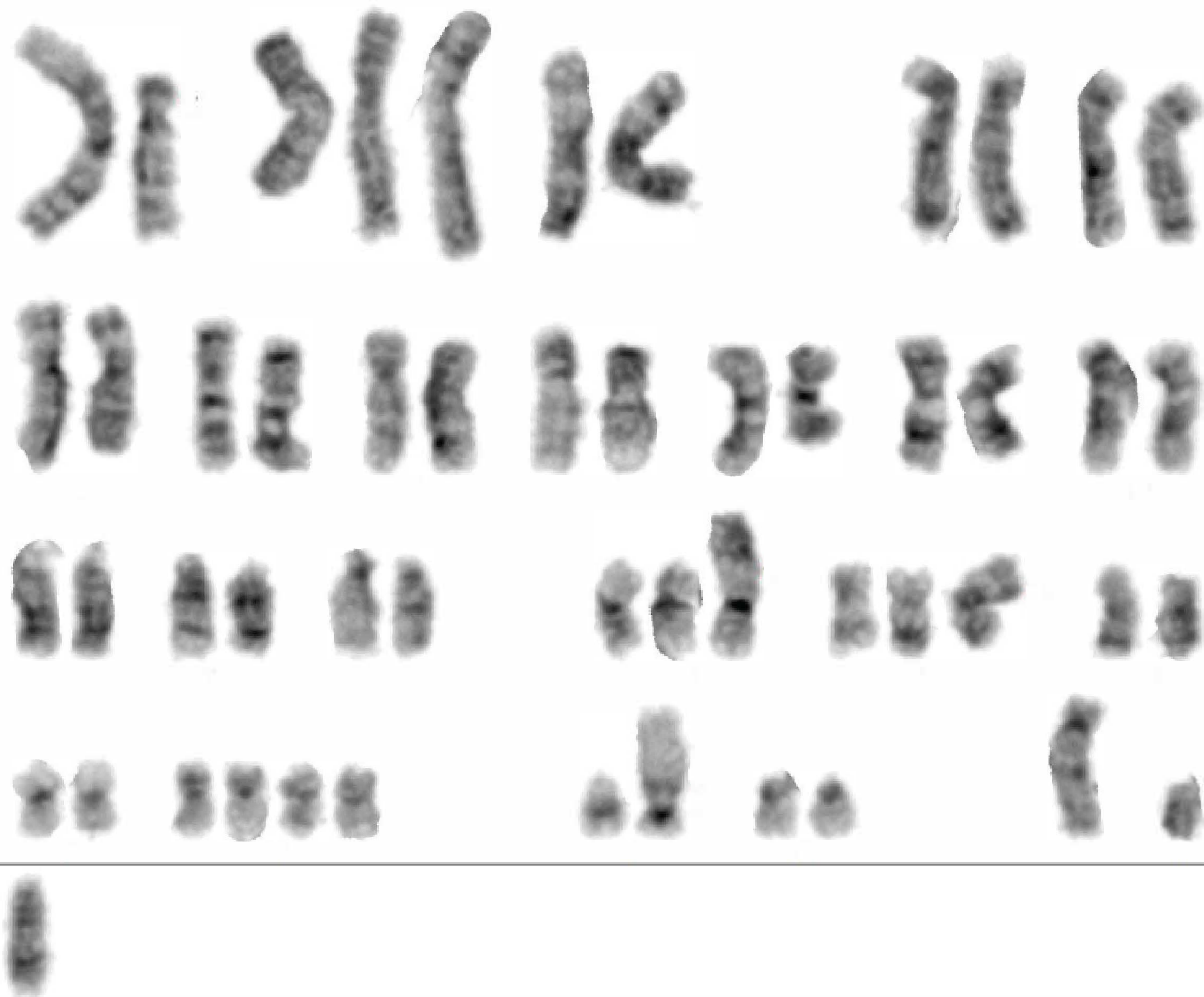

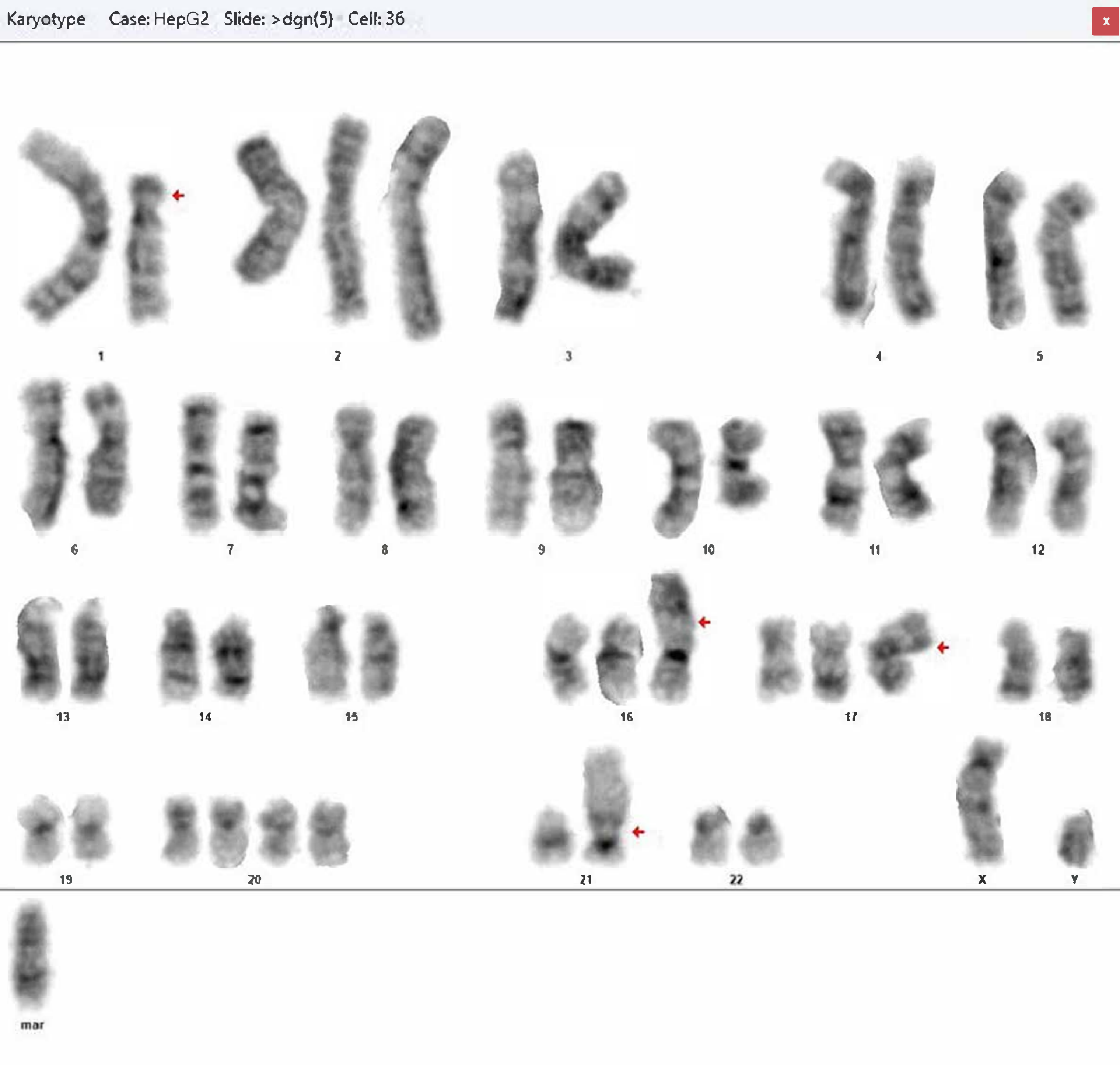
Karyotype Case: HepG2 Slide: >dgn(5) Cell: 36
x
1
2
3
4
5
6
7
8
9
10
11
12
13
14
15
16
17
18
19
20
21
22
X
Y
mar

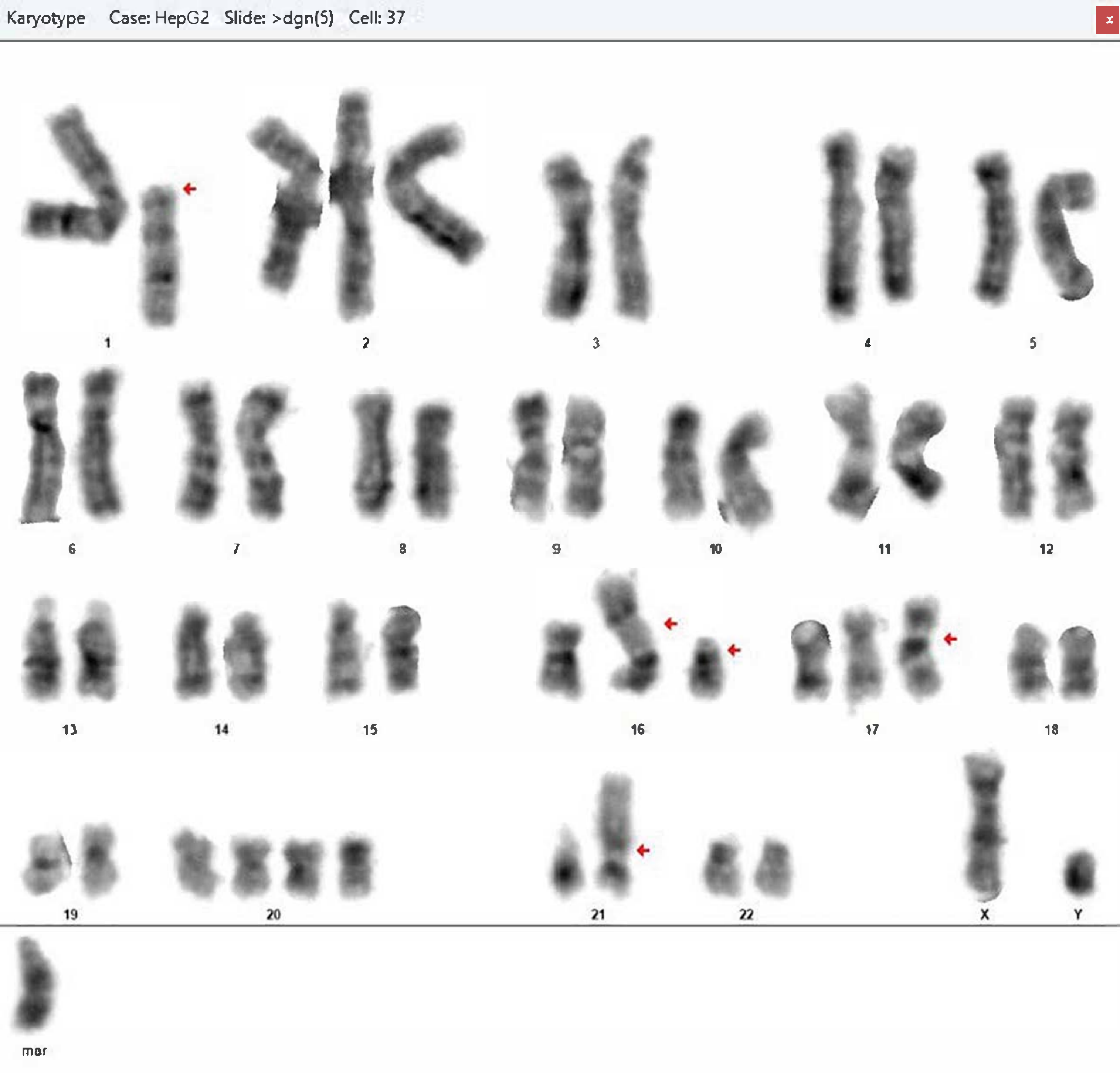
Karyotype
Case: HepG2
Slide: >dgn(5)
Cell: 37
1
2
3
4
5
6
7
8
9
10
11
12
13
14
15
16
17
18
19
20
21
22
X
Y
mar

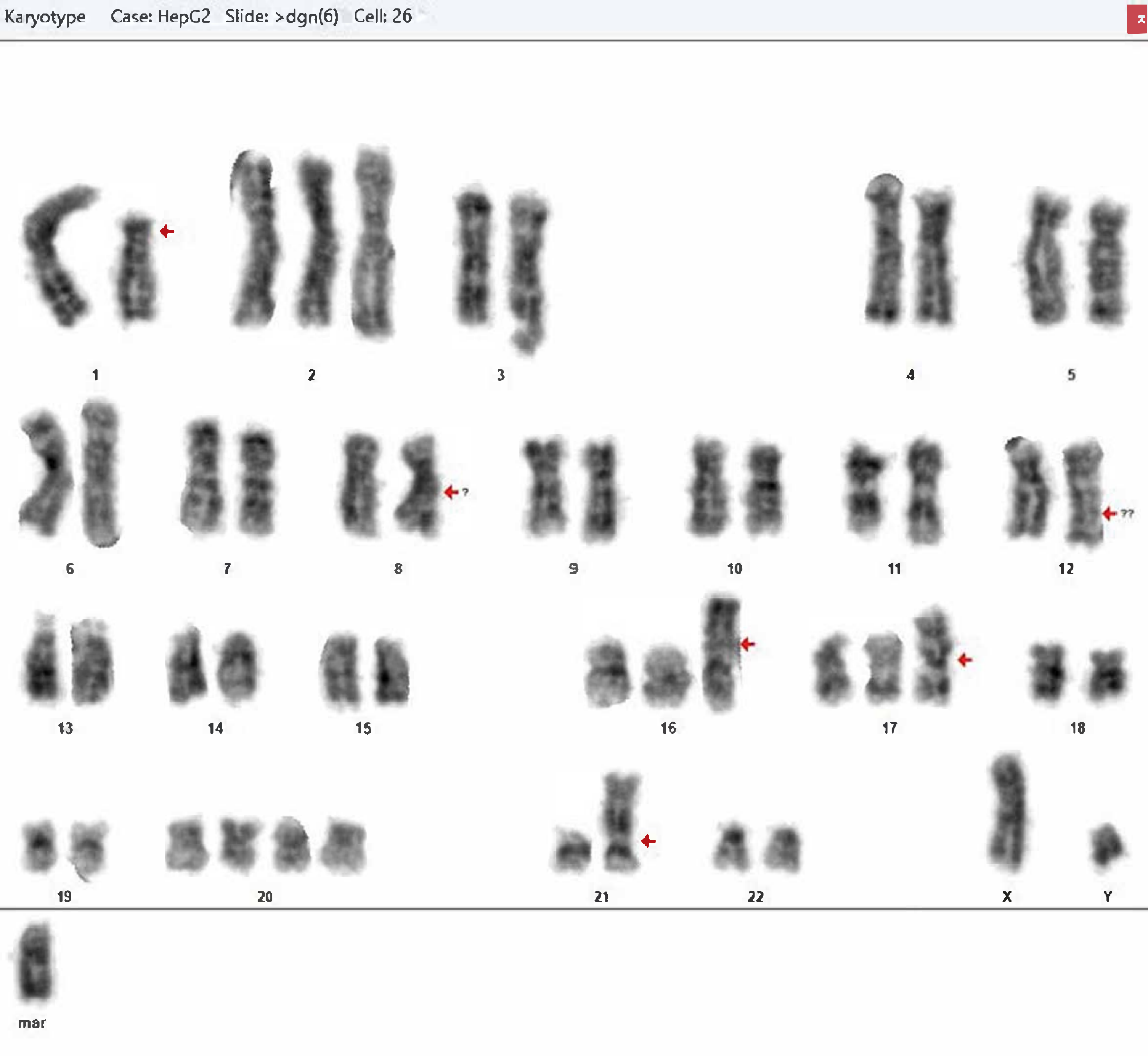
Karyotype Case: HepG2 Slide: >dgn(6) Cell: 26
1
2
3
4
5
6
7
8
?
9
10
11
12
??
13
14
15
16
17
18
19
20
21
22
X
Y
mar

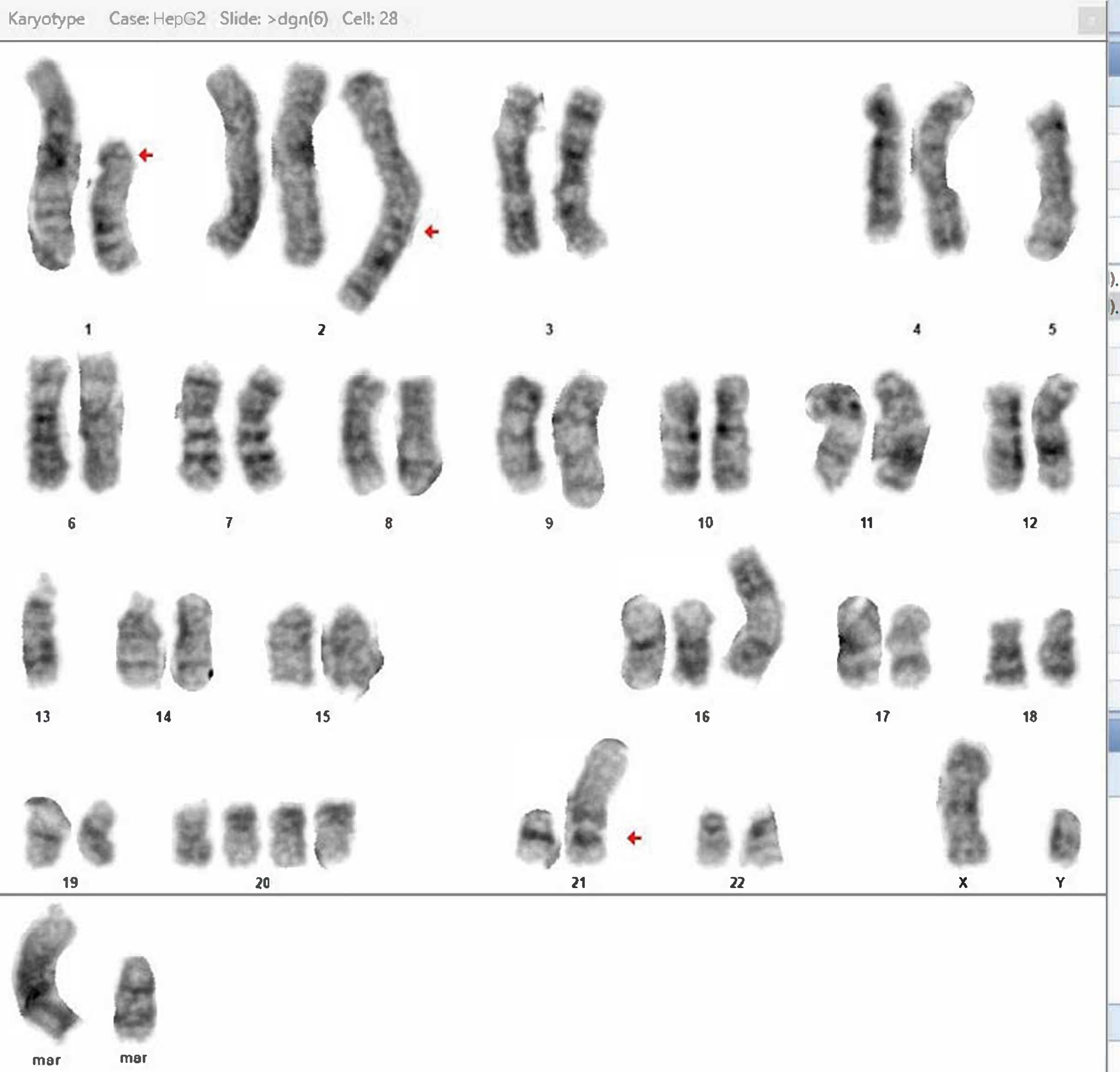
Karyotype
Case: HepG2
Slide: >dgn(6)
Cell: 28
1
2
3
4
5
6
7
8
9
10
11
12
13
14
15
16
17
18
19
20
21
22
X
Y
mar
mar

Case: Fa2N4

**Patient ID:**	**FA2N4**	**Operator Name:**	
**Patient Name:**	**Ellen-Victoria**	**Specimen:**	
**Date of Birth:**		**Received Date:**	**16-1-2025**
**Gender:**		**Completed:**	
**Clinical Indication:**			

Case Notes: **Analysis: 28-3-2025**
mar appears to be 20

Case Results:
line I:
43,X,-X,add(8)(p2?1),der(9;11)(q10;q10),-10,-13,-22,+2mar[cp3]

line II:
41~45,X,-X,add(8)(p2?1),-9,-10,del(11)(p11.2),-13,+17,-22,-22,+2mar[cp7]

Final Results:

Microscope ID	

Cells Information:

#	Label	Slide-Cell:	Analysis State	X , Y	State By	Report	Notes	Results
1	24h	2-9	Karyotyped	20,58 X 21,46	EV	None		II 45,XX,?4,add(8)(p2?1),-9,-10,del(11)(p11.2),-13,+17,-22,+2mar
2	24h	2-12	Karyotyped	11,36 X 25,70	EV	None		I 43,X,-X,add(8)(p2?1),der(9;11)(q10;q10),-10,-13,-22,+2mar
3	24h	2-13	Karyotyped	5,88 X 34,66	EV	None		I 43,XX,add(8)(p2?1),der(9;11)(q10;q10),-10,-13,-22,+mar
4	24h	2-16	Karyotyped	19,68 X 36,10	EV	None		II 43,XX,add(8)(p2?1),-10,add(11)(p11.2),add(12)([p12),-13,-18,-20,-22,+2mar
5	24h	2-25	Karyotyped	7,00 X 49,39	EV	None		II 41,XX,-8,add(8)(p2?1),-9,-10,del(11)(p11.2),-13,-19,-22,+mar
6	24h	7-2	Karyotyped	9,14 X 7,74	EV	None		II 42,X,-X,add(8)(p2?1),-9,-10,del(11)(p11.2),-13,-22,-22,+2mar
7	24h	7-18	Karyotyped	3,44 X 25,45	EV	None		II 43,X,-X,add(8)(p2?1),-9,-10,del(11)(p11.2),-13,+17,-22,-22,+2mar
8	24h	7-20	Karyotyped	8,22 X 32,32	EV	None		II 42,XX,add(8)(p2?1),-9,-10,del(11)(p11.2)-13,-16,-22,-22,+2mar
9	24h	7-24	Karyotyped	4,65 X 45,12	EV	None		I 43,X,-X,add(8)(p2?1),der(9;11)(q10;q10),-10,-13,-22,+2mar
10	24h	7-25	Karyotyped	4,31 X 45,09	EV	None		II 44,XX,add(8)(p2?1),-9,-10,del(11)(p11.2),-13,-22,+2mar

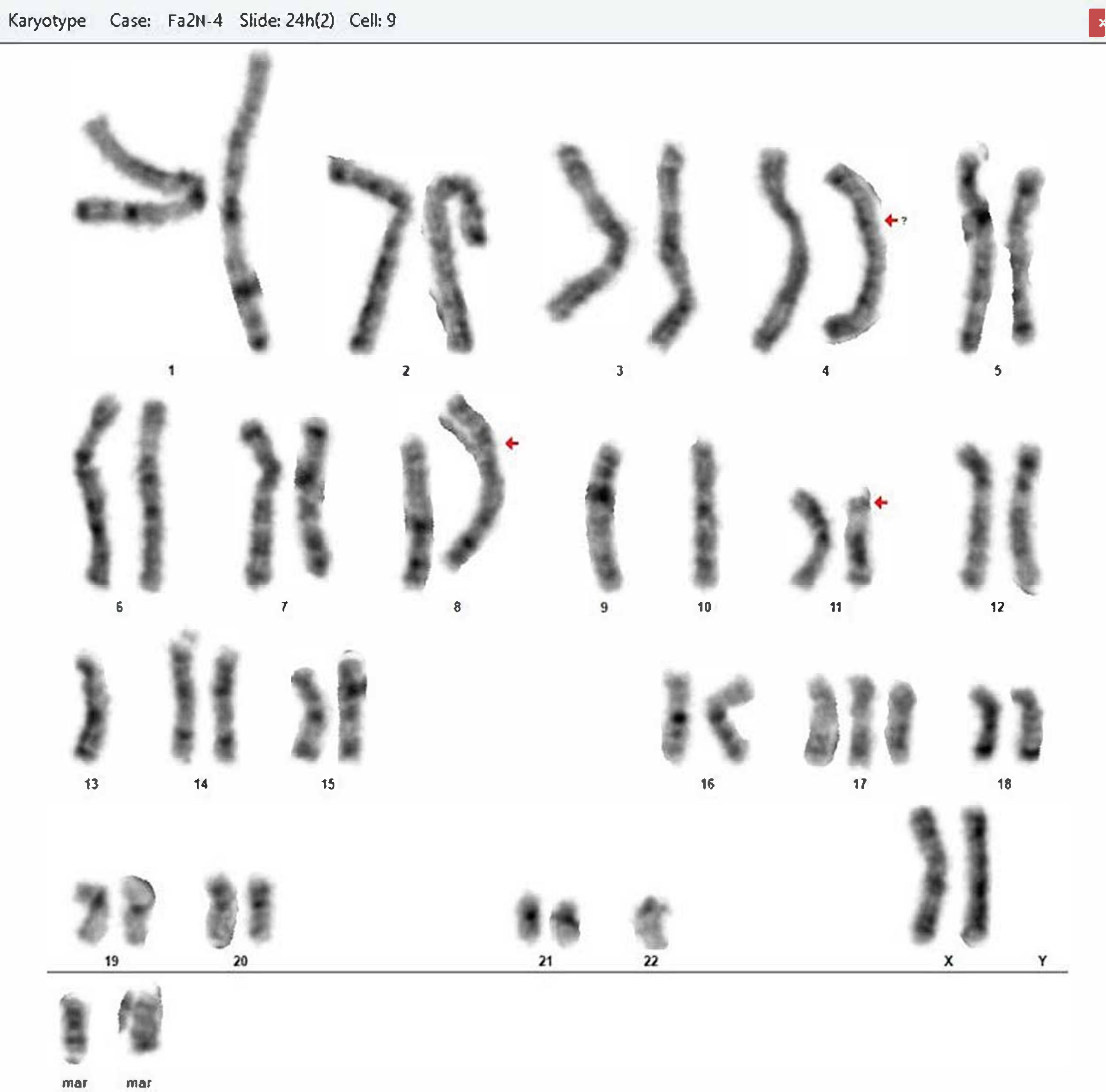
Karyotype Case: Fa2N-4 Slide: 24h(2) Cell: 9
1
2
3
4
?
5
6
7
8
9
10
11
12
13
14
15
16
17
18
19
20
21
22
X
Y
mar
mar

Karyotype Case: Fa2N-4 Slide: 24h(2) Cell: 12

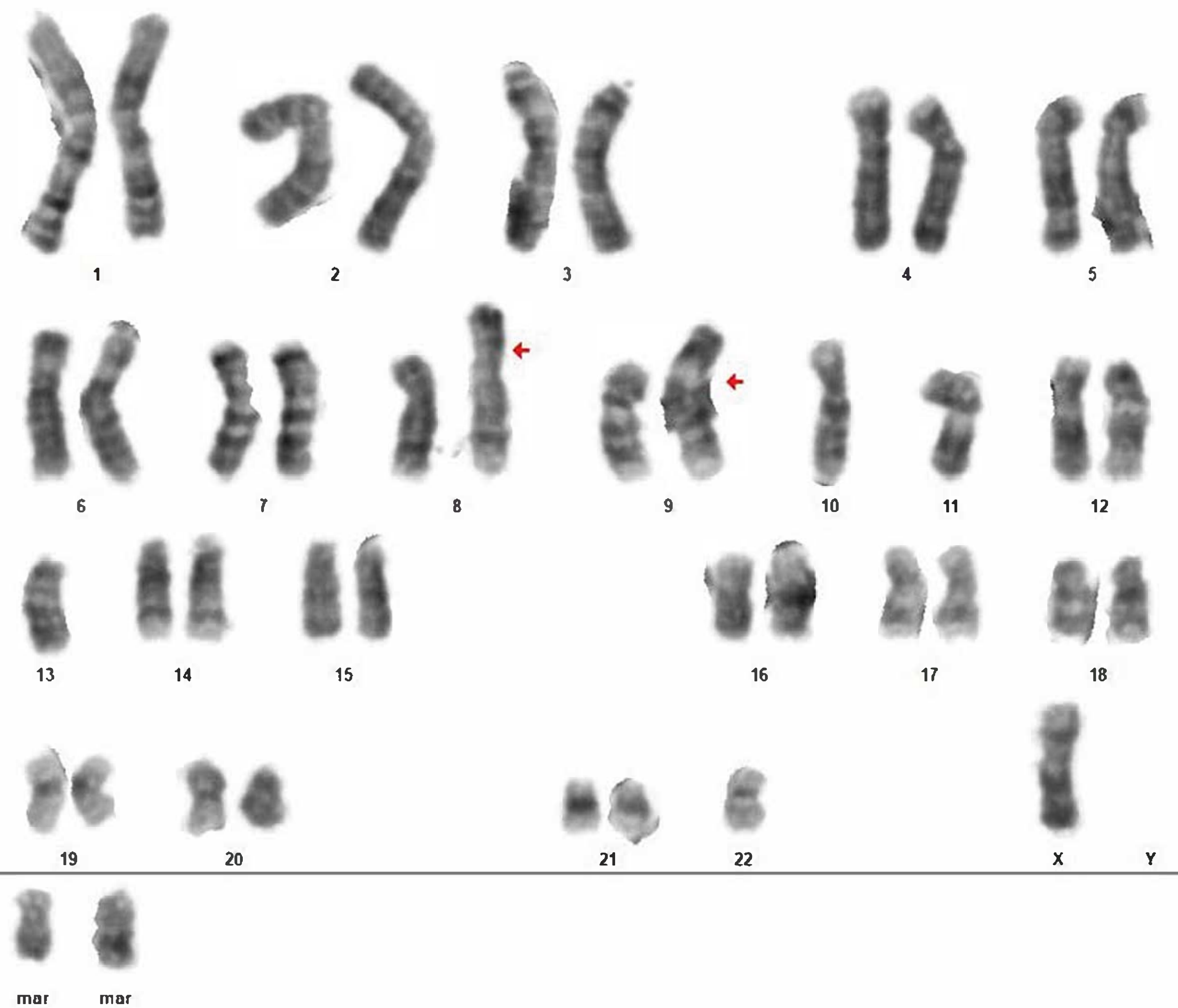

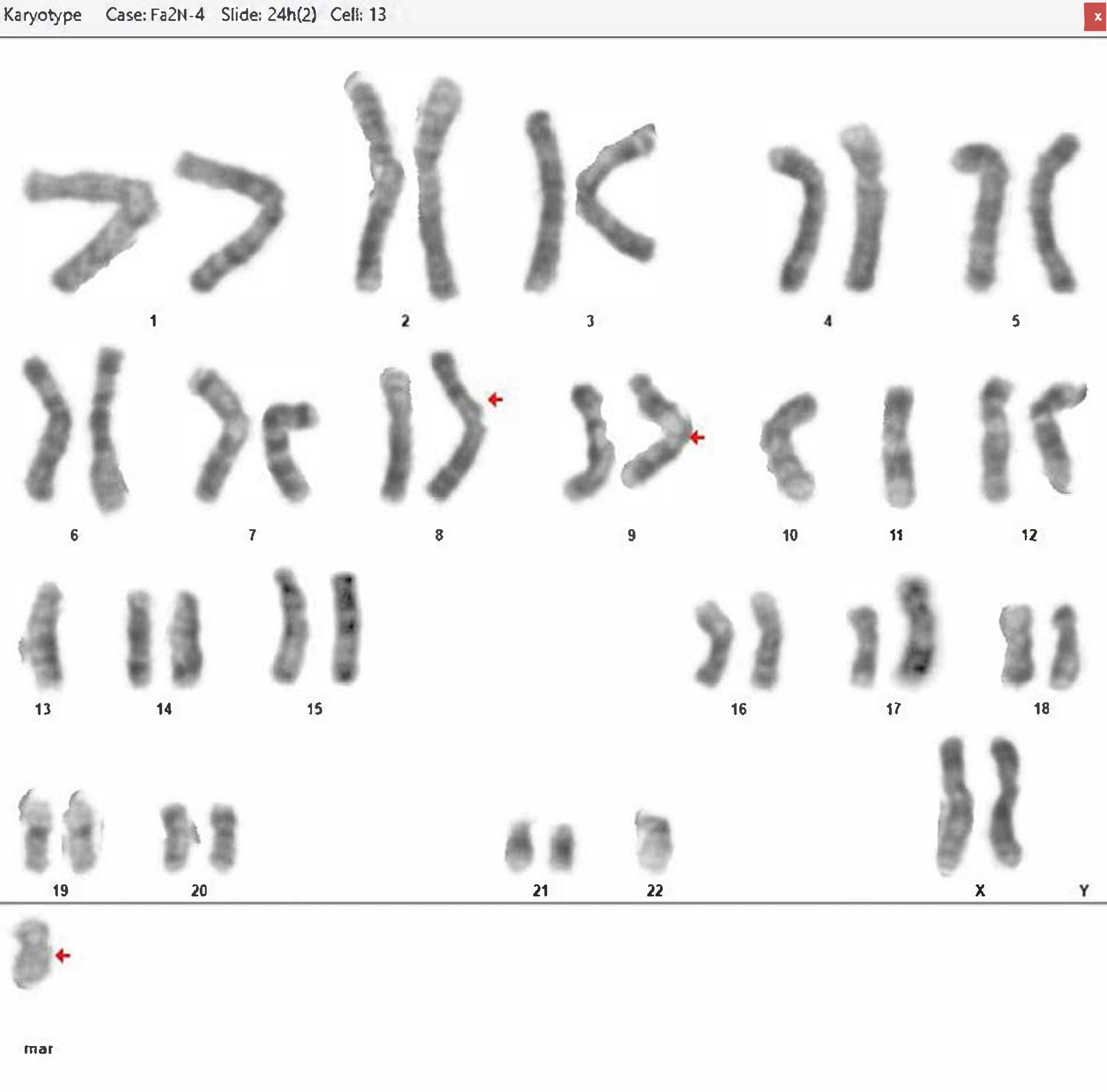
Karyotype Case: Fa2N-4 Slide: 24h(2) Cell: 13
x
1
2
3
4
5
6
7
8
9
10
11
12
13
14
15
16
17
18
19
20
21
22
X
Y
mar

Karyotype Case: Fa2N-4 Slide: 24h(2) Cell: 16

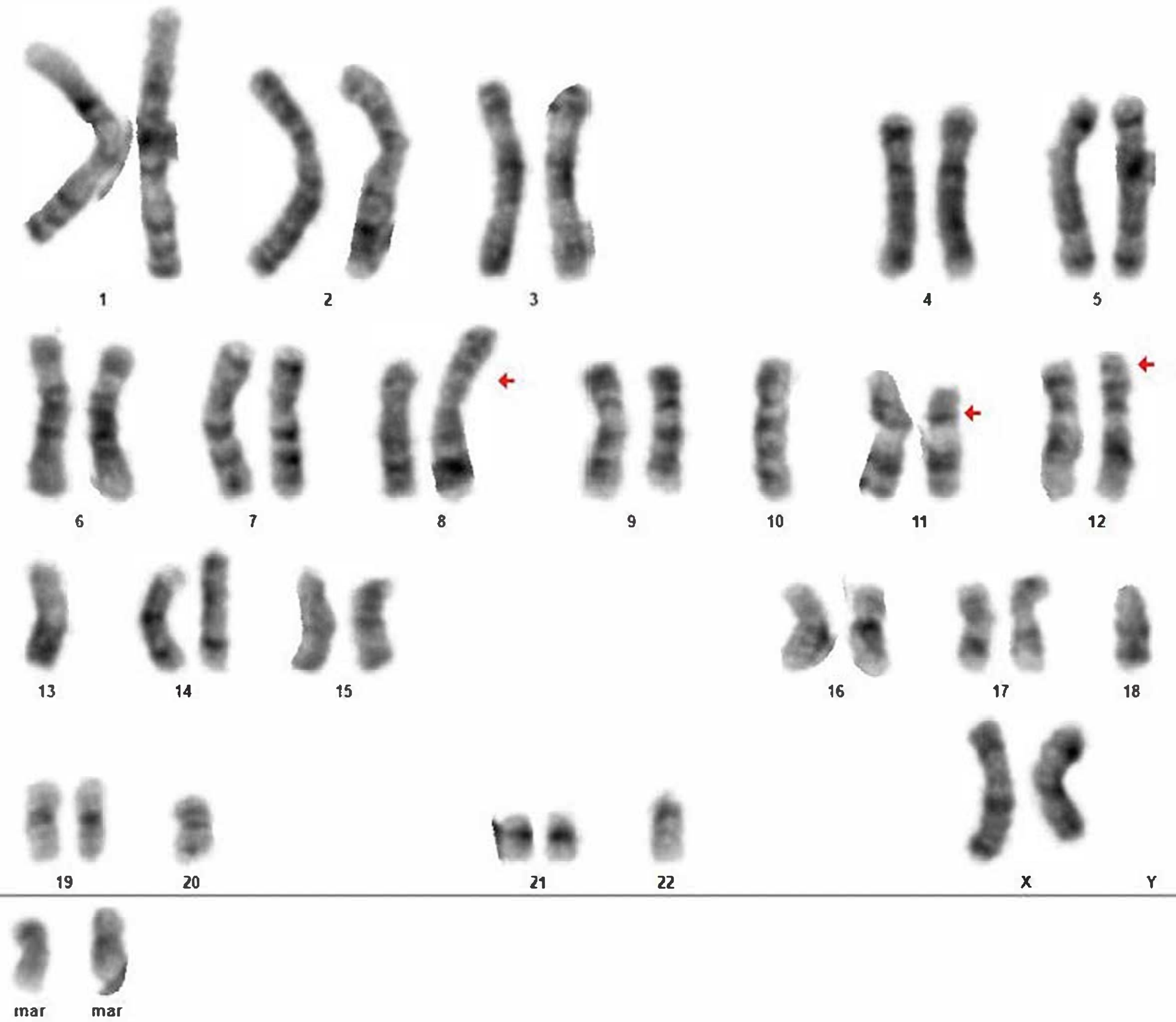

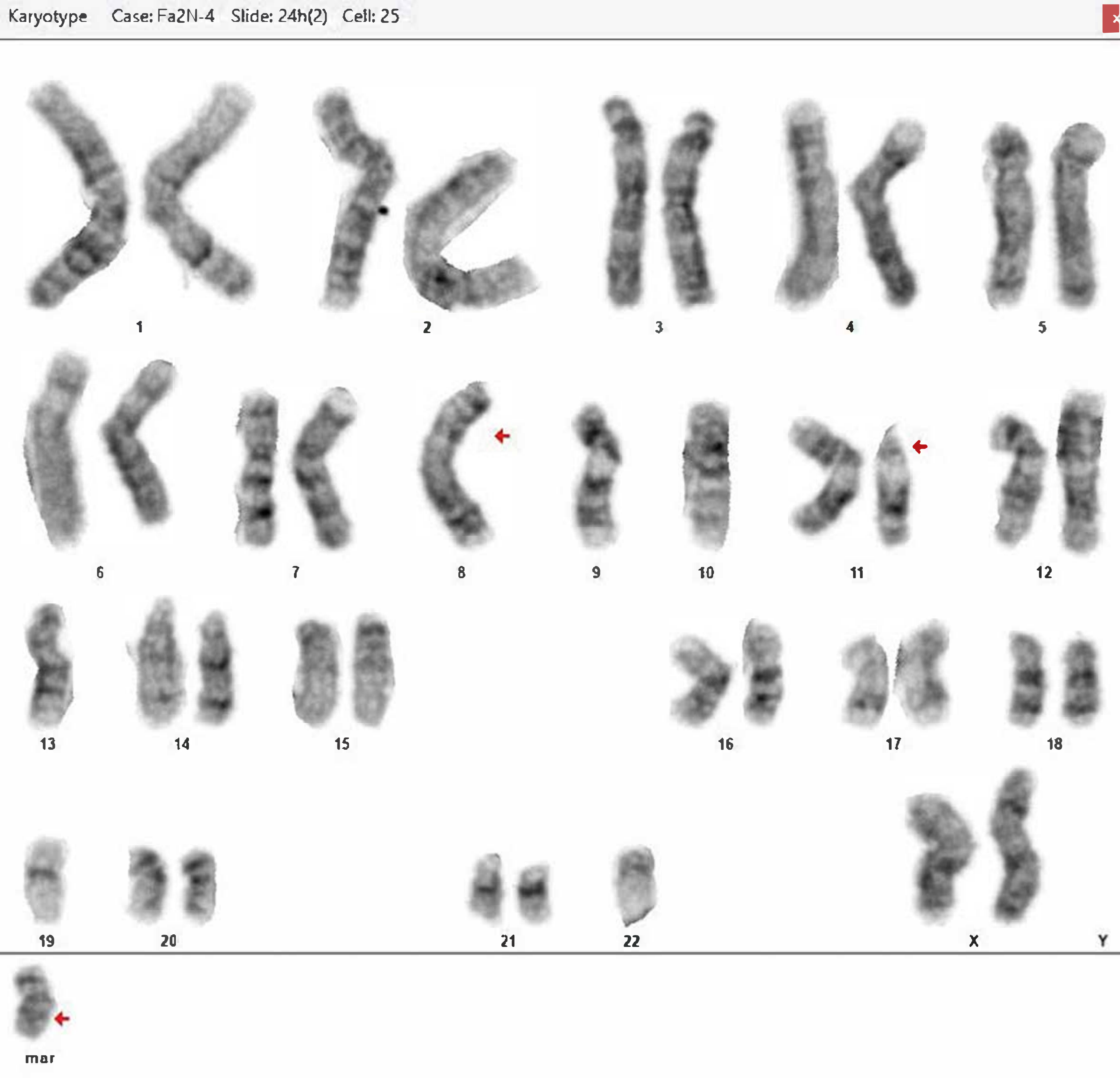
Karyotype Case: Fa2N-4 Slide: 24h(2) Cell: 25
1
2
3
4
5
6
7
8
9
10
11
12
13
14
15
16
17
18
19
20
21
22
X
Y
mar

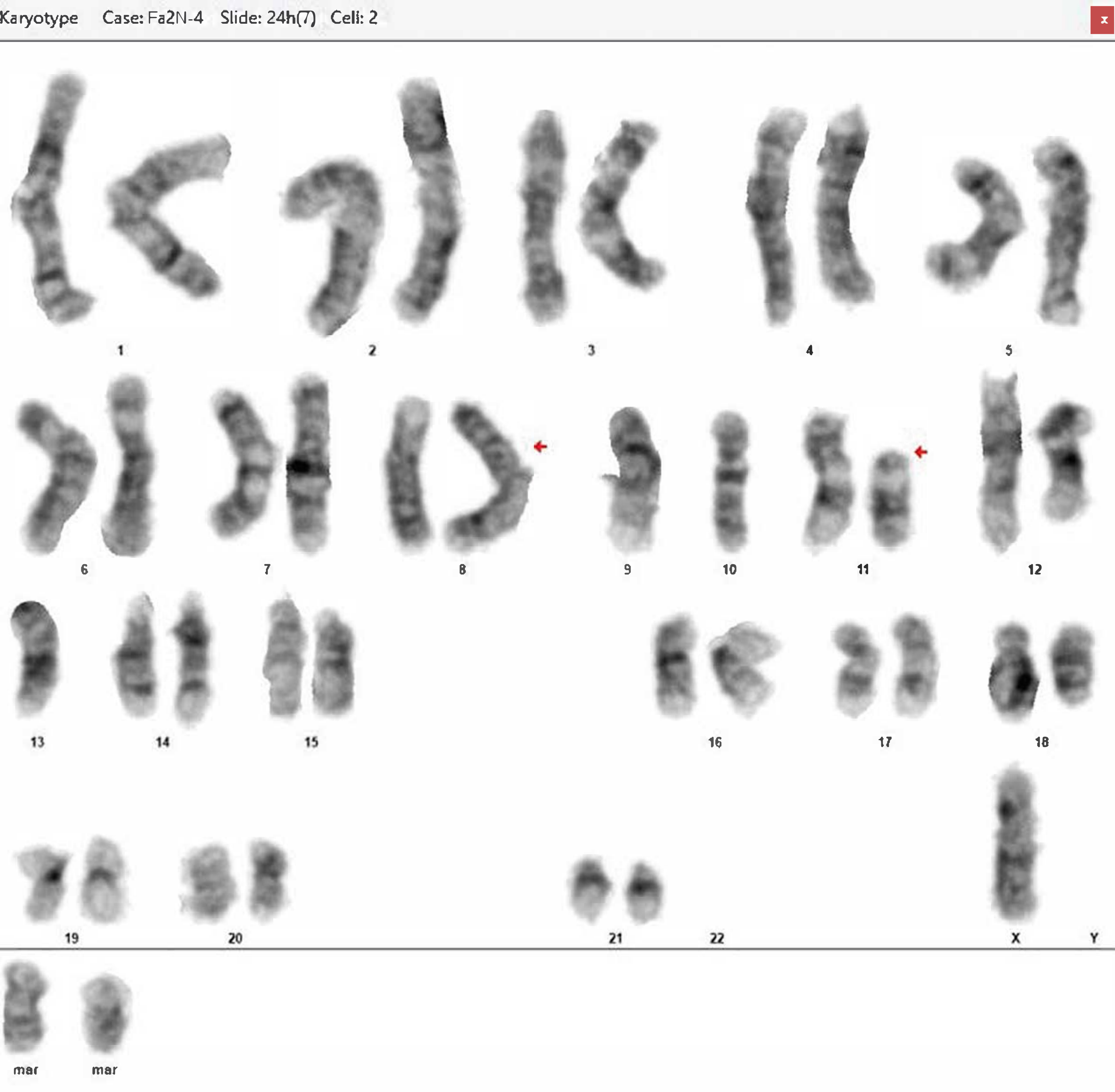
Karyotype
Case: Fa2N-4
Slide: 24h(7)
Cell: 2
1
2
3
4
5
6
7
8
9
10
11
12
13
14
15
16
17
18
19
20
21
22
X
Y
mar
mar

Karyotype Case: Fa2N-4 Slide: 24h(7) Cell: 18

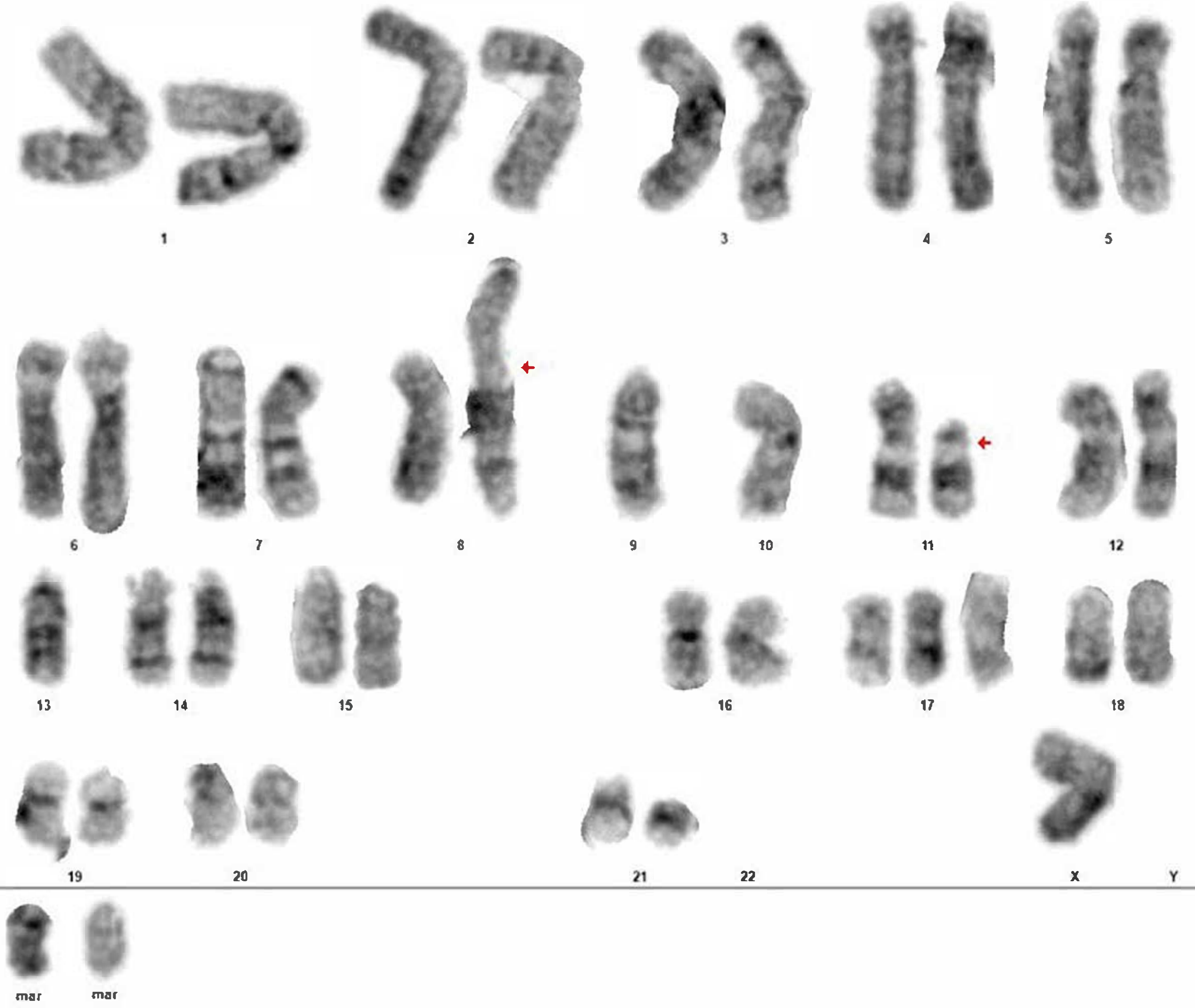

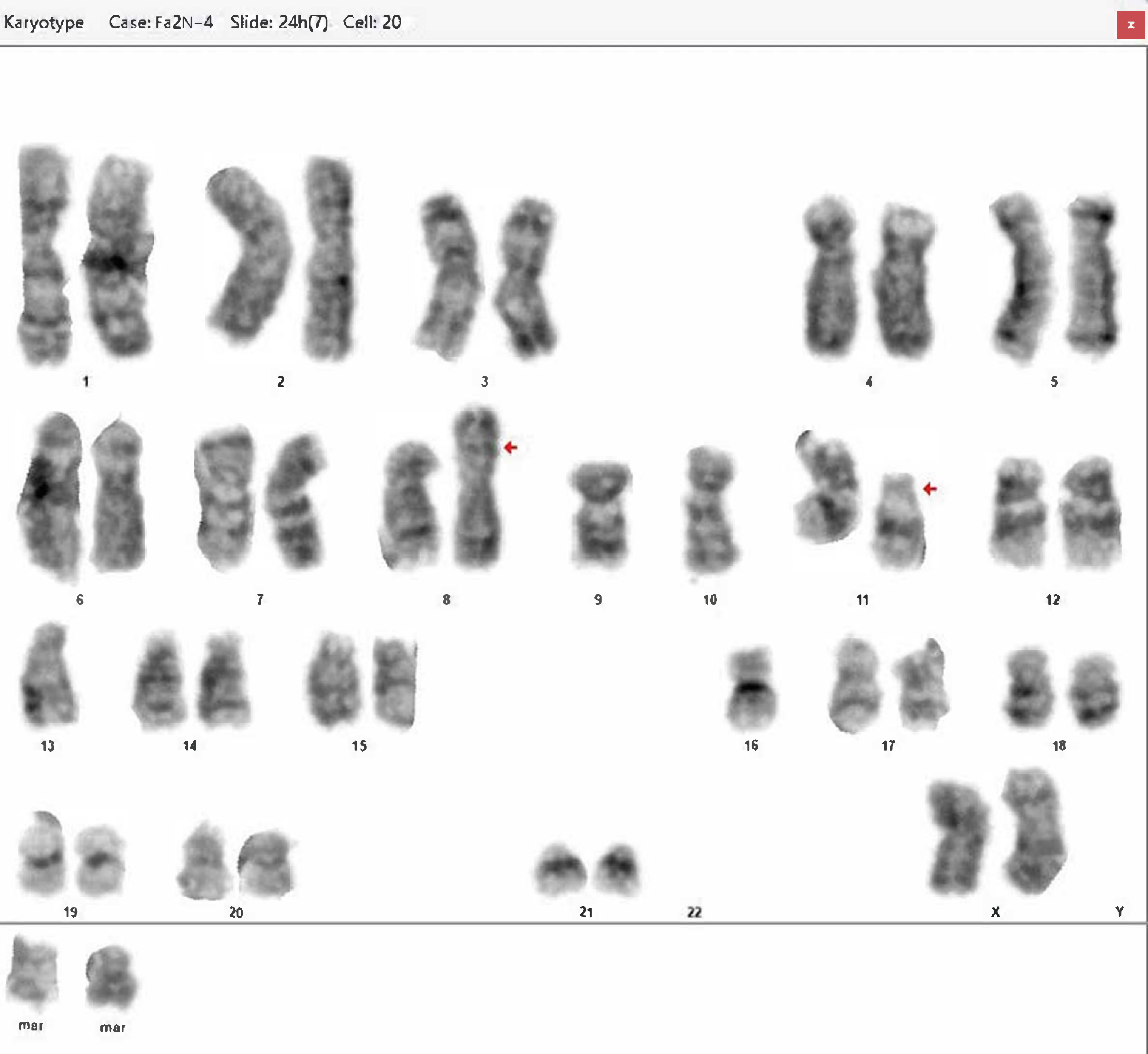
Karyotype Case: Fa2N-4 Slide: 24h(7) Cell: 20
1
2
3
4
5
6
7
8
9
10
11
12
13
14
15
16
17
18
19
20
21
22
X
Y
mar
mar

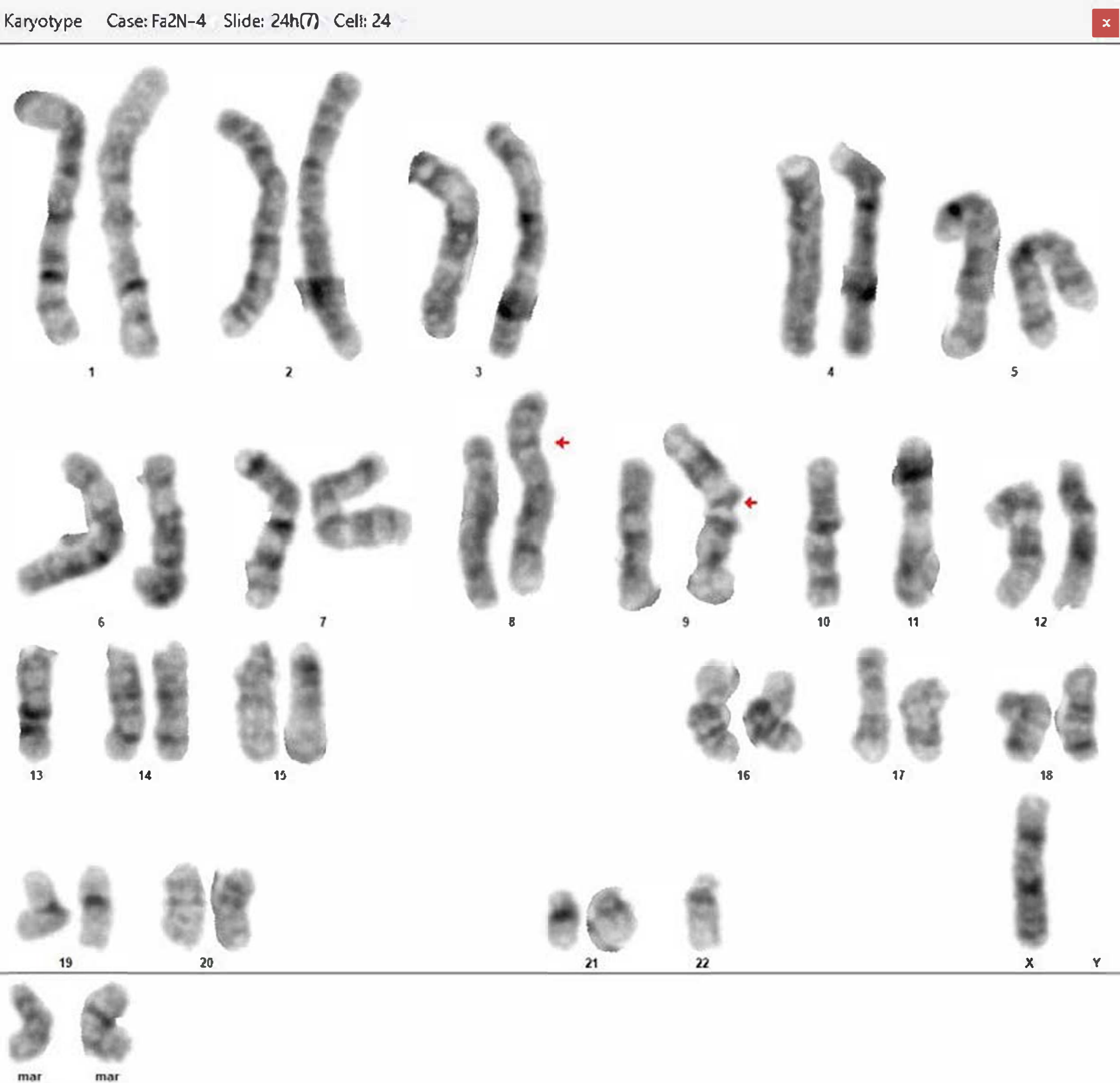
Karyotype Case: Fa2N-4 Slide: 24h(7) Cell: 24
1
2
3
4
5
6
7
8
9
10
11
12
13
14
15
16
17
18
19
20
21
22
X
Y
mar
mar

Karyotype Case: Fa2N-4 Slide: 24h(7) Cell: 25

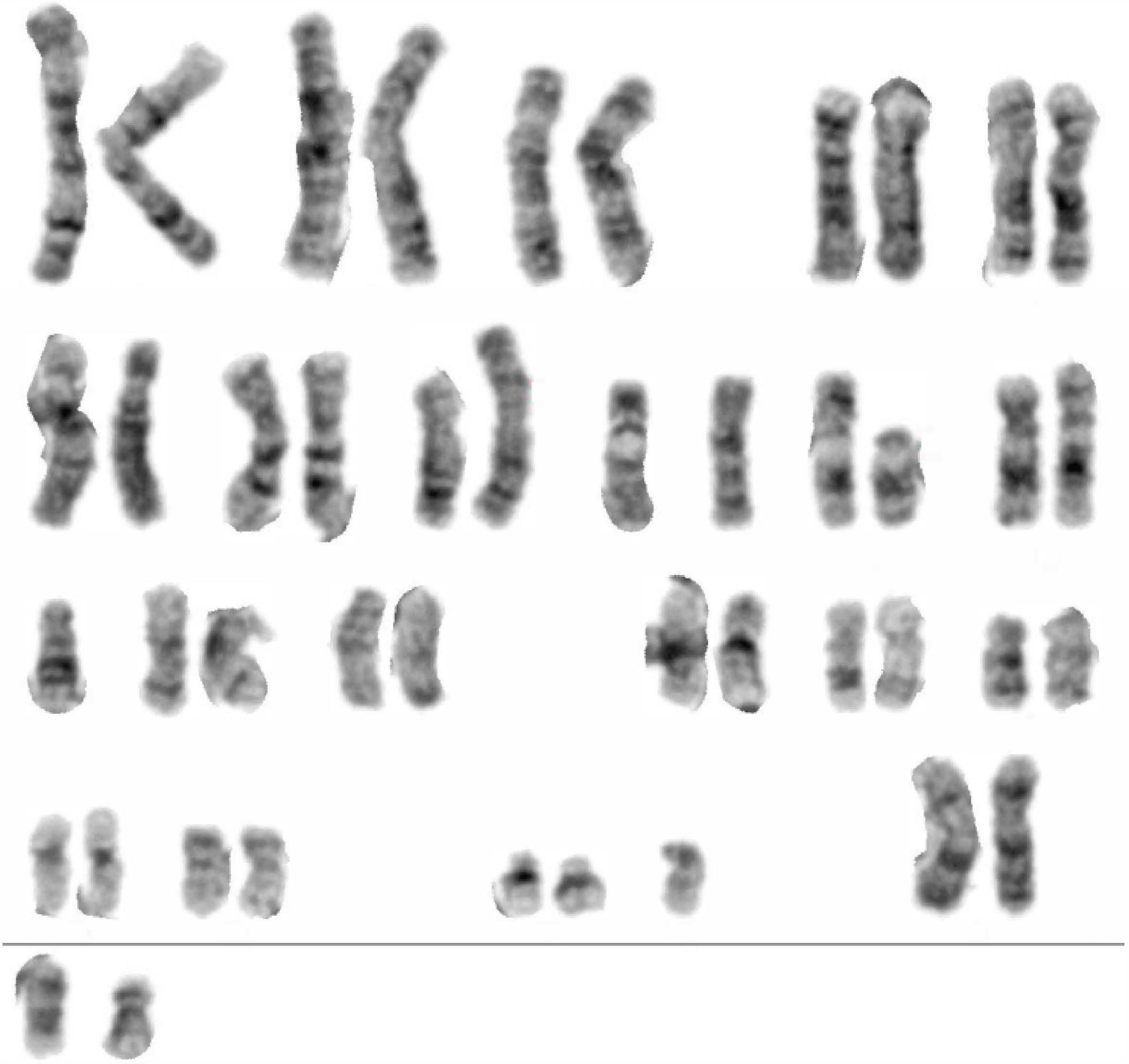

Karyotype Case: Fa2N-4 Slide: 24h(7) Cell: 25

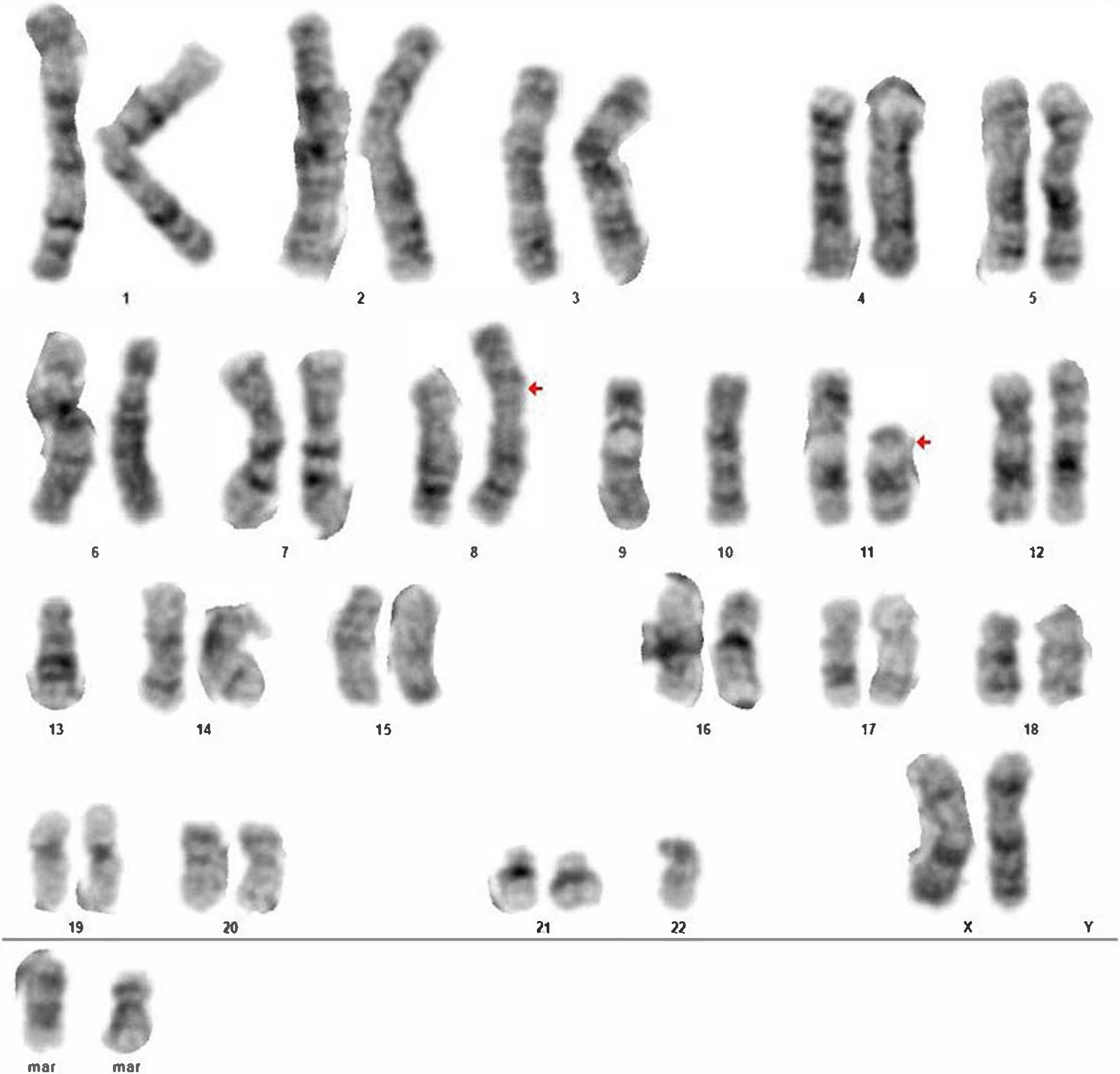

Supplement: Supplementary file 2 [file mmc3.pdf]
